# *Magnaporthe oryzae* fimbrin organizes actin networks in the hyphal tip during polar growth and pathogenesis

**DOI:** 10.1371/journal.ppat.1008437

**Published:** 2020-03-16

**Authors:** Yuan-Bao Li, Rui Xu, Chengyu Liu, Ningning Shen, Li-Bo Han, Dingzhong Tang

**Affiliations:** 1 State Key Laboratory of Ecological Control of Fujian-Taiwan Crop Pests, Key Laboratory of Ministry of Education for Genetics, Breeding and Multiple Utilization of Crops, Plant Immunity Center, Fujian Agriculture and Forestry University, Fuzhou, Fujian, China; 2 College of Agriculture, Fujian Agriculture and Forestry University, Fuzhou, Fujian, China; 3 College of Life Sciences, Fujian Agriculture and Forestry University, Fuzhou, Fujian, China; Sainsbury Laboratory, UNITED KINGDOM

## Abstract

*Magnaporthe oryzae* causes rice blast disease, but little is known about the dynamic restructuring of the actin cytoskeleton during its polarized tip growth and pathogenesis. Here, we used super-resolution live-cell imaging to investigate the dynamic organization of the actin cytoskeleton in *M*. *oryzae* during hyphal tip growth and pathogenesis. We observed a dense actin network at the apical region of the hyphae and actin filaments originating from the Spitzenkörper (Spk, the organizing center for hyphal growth and development) that formed branched actin bundles radiating to the cell membrane. The actin cross-linking protein Fimbrin (MoFim1) helps organize this actin distribution. MoFim1 localizes to the actin at the subapical collar, the actin bundles, and actin at the Spk. Knockout of *MoFim1* resulted in impaired Spk maintenance and reduced actin bundle formation, preventing polar growth, vesicle transport, and the expansion of hyphae in plant cells. Finally, transgenic rice (*Oryza sativa*) expressing RNA hairpins targeting *MoFim1* exhibited improved resistance to *M*. *oryzae* infection, indicating that *MoFim1* represents an excellent candidate for *M*. *oryzae* control. These results reveal the dynamics of actin assembly in *M*. *oryzae* during hyphal tip development and pathogenesis, and they suggest a mechanism in which MoFim1 organizes such actin networks.

## Author summary

The actin cytoskeleton plays crucial roles in *M*. *oryzae* growth and pathogenesis. However, the organizing principles and dynamic behavior of the cytoskeleton in *M*. *oryzae* growth and expansion in plant cells remain enigmatic. In this study, through super-resolution observation, we found a dense network of actin cables dynamically gathered near the Spitzenkörper, the organizing center for hyphal growth and development. The actin cytoskeleton also accumulated at the narrow penetration site where the hypha invaded the plant cell. Furthermore, the actin cross-linking protein MoFim1 contributes to organizing these actin cables, thus facilitating hyphal polarity, vesicle transport, and invasive expansion of hyphae in the host plant cell. In all, our results uncovered a mechanism by which *M*. *oryzae* organizes and relies on a unique actin distribution pattern in hyphae during cell growth and pathogenesis.

## Introduction

*Magnaporthe oryzae* is a filamentous fungus and the causal agent of rice blast disease. Each year, infections caused by this recalcitrant pathogen destroy up to 30% of the rice (*Oryza sativa*) crop and threaten global food security [1, 2]. *M*. *oryzae* undergoes extensive developmental changes while building elaborate infection structures, invading plant cells, and finally proliferating inside the host cells [3, 4]. Thus, understanding the biology of *M*. *oryzae* development and infection is critical for developing durable strategies for controlling rice blast disease.

In filamentous fungi, hyphae generally extend by tip growth [5, 6]. This process is thought to be associated with the polarized trafficking of secretory vesicles to the Spitzenkörper (Spk), the organizing center for hyphal growth and development [7]. Cell tip growth determination and protein secretion are intriguing issues in fungal cell biology [6]. Polarized exocytosis is an essential process in fungi and is involved in cell morphogenesis and pathogenesis [8, 9]. The Spk, which acts as a vesicle supply center, is composed of a dense group of vesicles, cytoskeleton, ribosomes, and other undefined components delivered in a polar manner [7, 10, 11]. The exocyst complex in *M*. *oryzae* is located at the hyphal tip, ahead of the Spk, indicating that a successive protein secretion pathway is required for fungal infection [12]. However, even though the Spk plays critical roles in the development and pathogenesis of filamentous fungi, how the Spk is maintained and moves in a polar manner in fungal cells is not well understood.

Actin is a highly conserved component of the cytoskeleton found in all eukaryotes and plays important roles in a variety of cellular processes [13, 14]. The main components of the actin cytoskeleton include monomeric globular actin (G-actin), polymeric filamentous actin (F-actin), motors, actin-binding proteins, and septins (which serve as actin scaffolds) [15]. During the past decades, much progress has been made in elucidating the crucial roles of the actin cytoskeleton in fungal morphogenesis and the pathogenesis of *M*. *oryzae*. For example, the *M*. *oryzae* actin motor proteins MoMyo2 and MoMyo5 are associated with hyphal growth and appressorial development; thus, their mutants exhibit defects in the infection of rice host cells [16–18]. The actin-binding proteins MoCAP and MoEnd3 are key factors in *M*. *oryzae* development and virulence through regulating actin assembly [19, 20]. *M*. *oryzae* septins polymerize into a heteromeric ring to scaffold a toroidal F-actin network at the base of the appressorium to facilitate the formation of a rigid penetration peg, which breaches the leaf surface [21]. Pmk1, a fungal mitogen-activated protein kinase (MAPK), was recently found to regulate the expression of the actin cross-linking protein alpha-actinin, and the septin phosphorylation protein Chm1, thus controlling the hyphal constriction required for the fungus to penetrate from one rice cell into its neighbor [22]. These findings shed light on the importance of the actin cytoskeleton in *M*. *oryzae* development and pathogenesis. However, despite this progress, live-cell visualization is needed to explore the dynamic remodeling of the actin cytoskeleton in *M*. *oryzae* during typical cellular processes, including development and infection, to understand its versatile functions.

Fimbrin is a conserved F-actin cross-linking protein present in organisms ranging from yeast to mammals and plays important roles in numerous fundamental cellular processes. Fission yeast fimbrin (Sac6) is reported to play important roles in endocytosis, cytokinesis, and polarization [23, 24]. Five *FIMBRIN* genes are present in the *Arabidopsis thaliana* genome [25, 26]. Among the encoded fimbrins, FIMBRIN1 and FIMBRIN5 have been implicated in the regulation of pollen development and FIMBRIN5 might regulate the generation and maintenance of the rigidity of actin bundles oriented along the length of the pollen tube [26–28]. In addition, GFP-ABD2, comprising the second actin-binding domain (ABD2) of Arabidopsis FIMBRIN1 fused with green fluorescent protein (GFP), is widely used as an actin-labeling probe in plants [29–31].

Compared to our understanding of fimbrin functions in plants, our knowledge of the physiological functions of fimbrin in filamentous fungi is limited. A fimbrin protein in *Aspergillus nidulans* was observed as mobile patches throughout the hyphae and concentrated near hyphal apices, which are thought to play a role in endocytosis [32]. A *M*. *oryzae* fimbrin protein was previously reported to accumulate in dynamically moving actin patches at the hyphal subapical region and function by interacting with the exocyst [12]. However, neither the mechanisms by which filamentous fungal fimbrins regulate the dynamic organization of actin filaments, nor the associated underlying cellular processes, are well understood. Addressing these issues would help unravel exactly how fimbrin regulates the growth and pathogenesis of fungal pathogens, increasing our understanding of the functions of the actin cytoskeleton in filamentous fungi.

In this study, we labeled the actin cytoskeleton in the model pathogenic fungus *M*. *oryzae* using the actin probe Lifeact-GFP [33, 34] and performed super-resolution live-cell imaging to reveal the architecture and dynamics of the actin cytoskeleton during polar growth and vesicle delivery in fungal hyphae. We demonstrate that the actin cytoskeleton forms a unique structure at the apex of the active hyphae, including actin at the Spk and the formation of branched actin cables originating from the Spk and extending into the cell membrane. Furthermore, we show that *M*. *oryzae* Fimbrin (MoFim1) helps maintain this elaborate actin organization. Our results suggest that the development and elaboration of apical actin structures help ensure vesicle delivery for fungal development and protein secretion as well as hyphal expansion in rice cells. In addition, this study increases our understanding of the actin-based regulatory mechanism underlying both the development and pathogenesis of filamentous fungi.

## Results

### MoFim1 is important for the polar growth, conidiation, and full virulence of *M*. *oryzae*

**Fig 1:**
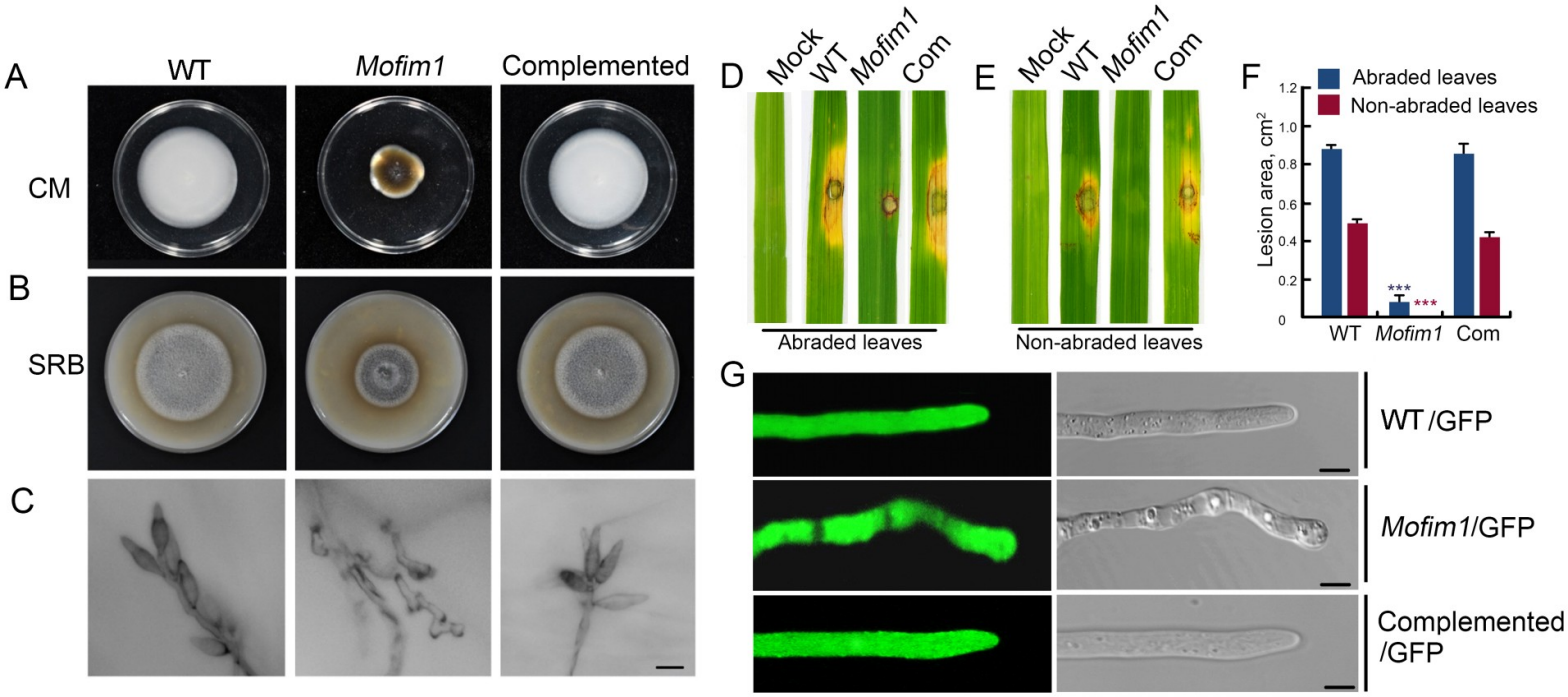
Growth, conidiation, hyphal morphogenesis, and plant infection defects of the *Mofim1* mutant. Seven-day-old cultures of WT, *Mofim1*, and the complemented strain on CM **(A)** and SRB medium **(B)**. **(C)** Microscopy observation of conidiation in WT, *Mofim1*, and the complemented strain. Bar = 10 μm. Pathogenicity assay using abraded **(D)** and non-abraded rice leaves **(E)** of WT, *Mofim1*, and the Com (complemented) strain. The same area of each SRB culture plate from WT, *Mofim1*, and the complemented strain was used to infect these rice leaves (*O*. *sativa* cv. Nipponbare). Photographs were taken 5 d after infection. **(F)** Quantification of the lesion area of the rice leaves shown in **(D)** and **(E)**. Error bars represent SD (*n* = 20) and asterisks (***) represent significant difference (*P* < 0.001). **(G)** Hyphal phenotypes of GFP-labeled WT, *Mofim1*, and the complemented strain. The hyphae were cultured in CM, and 100 hyphae were analyzed for WT, *Mofim1*, and the complemented strain. Bars = 5 μm.

The physiological roles of fimbrins in regulating actin rearrangement during *M*. *oryzae* development and pathogenesis have not been elucidated. The *M*. *oryzae* Y34 genome contains one *Fimbrin* gene, *MoFim1*. To investigate the physiological functions of MoFim1, we employed the standard one-step gene replacement strategy to knock out *MoFim1* in *M*. *oryzae* [35] (S1 Fig). When cultured on complete medium (CM) or straw rice bran (SRB) medium, the *Mofim1* knockout mutant developed at a significantly lower rate compared to the wild type (WT) and complemented strain (Fig 1A and 1B, S2 Fig). Furthermore, conidiation was completely abolished in the *Mofim1* mutant. No mature spores were present on the conidiophores, as observed by microscopy (Fig 1C).

Because the mutant fails to produce mature spores, we could not carry out single-spore isolation. Instead, we generated protoplasts using three *Mofim1* deletion clones. We diluted and cultured the protoplasts on CM agar medium, then picked and cultured a single colony from each clone. We found that all colonies exhibited the same developmental phenotype as the *Mofim1* mutant. Furthermore, all the single colonies could be recovered to a wild-type phenotype by expressing *MoFim1* driven by its native promoter (S3A Fig). We also performed a Southern blot experiment to examine these mutants, which showed that these colonies were true deletion mutants (S3B Fig).

We next analyzed the pathogenesis of *Mofim1* using the same region on the plate for SRB-grown cultures of WT, *Mofim1*, and the complemented strain to infect both abraded and non-abraded rice leaves (*Oryza sativa* cv. Nipponbare). We found that deletion of *MoFim1* severely affected the ability of *M*. *oryzae* to infect rice leaves. Comparing with the WT and complemented strains, the *Mofim1* mutant induced a rather small lesion on the abraded rice leaves (Fig 1D and 1F). On the non-abraded leaves, we could not find evidence for penetration and infection by the mutant (Fig 1E and 1F).

Microscopy of the hyphae revealed that while almost all WT and complemented tips maintained the hyphoid shape, the *Mofim1* mutant had more blunt ends (Fig 1G). These results indicate that MoFim1 plays important roles in the development and pathogenesis of *M*. *oryzae*.

### MoFim1 helps organize the actin cytoskeleton in the hyphal tip

**Fig 2:**
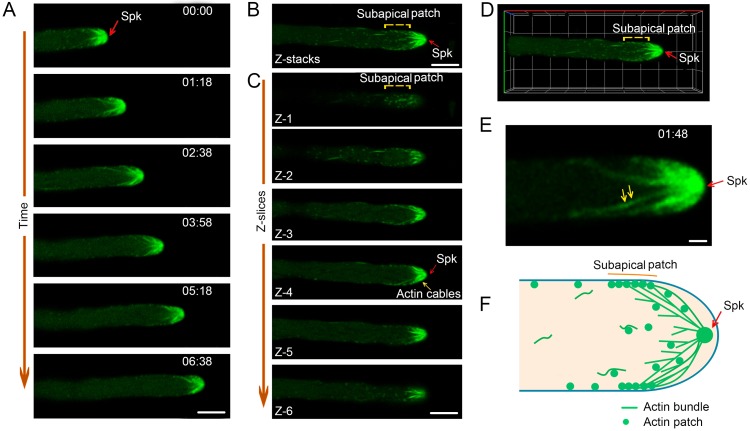
Organization of the actin cytoskeleton at the growing hyphal tip. **(A)** Time-lapse images illustrating actin distribution in the tip region of a growing hypha in liquid CM. The corresponding movie is provided as S1 Movie, which contains 200 frames. The numbers at the top right corner indicate the timestamps (min:s). Bar = 5 μm. **(B)** Maximum projection of Z-slices obtained by imaging a hyphal tip illustrating the structure of the actin cytoskeleton. Bar = 5 μm. **(C)** Z-slice images of **(B)** showing the distribution of subapical actin patches, actin cables, and actin at the Spk in an active hypha. **(D)** 3D image obtained from photographs in **(B)** and **(C)** showing the spatial structure of the actin cytoskeleton in the hyphal tip. The corresponding movie is provided as S2 Movie. **(E)** Time-lapse images (10 time points) showing the dynamic transport of patches along the actin bundle. The red arrows indicate the actin patches. The corresponding movie is provided as S3 Movie. Bar = 2 μm. **(F)** A proposed model showing the bowl-shaped organization of the actin cytoskeleton in the hyphal tip.

As fimbrin is a putative actin-binding protein, we labeled the actin cytoskeletons of the WT and *Mofim1* hyphae with the widely used Lifeact-GFP peptide probe [21, 33, 34]. Given that the organization of actin in developing *M*. *oryzae* hyphae is not well understood, we examined the dynamic distribution of actin filaments during *M*. *oryzae* development using a super-resolution live-cell imaging system. In growing WT vegetative hyphae, a population of actin filaments was continuously generated from the Spk area; the generation of these filaments was tightly linked with hyphal growth (Fig 2A and S1 Movie). In addition to the actin at the Spk and in actin cables, we also observed actin accumulation in patches at the subapical collar region (Fig 2B and 2C). Images from Z-slice projections show that the subapical actin patches mainly clustered close to the cell membrane (Fig 2C, slice Z-1 and Z-2). The actin cables that originated from the Spk formed long, branched actin bundles radiating into the subapical region; these bundles appeared as a bowl-shaped structure at the hyphal head (Fig 2D and S2 Movie). Furthermore, we noticed that some patches travelled dynamically along the actin cables (Fig 2E and S3 Movie). We developed a schematic representation of actin organization and remodeling associated with hyphal growth based on our observations (Fig 2F). These results uncover a unique actin organization pattern underlying elongation that occurs via tip or diffuse growth.

**Fig 3:**
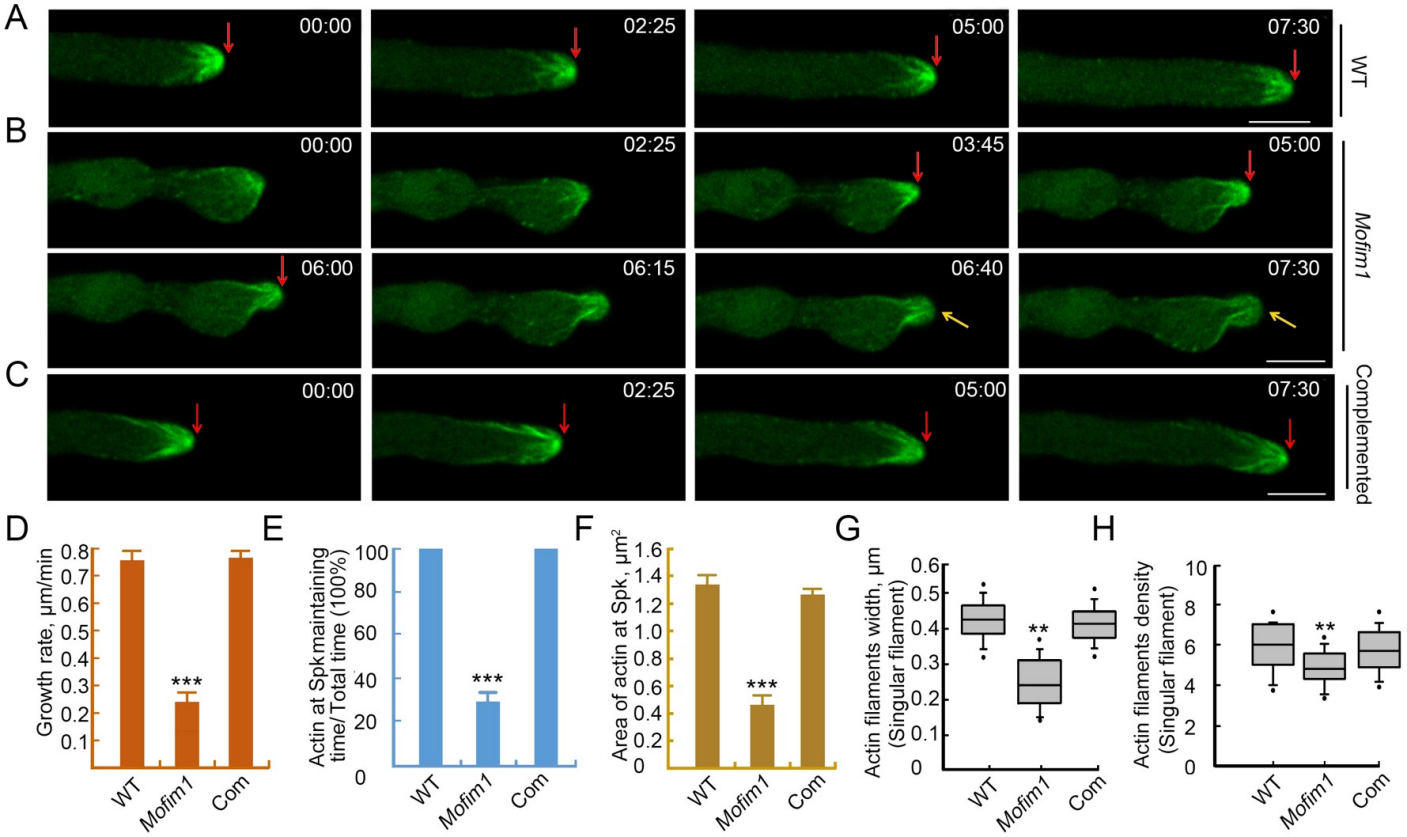
Defects of the *Mofim1* mutant impaired in actin organization in the hyphal tip during polar tip growth. **(A–C)** Time-lapse images showing actin dynamics in growing WT **(A)**, *Mofim1* mutant **(B)**, and the complemented **(C)** hyphae. The corresponding movie is provided as S4 Movie. The red arrows indicate the presence of actin at the Spk during polar growth. The yellow arrows in **(B)** indicate the absence of this structure. The numbers at the top right corner indicate the timestamps (min:s). Bar = 2 μm. **(D–G)** Quantitative analysis of the growth rate **(D)**, maintaining time of actin at Spk **(E)**, area of actin at Spk **(F)**, width of actin cables **(G)**, and actin density **(H)** in WT, *Mofim1* and the complemented strain (100 hyphae each of the WT, *Mofim1*, and the complemented strain in three experimental repeats). Data are means ± SE (*n* = 100); asterisks indicate statistically significant differences, as determined by Student’s *t*-test (***, *P* < 0.001).

The hyphae of WT and complemented lines produced a population of dense actin filaments converging in the Spk during growth (Fig 3A and 3C, S4A and S4C Movie). The mean growth rate was ~0.79 and 0.80 μm/min for the WT and the complemented hyphae, respectively (Fig 3D, S5A and S5C Movie). However, in *Mofim1* hyphae, we observed intermittent appearance of actin at the Spk and the average growth rate was only ~0.25 μm/min (Fig 3D, S5A and S5B Movie). When the mutant hyphae were growing, the hyphal tips gradually became pointed and, at the same time, actin was observed at the Spk and Spk-converging actin filaments formed (from 3:45 [min:s] to 6:00 in Fig 3B). The growth rate in this time period was ~0.68 μm/min. Subsequently, the actin at the Spk gradually disappeared and the actin filaments became loosely distributed in the apical regions of the hyphae. Finally, the growth rate decreased to ~0.1 μm/min and the hyphal tips began to expand (from 6:00–7:30 in Fig 3B and S4B Movie). Quantitative analysis showed that actin at the Spk was maintained in mutant hyphae for ~30% of the time that it was maintained in the WT and the complemented hyphae during growth (Fig 3E). The area of the actin at the Spk, and the width and number of the actin filaments were significantly reduced in the *Mofim1* mutant (Fig 3F–3H).

We performed fluorescence recovery after photo bleaching (FRAP) analysis to assess the turnover of the actin cytoskeleton in the WT versus the *Mofim1* mutant. Actin fluorescence recovered to a higher level in WT cells compared to *Mofim1* cells, indicating that the dynamic state of actin was reduced in the mutant cells (S4 Fig and S6 Movie). Together, these results indicate that MoFim1 plays important roles in organizing the actin cytoskeleton in the hyphal tip and is therefore crucial for hyphal morphogenesis and tip growth.

### MoFim1 colocalizes with the actin cytoskeleton

**Fig 4:**
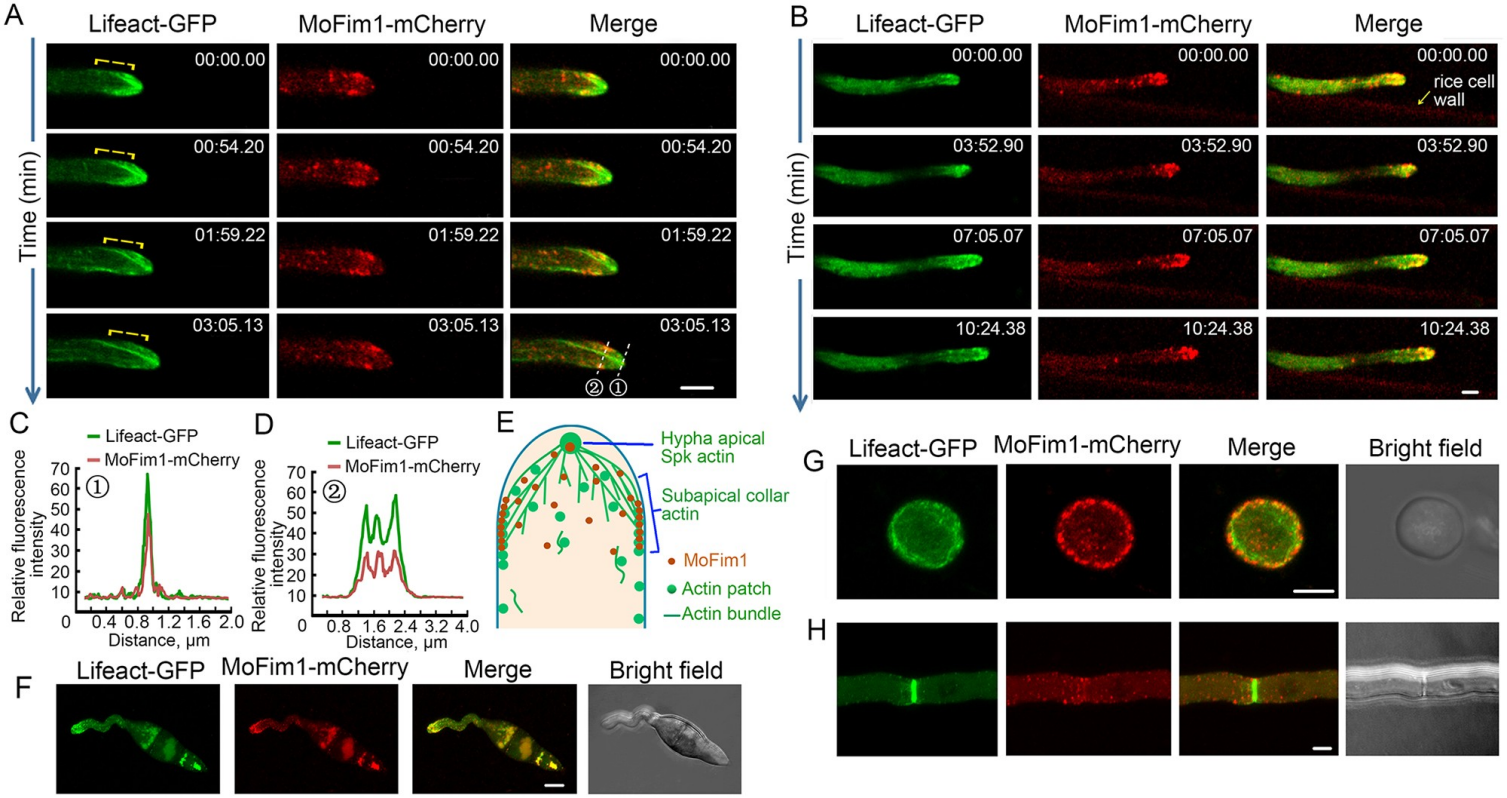
Localization of MoFim1 in *M*. *oryzae*. **(A and B)** Expression of p*MoFim1*-*MoFim1-mCherry* in Lifeact-GFP-labeled *Mofim1* mutant vegetative **(A)** and invasive **(B)** hyphae. The numbers at the top right corner indicate the timestamps (min:s). The yellow brackets indicate the subapical collar region. Bars = 2 μm. **(C and D)** Line-scan analysis of the localization of MoFim1 related to the actin cytoskeleton. **(E)** Localization of MoFim1 in the hyphal tip. **(F–H)** Localization of MoFim1 in the *M*. *oryzae* germ tube **(F)**, appressorium **(G)**, and septum **(H)**. Bars = 5 μm.

To investigate how MoFim1 regulates the dynamics of the actin cytoskeleton in the hyphal tip, we observed the localization of MoFim1-mCherry in *M*. *oryzae*. *MoFim1- mCherry* driven by its native promoter and Lifeact-GFP were coexpressed in the *Mofim1* mutant, and the colocalization of GFP and mCherry was observed by super-resolution live-cell imaging microscopy. Time-lapse imaging of vegetative hyphae revealed that MoFim1 formed dense patches in the cytoplasm or the actin collar at the subapical region. MoFim1-mCherry also colocalized with the actin at the Spk in rapidly growing hyphae. Some MoFim1-mCherry patches in the cytoplasm localized to actin cables (Fig 4A and S7 Movie). MoFim1 exhibited a similar distribution in invasive hyphae: MoFim1 accumulated at the cell tip, with strong signals from the Lifeact-GFP-labeled actin cytoskeleton (Fig 4B, S5 Fig and S8 Movie). Line-scan analysis further supported the colocalization of MoFim1-mCherry and the actin cytoskeleton at the Spk and subapical collar (Fig 4C and 4D).

Based on our observations, we developed a model of the localization pattern of MoFim1 in hyphae (Fig 4E). MoFim1-mCherry patches were also visible at the tip of the germ tube, the actin at the periphery of the cell in the appressorium, and the septum (Fig 4F–4H). These results indicate that MoFim1 closely associates with the actin cytoskeleton during polarized hyphal growth.

### MoFim1 binds to and bundles actin filaments

As fimbrin is a major regulator of actin organization [26, 28], we investigated the biochemical basis for the function of MoFim1. After producing His-tagged recombinant MoFim1 in *Escherichia coli*, we performed a high-speed co-sedimentation assay to assess the ability of MoFim1 to bind to actin filaments. As shown in S6A Fig, MoFim1 bound to and coprecipitated with actin filaments. When we incubated F-actin (4 μM) with MoFim1, the amount of MoFim1 in the pellet increased in proportion to the MoFim1 concentration (0–8 μM).

Since fimbrin family members generally bundle actin filaments, we performed a low-speed cosedimentation assay to determine whether MoFim1 also possesses this property. As shown in S6B Fig, the amount of F-actin in the supernatant decreased in the presence of increasing levels of MoFim1, indicating that more F-actin bundles formed and precipitated in the pellets.

**Fig 5:**
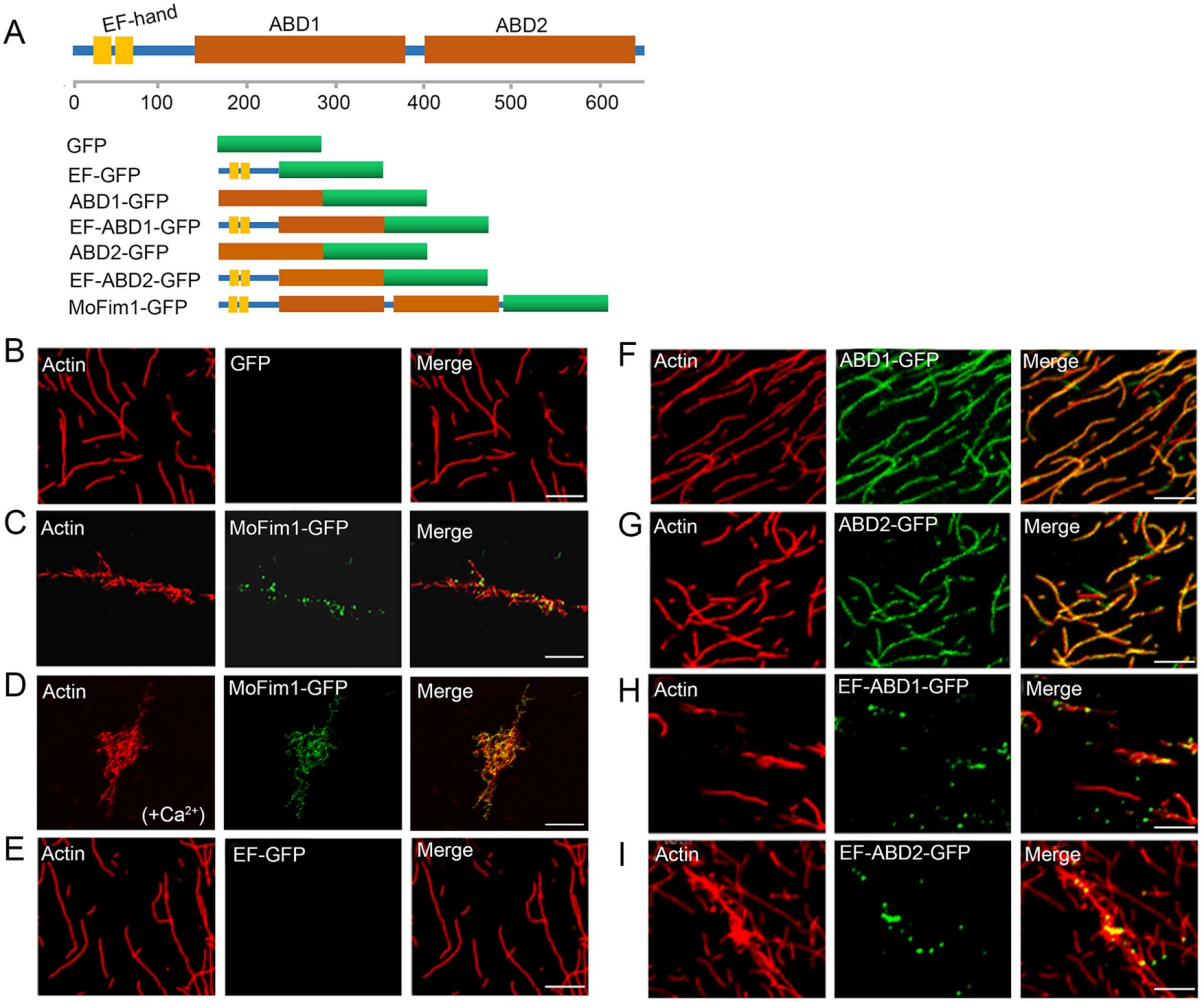
Biochemical analysis of MoFim1. **(A)** Schematic diagram showing the protein domains of MoFim1 and GFP-fused MoFim1 and truncated proteins. **(B–I)** Actin binding/bundling analysis of MoFim1 and its various domains by fluorescence observation. Polymerized F-actin (1 μM) incubated with various proteins (1 μM) was used for analysis. Alex561-phalloidin-labeled F-actin alone **(B)**; F-actin in the presence of MoFim1-GFP **(C)**; F-actin in the presence of MoFim1-GFP and Ca^2+^ (1 μM) **(D)**; F-actin in the presence of EF-GFP **(E)**; ABD1-GFP **(F)**; ABD2-GFP **(G)**; EF-ABD1-GFP **(H)** and EF-ABD2-GFP **(I)**. Bars = 1 μm.

MoFim1 is composed of an N-terminal EF-hand (EF) motif and two actin-binding domains (ABD1 and ABD2) in tandem (Fig 5A). To elucidate the actin binding and bundling mechanism of MoFim1, we used an *in vitro* system to produce GFP-fused full-length and truncated versions of MoFim1, as shown in Fig 5A. We incubated these proteins with polymerized F-actin to evaluate their F-actin binding/bundling activities. Single-molecule imaging of phalloidin-stained F-actin revealed fine actin filaments when incubated with the GFP control (Fig 5B). However, when incubated with MoFim1-GFP, thick actin bundles appeared, and punctate MoFim1-GFP signals were visible on the actin filaments (Fig 5C). These results indicate that MoFim1 binds to and cross-links F-actin to form actin bundles. Large actin aggregates formed in the presence of Ca^2+^, implying that the actin bundling activity is accelerated by Ca^2+^ (Fig 5D).

We also analyzed the biochemical properties of the MoFim1 domains shown in Fig 5A. The ABD1 and ABD2 domains, but not the EF domain, bound to F-actin. All three domains lacked F-actin bundling activity (Fig 5E–5G). Moreover, when the EF domain was fused to ABD1 and ABD2, both EF-ABD1 and EF-ABD2 bound to and bundled actin filaments (Fig 5H and 5I). These results suggest that the EF domain facilitates the actin binding/bundling activities of MoFim1 via the formation of homodimers. This notion was further confirmed by a yeast two-hybrid assay, as shown in S7 Fig.

**Fig 6:**
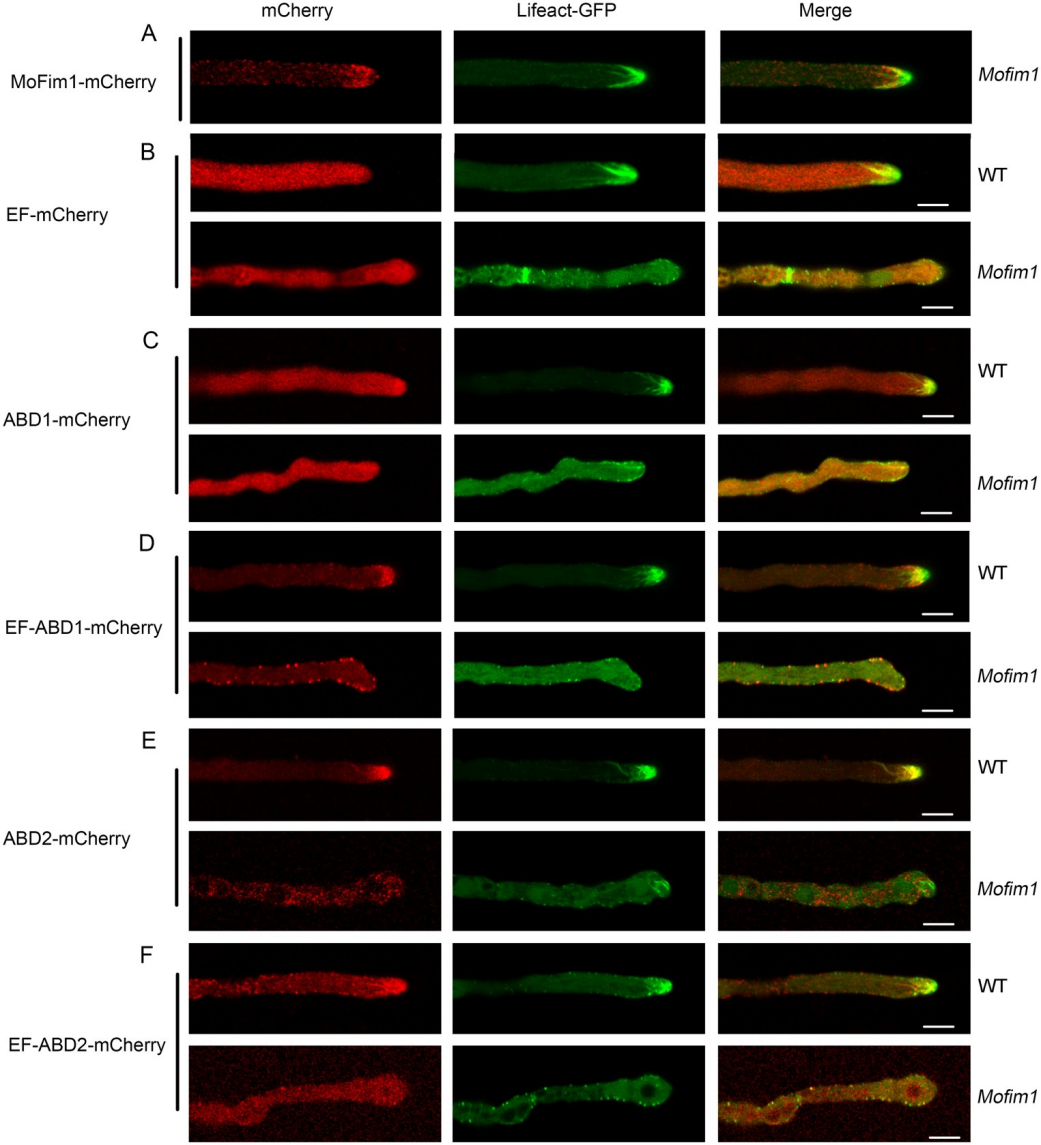
*In vivo* analysis of the characteristics of truncated MoFim1 proteins. MoFim1 and its domains fused with mCherry were expressed under the control of the native promoter of *MoFim1*. Expression of MoFim1-mCherry in Lifeact-GFP-labeled *Mofim1* mutant hyphae **(A)**. Expression of the truncated version of MoFim1 including EF **(B)**, ABD1 **(C)**, EF-ABD1 **(D)**, ABD2 **(E)**, EF-ABD2 **(F)** fused with mCherry in Lifeact-GFP-labeled WT and *Mofim1* mutant hyphae, respectively. Bars = 5 μm.

In addition to these *in vitro* experiments, we performed genetic complementation tests using genomic constructs with various domain truncations to unravel the molecular and cellular basis of MoFim1 activity. None of the truncated versions of *MoFim1*, including *EF*, *ABD1*, *ABD2*, *EF-ABD1*, and *EF-ABD2* driven by the *MoFim1* native promoter, rescued the *Mofim1* mutant phenotype (S8 Fig). In contrast to the distribution of the intact MoFim1 in the Spk, actin cables, and the subapical collar (Fig 6A), EF-mCherry was distributed uniformly inside WT and *Mofim1* hyphae (Fig 6B). ABD1-mCherry accumulated at the hyphal apex and colocalized with actin at the Spk (Fig 6C). When EF was fused to ABD1, brighter mCherry signals were observed at the Spk. However, this fusion protein was not obviously colocalized with F-actin in the subapical region (Fig 6D). By contrast, ABD2-mCherry labeled both actin at the Spk and F-actin at the subapical region of the *M*. *oryzae* hyphae (Fig 6E), and the EF motif promoted its actin labeling efficiency (Fig 6F).

### MoFim1 is involved in actin cytoskeleton-mediated endocytosis

**Fig 7:**
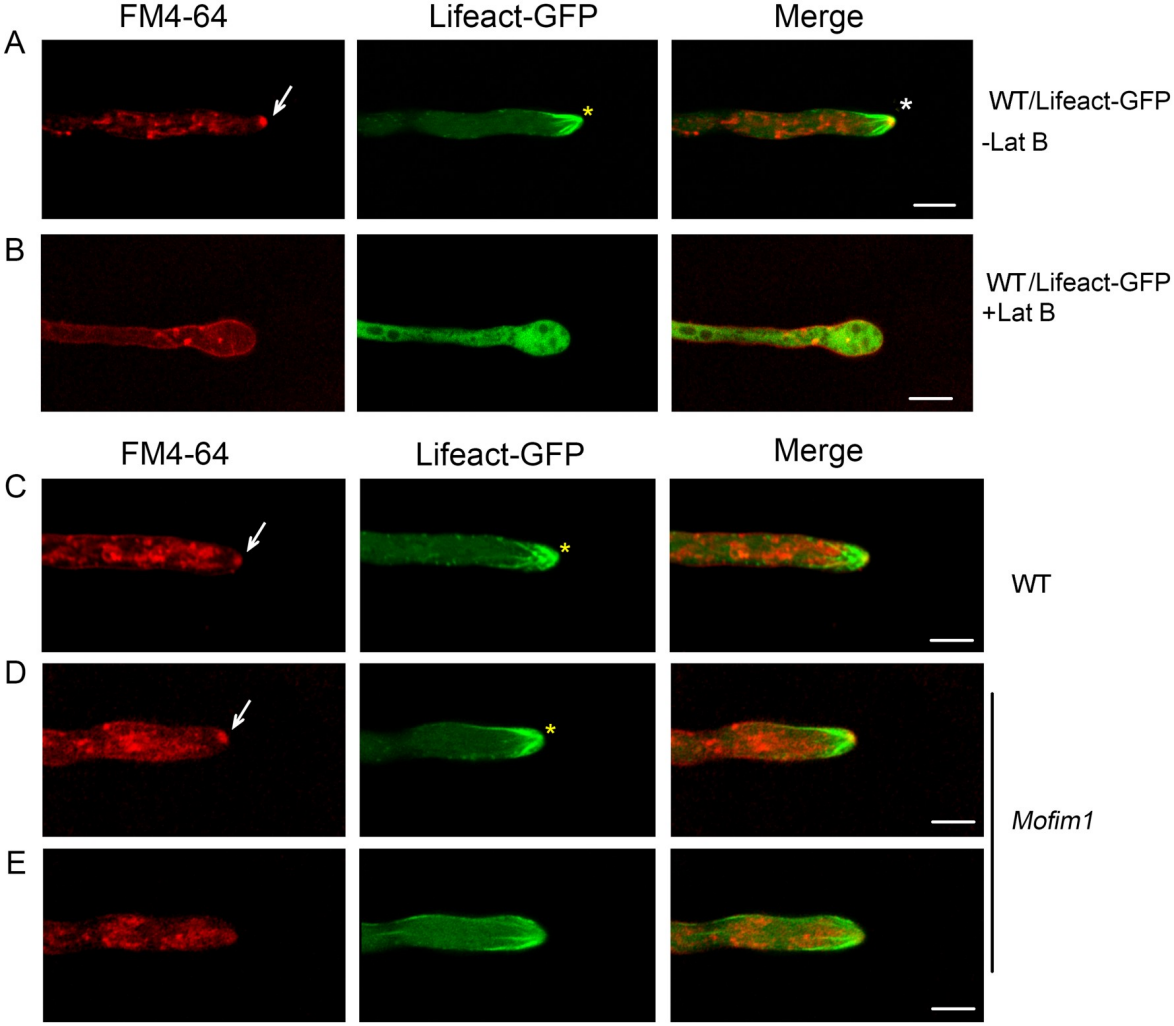
Defects in actin cytoskeleton-mediated endocytosis in *Mofim1*. **(A)** Colocalization of the actin cytoskeleton and FM4-64-labeled Spk. The arrowhead indicates FM4-64-labeled Spk. The asterisk indicates the fluorescence merge of FM4-64 and actin at the Spk. Bar = 5 μm. **(B)** Colocalization of Lifeact-GFP and FM4-64 in hyphae pretreated with Lat B for 30 min. Bar = 5 μm. **(C–E)** Representative images showing FM4-64 stained hyphae of WT **(C)** or the *Mofim1* mutant with **(D)** or without a Spk **(E)**. The arrowheads indicate FM4-64-labeled Spk and the yellow asterisks indicate actin at the Spk. Bars = 5 μm.

We then investigated the physiological functions of MoFim1. The actin collar plays a role in endocytosis [7] and some MoFim1 localized to the actin collar, indicating that MoFim1 functions in endocytosis (Fig 4). To verify this notion, we stained the hyphae with FM4-64, a widely used marker of endocytosis. Approximately 5 min after incubation with FM4-64, the red signals were endocytosed in the cytoplasm of the hyphae and delivered to the hyphal tip, where they then colocalized with the actin at the Spk (Fig 7A).

To investigate the roles of the actin cytoskeleton in endocytosis, we treated the hyphae with the actin-depolymerizing drug latrunculin B (Lat B). The actin cytoskeleton, including the actin collar, actin cables, and Spk, were destroyed upon Lat B treatment (Fig 7B). These hyphae failed to undergo polar growth; instead, the hyphal tip began to inflate. Strong red signals from FM4-64 accumulated at the hyphae cell membrane, indicating that the internalization of this endocytotic marker had been delayed. Moreover, the endocytosed FM4-64 was not transported to the Spk region (Fig 7B). We next stained *Mofim1* hyphae with FM4-64 and found that FM4-64 could be transported to the Spk when actin was accumulating at the Spk (Fig 7D). Actin appeared only intermittently at the Spk in the *Mofim1* mutant (Fig 3B); when actin at the Spk depolymerized, accumulation of FM4-64 at the Spk was subsequently attenuated (Fig 7E). Together, these results indicate that MoFim1 is required for actin cytoskeleton-mediated endocytosis.

### MoFim1 organizes the actin cytoskeleton in the hyphal tip for septin-dependent assembly of the exocyst

The failure of Lat B-treated WT and *Mofim1* hyphae to accumulate of FM4-64 at the Spk (Fig 7B and 7E) indicated that the delivery of secretory vesicles is dependent on the actin cytoskeleton in the hyphal tip. The septin-dependent exocyst in *M*. *oryzae* is located near the Spk to facilitate the delivery of secretory vesicles to the plasma membrane during polarized exocytosis [12]. Therefore, we examined whether protein secretion would be affected in *Mofim1* grown under axenic culture conditions compared to the WT. The *Mofim1* mutant exhibited a >50% reduction in protein secretion compared to the WT (S9 Fig), indicating that exocytosis was severely affected in this mutant. Mass spectrometry (MS) analysis of these secreted proteins identified more than one hundred proteins, most of which were down-regulated in the *Mofim1* mutant. Of these, the amount of the effector AVR-Pia secreted by the *Mofim1* mutant was 0.43-fold that secreted by the WT, indicating that MoFim1-mediated actin assembly is required for secretion of effectors (S1 Table).

**Fig 8:**
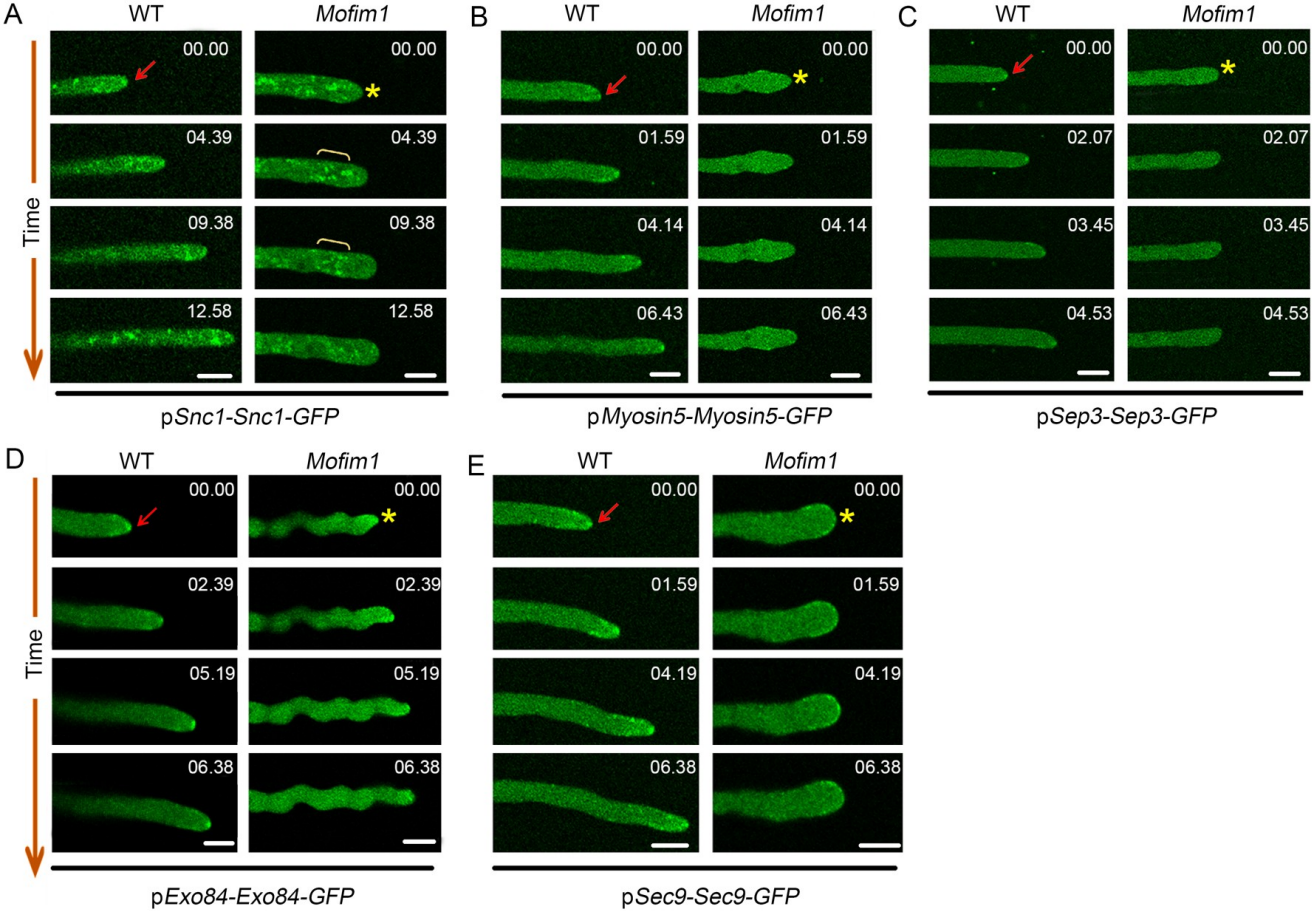
Compromised septin-dependent exocyst in *Mofim1*. The indicated genes encoding the v-SNARE protein Snc1 **(A)**, actin motor protein Myosin5 **(B)**, actin scaffold protein Sep3 **(C)**, exocyst component Exo84 **(D)**, and t-SNARE protein Sec9 **(E)** were fused with GFP and driven by their native promoters. The constructs were transformed into WT and *Mofim1*. The red arrowheads indicate the apical accumulation of the indicated proteins in WT hyphae and the yellow asterisks indicate those in *Mofim1*. These images are related to S9–S12 Movies. The numbers at the top right corner indicate the timestamps (min:s). Bars = 5 μm.

To investigate which step was impaired during polarized exocytosis, we introduced Snc1-GFP (Snc1, a putative vesicle-bound v-SNARE protein) into the WT and *Mofim1* mutant backgrounds. In the WT, Snc1-GFP-labeled vesicles were actively transported to the growing hyphal tip (Fig 8A, and S9 Movie). However, in the *Mofim1* mutant, the vesicles moved to the subapical region of the hyphae but failed to move forward to the hyphal tip. The vesicles usually formed aggregates at this site (Fig 8A and S9 Movie). These results indicate that polarized vesicle exocytosis was impaired in *Mofim1*.

Next, we introduced Myosin5-GFP fusion protein into the WT and *Mofim1* mutant backgrounds. Class V myosin is an actin-based motor responsible for the short-distance transport of vesicles to the Spk and their delivery to specific regions of the hyphal apex [36–38]. Myosin5-GFP appeared as a bright spot at the growing tips of WT hyphae. By contrast, in *Mofim1*, no signal was detected at the hyphal tip. Instead, some small spots appeared at the cell membrane (Fig 8B and S10 Movie). These findings indicate that the actin motor protein Myosin5 is distributed in a disorderly fashion in *Mofim1*. We also used septin3-GFP to label the Spk. In agreement with the results from FM4-64 staining, no septin3 accumulated in the Spk area in *Mofim1* (Fig 8C and S11 Movie). Finally, we observed the expression of the exocyst component Exo84, and the membrane-bound t-SNARE Sec9 in the WT and *Mofim1*. Both proteins localized to puncta at the cell apex in WT. In the mutant, the distribution pattern of Exo84 proteins was similar to that in the WT. The membrane-bound t-SNARE protein Sec9 was detected at the cell membrane, although its distribution pattern was diffuse (Fig 8D and 8E, S12 Movie). These results indicate that without MoFim1, the activity of the septin-dependent exocyst is compromised prior to tethering secretory vesicles to the plasma membrane.

### MoFim1 is required for invasive hyphal growth and expansion in rice cells

**Fig 9:**
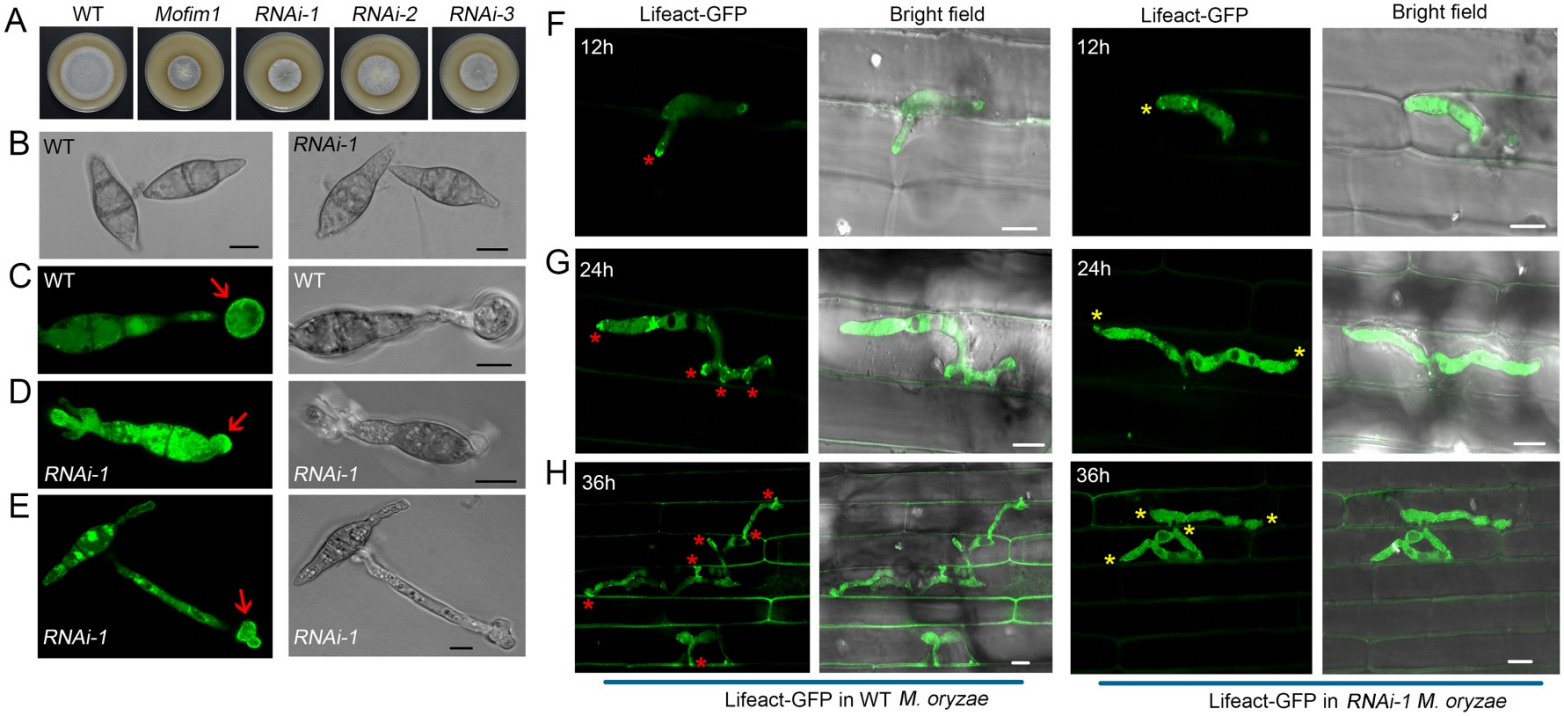
MoFim1 functions in actin cytoskeleton organization in invasive hyphae, which is required for hyphal expansion in rice cells. **(A)** Images of 7-d-old cultures including WT, *Mofim1*, and three *MoFim1* silencing strains. **(B)** Images of conidia in WT and RNAi-1 strains. Bars = 10 μm. **(C–E)** Morphology and actin organization in WT **(C)** and RNAi-1 strains **(D and E)**. The red arrows indicate the induced appressorium in the WT and the RNAi-1 strains labeled with Lifeact-GFP. Bars = 5 μm. **(F–H)** Actin organization at different time points in invasive hyphae of WT (left panels) and RNAi-1 (right panels). The red asterisks indicate actin accumulation at the hyphal tip or the narrow site in which the hyphae penetrate into neighboring cells. Yellow asterisks indicate the area described above in RNAi-1. Bars = 5 μm.

The *Mofim1* mutant exhibited disrupted conidiation, preventing us from investigating its intracellular behavior in plant cells. Therefore, we generated a *MoFim1* RNAi line to knock down *MoFim1* expression. Three DNA fragments (~200 bp from the 5′ terminus, the middle region, and the 3′ terminus of the *MoFim1* open reading frame) were used for RNA hairpin construction (named RNAi-1, RNAi-2, and RNAi-3, respectively). We transformed these RNAi constructs into WT *M*. *oryzae*. The RNAi construct from the 5′ terminus (RNAi-1) exhibited good efficiency for silencing *MoFim1*. The growth rate of the RNAi-1 line was comparable to that of the *Mofim1* knockout mutant, in contrast to RNAi-2 and RNAi-3 (Fig 9A). Although conidiation was dramatically reduced in RNAi-1 (~20% of the WT rate), the RNAi-1 *M*. *oryzae* spores did not exhibit great changes in morphology compared to the WT (Fig 9B). When we examined appressorium formation on hydrophobic glass surfaces, after 8 h of induction, a circular appressorium labeled with Lifeact-GFP appeared in the WT (Fig 9C). However, in RNAi-1, the appressoria were usually deformed (Fig 9D and 9E).

The RNAi-1 spores usually developed long hyphae outside of the rice epidermal sheath cells and only a small portion could penetrate (S10 Fig). In invasive WT hyphae, an actin cytoskeleton was usually present in the hyphal tip or at the narrow site in which the hyphae penetrated into the neighboring cell (left panels of Fig 9F–9H). By contrast, invasive RNAi-1 hyphae rarely exhibited this type of actin organization (right panels of Fig 9F–9H). Most invasive hyphae of RNAi-1 were confined to the first penetrated cell (102 cells were examined). By contrast, 80% of WT invasive hyphae (105 cells were examined) penetrated into the other cells, but only ~12% of those in RNAi-1 (118 cells were examined) expanded to the neighboring cells at 36 h after infection (Fig 9H). These results indicate that MoFim1 is required for both the initial plant cell penetration and the expansion of hyphae from one plant cell to another.

### Host-induced gene silencing of *MoFim1* improves rice blast resistance

**Fig 10:**
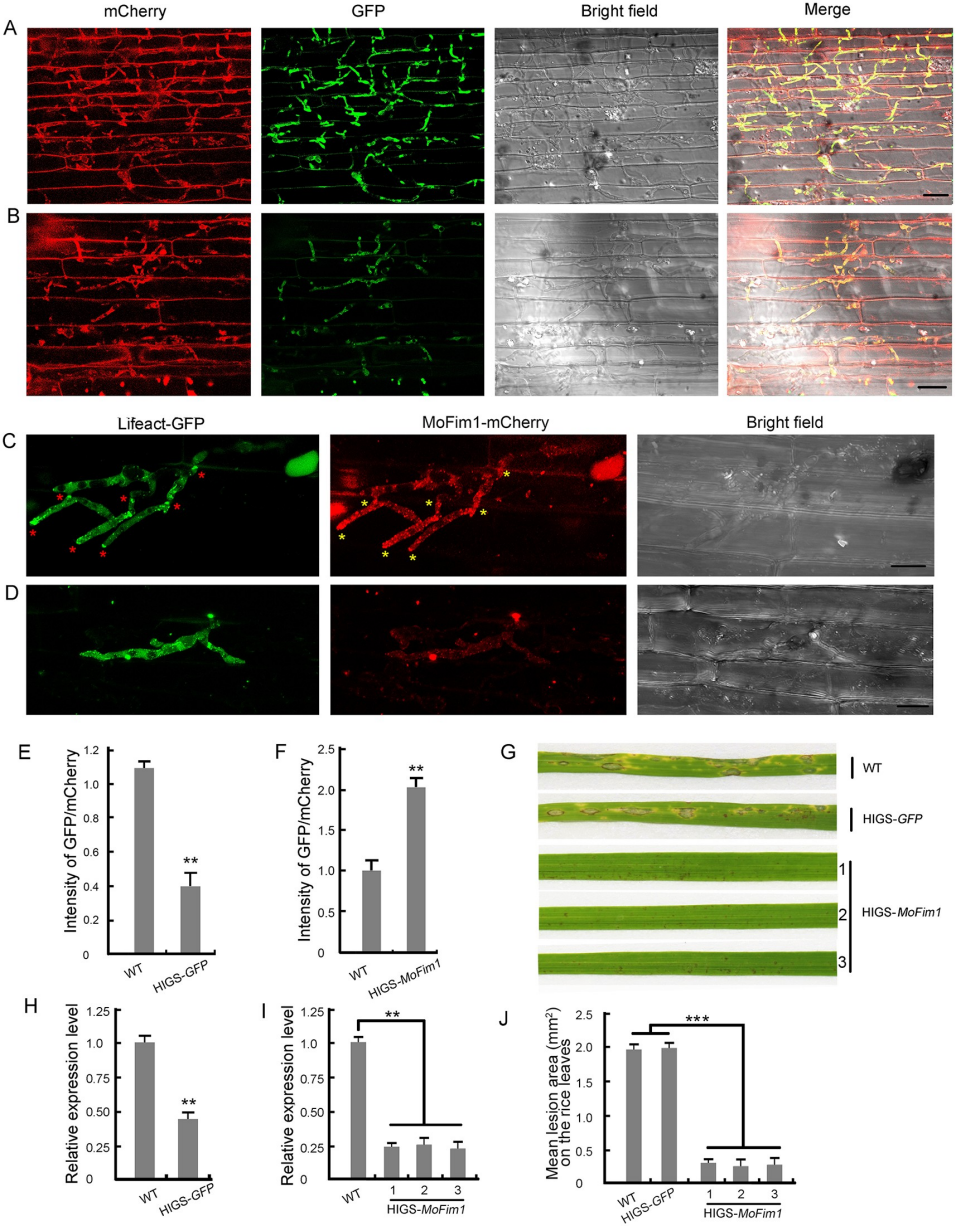
*MoFim1*-RNAi transgenic plants exhibit resistance to rice blast disease. **(A and B)** Fluorescent images of WT **(A)** and HIGS-*GFP* transgenic **(B)** rice sheath cells infected by WT *M*. *oryzae* labeled with GFP/mCherry for 72 h. Bars = 20 μm. **(C and D)** Fluorescent images of WT **(C)** and HIGS-*MoFim1* transgenic **(D)** rice sheath cells infected by *Mofim1* labeled with Lifeact-GFP/MoFim1-mCherry for 72 h. The red asterisks indicate actin accumulation at the hyphal tip or the narrow site in which the hyphae penetrate into neighboring cells. Yellow asterisks indicate the MoFim1-mCherry accumulation at the area described above. Bars = 20 μm. **(E–F)** Fluorescence intensity of GFP/mCherry from invasive hyphae in WT and HIGS-*GFP* transgenic rice **(E)**, and WT and HIGS-*MoFim1* transgenic rice **(F)**. Data are means ± SE, *n* ≥ 50 cells. Asterisks indicate statistically significant differences, as determined by Student’s *t*-test (**, *P* < 0.01); Similar results were obtained in three independent biological repeats. **(G)** Pathogenicity assay of WT, HIGS-*GFP*, and HIGS-*MoFim1* rice plants. Conidial suspensions of WT strains were sprayed onto 2-week-old rice seedlings (*O*. *sativa* ssp. *japonica* cv. Nipponbare). Diseased leaves were photographed after 5 d of inoculation. **(H and I)** qRT-PCR analysis of *GFP* and *MoFim1* expression in WT versus HIGS-*GFP* **(H)** or HIGS-*MoFim1* **(I)** transgenic plants. Error bars represent SD from three independent experiments (*n* = 3). Asterisks indicate statistically significant differences, as determined by Student’s *t*-test (**, *P* < 0.01). **(J)** Quantification of the lesion area on the leaves of WT, *HIGS-GFP*, and HIGS-*MoFim1* T_2_ transgenic rice plants. Asterisks indicate statistically significant differences, as determined by Student’s *t*-test (***, *P* < 0.001, *n* ≥ 20).

Host-induced gene silencing (HIGS) is a powerful strategy for developing transgenic rice cultivars to control fungal diseases and is a useful tool for investigating gene function in pathogens [39, 40]. To test HIGS with *MoFim1*, we generated transgenic rice lines (*O*. *sativa* ssp. *japonica* cv. Nipponbare, susceptible to *M*. *oryzae* strain Y34) overexpressing artificial small interfering RNAs (siRNAs) produced from the same DNA fragment used for construction of RNAi-1. We also generated transgenic lines for HIGS of *GFP* as a parallel negative control. Southern blot analysis showed that the three selected T_2_ HIGS-*MoFim1* transgenic lines were all single-copy insertions (S11 Fig). Specifically, WT *M*. *oryzae* spores subjected to dual fluorescence labeling with GFP and mCherry were used in the rice leaf sheath penetration assays. After 72 h of infection, GFP/mCherry-labeled *M*. *oryzae* successfully penetrated into WT rice sheath cells. Strong GFP and mCherry signals from *M*. *oryzae* were observed in the plant cells (Fig 10A). We also incubated GFP/mCherry-labeled *M*. *oryzae* spores with HIGS-*GFP* transgenic rice sheath cells. While fluorescent mCherry signals were not obviously altered in these cells, the intensity of GFP signals was significantly reduced in *M*. *oryzae* that had penetrated into plant cells (Fig 10B and 10E). qRT-PCR analysis confirmed the silencing of *GFP* expression in HIGS-*GFP* transgenic rice cells (Fig 10H), verifying that the HIGS system functioned efficiently.

We used Lifeact-GFP/MoFim1-mCherry double-labeled *M*. *oryzae* to infect sheath cells from WT and HIGS-*MoFim1* transgenic rice plants. In WT sheath cells, Lifeact-GFP-labeled actin accumulated in some invasive hyphal tips or at the narrow sites in which the hyphae penetrated into neighboring cells (as observed in Fig 9). MoFim1-mCherry colocalized with these actin aggregates at these sites (Fig 10C). However, in HIGS-*MoFim1* transgenic rice plants, along with the significantly reduced fluorescence intensity of MoFim1-mCherry, a Lifeact-GFP labeled actin cytoskeleton was not usually detected at the hyphal tip or the narrow hyphal penetration site (Fig 10D and 10F). The reduced expression of *MoFim1* was further verified by qRT-PCR (Fig 10I). In addition, most invasive hyphae were confined to the first penetrated cell, which is reminiscent of the infection defects in RNAi-1 (Fig 9). Finally, when we sprayed WT *M*. *oryzae* spore suspensions onto WT and HIGS-*MoFim1* transgenic plants, after 5 d of inoculation, the HIGS-*MoFim1* plants produced significantly smaller lesions than the controls (Fig 10G and 10J). These findings indicate that HIGS of *MoFim1* improves rice resistance against *M*. *oryzae*.

## Discussion

### *M*. *oryzae* forms a unique actin structure that facilitates hyphal growth and infection

The growth of hyphal cells of filamentous fungi occurs at the tip via a process analogous to pollen tube growth and root hair elongation [41]. This process, referred to as tip growth, requires precise regulation of the actin cytoskeleton. Characterizing the various actin structures in these cell types is currently an active area of research. For example, a population of apical actin filaments has been observed at the growing apex of the Arabidopsis pollen tube. These actin filaments, which originate from the apical membrane, form a specialized structure for vesicle transport [34]. Many interconnected, thick actin bundles are present in the shaft of the root hair. However, at the apex of the growing root hair, the actin organization markedly changes, as actin bundles become progressively thinner and a dense array of filamentous actin is present [42]. Yu et al. (2019) recently investigated the organizing principles and dynamic behavior of the cytoskeleton in cotton (*Gossypium hirsutum*) fibers, which elongate via a tip-biased but diffuse growth mode. The elongating cotton fibers assemble a cortical filamentous actin network that extends along the cell axis, ultimately forming actin strands with closed loops in the tapered fiber tip [43]. These findings provide important insights into the cellular mechanisms of tip growth during cell elongation.

In the filamentous fungi, dynamic assembly of the actin cytoskeleton forms diverse actin structures in development and pathogenesis. Considerable effort, using actin-binding protein or peptide (Lifeact) labeling, has shown that the actin cytoskeleton assembles as patches, cables, and rings in filamentous fungi. In *Neurospora crassa*, the actin cytoskeleton forms a subapical collar of actin patches and a concentration of actin within the core of the Spk [44–47]. In actively growing *Aspergillus nidulans* hyphae, the actin assembles as patches, which are highly mobile throughout the hyphae and are concentrated near hyphal apices [30]. A subapical actin web was observed at several microns behind the growth site [48]. In *M*. *oryzae*, the actin cytoskeleton is thought to play crucial roles in development and pathogenesis [19, 20, 49]. A toroidal F-actin ring assembles in the appressorium and is required for the protrusion of a rigid penetration peg to breach the leaf surface [21]. These findings lay the foundation for investigating the roles of the actin cytoskeleton in *M*. *oryzae* infection. The *M*. *oryzae* MAPK Pmk1 was recently found to function upstream of the actin cytoskeleton, regulating its organization during the infection of adjacent plant cells [22]. However, despite the importance of the actin cytoskeleton, our understanding of how the actin cytoskeleton functions is limited, and we lack a unified view of the organization and dynamics of actin filaments, especially during hyphae polar growth and expansion in plant cells. In this study, through super-resolution live-cell imaging, we found that *M*. *oryzae* (like the fungus *Neurospora crassa*) produced a subapical collar of actin patches and intense actin at the Spk (Fig 2). In addition, we detected a dense assembly of actin filaments that were continuously generated from the Spk and extended (by ~5 μm) into the subapical area of the hyphal tip (Fig 2B, S1 Movie). These actin filaments between the Spk and subapical actin collar have not been clearly observed in other fungi such as *Neurospora crassa*, *Aspergillus nidulans*, or *Colletotrichum graminicola* [30, 44–48, 50]. We further noticed that these F-actin filaments radiated from the Spk and were connected to the cell membrane, forming bowl-shaped structures capping the hyphal tips (Fig 2B–2D). Since the delivery of endocytosed vesicles to the Spk area is dependent on the actin cytoskeleton (Figs 7 and 8A), it appears that the endocytosed vesicles, as well as the polarisome, are delivered to the subapical region and transported along F-actin cables to the Spk. Thus, based on our observations, we identified a unique organization pattern of actin at the hyphal tip unlike those observed in plant pollen tubes, root hairs, or cotton fibers.

A previous study revealed that F-actin also assembled in invasive hyphae and the actin cables might play an essential role in plant infection [50]. Until now, our knowledge of the dynamic organization of actin in invasive hyphae has remained limited. We determined that the actin cytoskeleton also accumulates in some invasive hyphal tips (but not in bulbous invasive hyphae) (Figs 4B, 9F–9H and 10C), indicating that these cells undergo polarized growth. Although obvious accumulation of actin at the apex of the invasive hyphae was detected (Fig 9F–9H), fine F-actin filaments were not frequently observed in these hyphae, like those in vegetative hyphal tips (Fig 4A and 4B). This perhaps was due to the stress environment in plant cells, which affects F-actin formation.

In addition to the hyphal tip, the actin cytoskeleton accumulated at the narrow sites in which hyphae penetrate into the neighboring cells (Fig 9H). In line with our results, the actin cytoskeleton scaffold protein Septin5 assembles at the cell wall contact points, highlighting the organization of the actin cytoskeleton at this region [22]. Mutations of the MAPK Pmk1 affect the location of Septin5 at this site, thus impairing cell-to-cell invasion by *M*. *oryzae* [22]. Based on these and the current observations, we speculate that when hyphae invade the adjacent plant cells, the actin in the hyphal tip rapidly becomes organized to build polarity to point towards the plasmodesmata and to cause the hyphae to become narrower, thereby helping them cross into neighboring cells.

### MoFim1 organizes the actin cytoskeleton for hyphal tip growth and polarized exocytosis in *M*. *oryzae*

Fimbrins are well-characterized actin bundling proteins that function through their two conserved ABDs [51]. *In vitro* analysis of the biochemical properties of MoFim1 revealed that MoFim1 directly binds to and bundles actin filaments (Fig 5C, and S6 Fig), pointing to the conserved activities of this protein in actin assembly. Vertebrate fimbrins also contain an N-terminal calcium-binding domain consisting of two EF-hand-like motifs that precede two ABDs, but fimbrins in yeast and plants lack this motif [25]. The functional significance of these EF-hand-like motifs is unclear, although they might play a role in calcium binding [52]. In this study, *in vivo* and *in vitro* experiments showed that the EF motif alone could not bind to or bundle actin filaments (Figs 5E and 6B). Instead, the ABD1 and ABD2 motifs could bind to but not bundle actin filaments (Figs 5F, 5G, 6C and 6E). The EF motif could form homodimers, facilitating the actin binding/bundling activities of ABD1 and ABD2 (Figs 5H, 5I and 6D–6F, S7 Fig). These findings shed light on how MoFim1 binds to and bundles actin filaments through its various domains.

The Spk in the hyphal apex is composed of a dense group of vesicles, ribosomes, microtubules, actin, and an amorphous or granular material of undefined nature [7, 11]. The Spk is thought to be associated with hyphal tip growth and vesicle transport, which was further verified by our drug treatment experiments (Fig 7B). Although the Spk is a highly dynamic structure maintained at the hyphal tip that moves during hypha growth, we still do not know exactly how the Spk forms or how vesicles are transported from the inner cytoplasm to the hyphal tip [15]. Here, we found that MoFim1 localized to both the actin at Spk and actin cables in *M*. *oryzae* hyphae (Fig 4A–4E). Knockout of *MoFim1* impaired the formation of both the actin at Spk and the actin cables, thus compromising hyphal tip growth (Fig 3). We noted that the *Mofim1* mutant could form an Spk and establish the polar growth sometimes, but it cannot maintain the Spk and polar growth (Fig 3B). Therefore, we speculate that the knockout of *MoFim1* mainly prevents the maintenance of polar growth of hyphae. It appears that MoFim1 plays important roles in the establishment of actin structures originating from the Spk involved in hyphal tip growth in *M*. *oryzae*.

Polarized exocytosis is an essential process in fungi and is required for cell growth and pathogenesis [12]. This process involves the polarized trafficking of secretory vesicles to the Spk and their subsequent delivery to specific domains of the plasma membrane, where they fuse to provide the enzymes and material needed to build the cell wall and deliver proteins for virulence [7]. The role of the actin cytoskeleton in this continuous process has not been clearly elucidated. In the *Mofim1* mutant, the actin cytoskeleton was disorganized at the hyphal tip, indicating that vesicle delivery tracks were disordered (Fig 3). Thus, the subsequent transport of the v-SNARE protein Snc1 and the actin motor protein Myosin5 was disrupted (Fig 8), and protein secretion was compromised (S9 Fig and S1 Table). Based on these findings, we conclude that MoFim1 organizes the actin cytoskeleton in the hyphal tip, providing the tracks for polarized exocytosis. These important insights shed light on the molecular mechanism underlying how secretory vesicles are trafficked to the hyphal tips.

### *MoFim1* represents a target for *M*. *oryzae* control

Rice blast caused by *M*. *oryzae* is the most devastating fungal disease of rice [53]. Therefore, it is critical to develop effective means to control this disease. Numerous rice resistance (*R*) and defense-regulator (*DR*) genes conferring resistance to *M*. *oryzae* have been identified. For example, ~25 blast *R* genes, including the recently characterized *Pigm* gene from rice cultivar Gumei4, confer blast resistance [54, 55]. In addition, multiple *DR* genes that function in blast resistance have been identified, such as *bsr-d1*, *bsr-k1*, *spl11*, *spl33*, *IPA1*, and so on [56–60]. These important *R* and *DR* genes have potential applications for breeding rice cultivars with enhanced *M*. *oryzae* resistance.

In addition to these rice *R* or *DR* genes, key genes that function in *M*. *oryzae* development or pathogenesis could serve as targets for the control of fungal diseases via HIGS [39, 61]. Here, we investigated the physiological roles of MoFim1 and found that this protein plays crucial roles in actin cytoskeleton organization in *M*. *oryzae* during development and virulence. Moreover, transgenic rice produced in our HIGS experiments exhibited strong resistance to *M*. *oryzae* (Fig 10). This resistance was most likely due to the downregulated expression of *MoFim1* (Fig 10I), which would lead to disorganization of the actin cytoskeleton in the invasive hyphae, thereby compromising hyphal tip growth and expansion in rice cells (Fig 10C and 10D). Since this fimbrin gene is highly conserved in *M*. *oryzae*, it might represent an ideal target for *M*. *oryzae* control.

## Materials and methods

### Plant materials and *Magnaporthe oryzae* culture

All *M*. *oryzae* isolates used in this study were derived from the WT strain Y34 (kindly provided by Prof. LiHuang Zhu, Institute of Genetics and Developmental Biology, Chinese Academy of Sciences). All strains were cultured on CM agar plates for growth and SRB medium for conidia production. Liquid CM was used to prepare the mycelia for DNA and RNA extraction. Y34 susceptible rice (*Oryza sativa* ssp. *japonica* cv. Nipponbare) was used for disease and transgenic analyses.

### Targeted *MoFim1* deletion and plasmid construction

The deletion of *MoFim1* in *M*. *oryzae* was generated using the standard one-step gene replacement method [35]. To obtain the *Mofim1* complemented strain, the sequence containing the *MoFim1* gene and 1.5-kb native promoter region was ligated to mCherry and cloned into the pKNTG binary vector.

To construct plasmids expressing Snc1-GFP, Myosin5-GFP, Sep3-GFP, Exo84-GFP, and Sec9-GFP, the related DNA fragments from the *M*. *oryzae* genome and the ~1.5-kb native promoter region were amplified and cloned into the pKNTG binary vector. To construct the Lifeact-GFP plasmid, the Lifeact DNA sequence was ligated to *GFP* and cloned into the PsulPH vector [62, 63].

The HIGS experiments mainly followed the method reported previously [39, 40]. The gene-specific DNA fragments (about 200 bp) for *MoFim1* and *GFP* were cloned into the plant RNAi pANDAHK35 vector in opposite orientations on either side of a GUS linker. Then the fragment containing the gene-specific DNA fragments in two opposite orientations and the GUS linker were amplified and cloned into the pCAMBIA1300 vector and the recombinant plasmids were used for rice transformation. All constructs were generated via homologous recombination cloning (ClonExpress MultiS One Step Cloning Kit, Vazyme Biotech, C112); all primers with restriction enzyme sites are listed in S2 Table. The recombinant plasmids were transformed into *M*. *oryzae* protoplasts as described previously [35].

### Pathogenicity assay

The *M*. *oryzae* WT, *Mofim1*, and complemented strain were cultured on SRB medium for 7 d. As *Mofim1* could not generate spores, the same region of the culture plate for all three strains was used to infect punched rice leaves (the third leaf of each seedling, cv. Nipponbare). The disease phenotypes of the leaves were observed and imaged at 5 days post infection (dpi).

For spray inoculation of conidia, a conidial suspension (1 × 10^5^ conidia/ml) was sprayed onto rice leaves (WT and HIGS transgenic plants) with a sprayer. Inoculated plants were grown in a growth chamber at 28°C with high humidity in the dark for the first 24 h, followed by a 12-h/12-h light (20,000 lux)/dark cycle [64].

To infect rice sheath cells, *M*. *oryzae* spores (1 × 10^5^ conidia/ml) from WT and the *MoFim1* RNAi strains were diluted in a 0.2% (w/v) gelatin solution. The inner leaf sheath cuticle cells of 3-week-old rice plants were inoculated with the conidial suspension and incubated under humid conditions at 28°C.

### Measurement of the hyphae growth rate

The hyphae of the WT, *Mofim1* mutant, and the complemented strains were cultured in liquid CM overnight. The hyphae of the WT, *Mofim1* and the complemented strains were observed under a microscope (Zeiss LSM880, with a 20x objective). Time-lapse imaging was conducted and the hypha’s growth rate was calculated according to the growth distance over 60 minutes.

### Yeast two-hybrid assay

To validate homodimer formation of the EF domain, a yeast two-hybrid assay was performed using the Matchmaker Yeast Two-Hybrid System (Clontech) following the manufacturer’s instructions. The cDNA encoding the EF domain was cloned into the AD and BD vectors. The recombinant plasmids were cotransformed into AH109 cells and their growth examined on DDO and QDO medium. The pGADT7-T/pGBKT7-53 (AD-T/BD-53) plasmid was used as a positive control, and pGADT7/pGBKT7 (AD/BD) was used as a negative control.

### *In vitro* protein purification and F-actin binding/bundling assay

cDNAs from *MoFim1* and its various domains (shown in Fig 5A) were fused to *GFP* and cloned into the pET28a vector to produce His-tagged MoFim1 and MoFim1-GFP, EF-GFP, ABD1-GFP, EF-ABD1-GFP, ABD2-GFP and EF-ABD2-GFP fusion proteins. All primers and restriction enzyme sites are listed in S2 Table. All constructs were transformed into *Escherichia coli* strain BL21 (DE3), and recombinant protein expression was induced by the addition of 0.5 mM isopropyl-β-D-thiogalactoside at 16°C overnight. Recombinant proteins were purified following the manufacturer’s instructions.

The high- and low-speed co-sedimentation assays were conducted as previously described [25]. F-actin was prepared from rabbit muscle G-actin proteins as previously reported [26]. Briefly, G-actin (4 μM) was incubated at 22°C for 60 min alone or with 0 to 8 μM MoFim1 in KMEI buffer (10× stock: 500 mM KCl, 10 mM MgCl_2_, 10 mM EGTA, and 100 mM imidazole, pH 7.0). The samples were centrifuged at 200,000 *g* for 60 min for the high-speed co-sedimentation assay or 13,500 *g* for 30 min for the low-speed co-sedimentation assay. The proteins in the supernatants and pellets were separated by SDS-PAGE and visualized by staining with Coomassie Brilliant Blue R 250.

Visualization of actin filaments in the presence of MoFim1 and its domains was performed by fluorescence microscopy as reported previously [65]. Prepolymerized F-actin (1 μM) was incubated with MoFim1 and various truncated proteins (1 μM) at room temperature for 30 min and labeled with Alexa561-phalloidin (Thermo Fisher, A12380). The images were obtained under a confocal microscope (Zeiss LSM880 Airyscan) at 488 or 561 nm.

### Hyphae FM4-64 staining and drug treatment

FM4-64 solution (Thermo Fisher, T3166) was prepared as described previously [66]. *M*. *oryzae* mycelia were cultured in liquid CM for 24 h. The hyphae were stained with FM4-64 (10 μM) before being viewed by fluorescence microscopy. The actin inhibitor Lat B (Invitrogen, L22290) was added to the culture 30 min before FM4-64 staining.

### Extraction of secreted proteins from *M*. *oryzae* mycelia

Secreted proteins were extracted from *M*. *oryzae* mycelia as described previously [12]. Fresh WT and *Mofim1* mycelia were cultured in liquid CM for 48 h. An equal weight of WT and *Mofim1* mycelia was harvested by filtration and transferred to liquid GMM for 24 h. The secreted proteins in the medium were collected and condensed in an ultrafiltration tube (3 kD, Millipore). The protein samples were quantified by the Bradford method.

### RNA extraction and qRT-PCR analysis

Total RNA was extracted from the samples using a Total RNA Purification kit (TransGen, ET101-01) according to the manufacturer’s protocol. qRT-PCR analysis of *GFP* and *MoFim1* in transgenic rice (generated by HIGS) was performed using SYBR Green Real-time PCR Master Mix (Toyobo, Japan), with *M*. *oryzae Histone* used as the internal control. All reactions were conducted in triplicate using the primers shown in S2 Table.

### Observation of fluorescent signals by super-resolution live-cell imaging

Live-cell imaging was conducted under a super-resolution confocal microscope (Zeiss LSM880) equipped with an Airyscan detector. The Airyscan unit acquires data simultaneously using 32 detectors arranged in a hexagonal array [67]. The images were processed and analyzed using ImageJ (http://rsbweb.nih.gov/ij), as described previously [68]. F-actin skewness and density were measured as previously described [69]. The maximum projection of the image stack was used to record the global organization of the actin cytoskeleton in fast-growing *M*. *oryzae* hyphal cells.

### Southern blot analysis

For *MoFim1* deletion verification, Kpn I was used to digest the genomic DNA from the WT and the *Mofim1* mutant. The digested products were separated, blotted to a membrane, and hybridized with the two indicated biotin-labeled probes (S3 Fig). The probe was designed according to the disruption strategy and was amplified from genomic DNA. To confirm HIGS-*MoFim1* transgenic rice, the biotin-labeled probe was used to hybridize to BamH I-digested genomic DNA from the WT and the transgenic rice plants. All the primers used in the Southern blot experiments are listed in S2 Table. The detection was carried out according to the manufacturer’s instructions (Thermo DNA Detection kit, 20148).

### Statistical analysis

Skewness analysis was performed to quantify the extent of actin bundling in hyphae according to a previously described method [70, 71]. The z-series stacks of all optical sections were filtered using Gaussian blur to reduce background noise and then skeletonization was assessed with ThinLine, a JAVA plug-in procedure [70]. The actin filament pixels were collected into a single image using maximum intensity projections and the skewness values were calculated. The actin filament counts in hyphae, the area of the diseased rice leaves, and the fluorescence intensity of the images were obtained using the ImageJ/Fiji platform (http://rsbweb.nih.gov/ij). Then two-tailed *t*-tests were used to determine the significance of results. The numerical data and statistical analysis that were used to generate graphs were provided in S1 Data.

### Accession numbers

Sequence data for the genes described in this study can be found in the GenBank/EMBL database under the accession numbers: *MoFim1* (ELQ33056), *Snc1* (ELQ36245), *Myosin5* (ELQ36095), *Sep3* (ELQ45022), *Exo84* (ELQ40036), *Sec9* (ELQ38864).

## Supporting information

S1 Fig Construction of the *Mofim1* knockout mutant. **(A)** Schematic representation of the recombination event involved in the targeted replacement of *MoFim1*. **(B)** PCR identification of the knockout mutant and complemented strain using the primers indicated in **(A)**. Lanes 1, 2, and 3 indicate the *Mofim1* mutant, WT and the complemented strain, respectively.
(TIF)

S2 Fig Hyphae growth rate of the WT, *Mofim1* mutant, and the complemented strain on CM agar plates. The diameters of the cultured WT, *Mofim1* mutant, and complemented strain were measured for 7 days. Error bars indicate standard deviation calculated for three replicates.
(TIF)

S3 Fig Verification of the *Mofim1* mutant. **(A)** Seven-day-old cultures of the WT, three purified *Mofim1* single colonies from the protoplasts, and the corresponding complemented strains on SRB medium. **(B)** Southern blot analysis of the *Mofim1* gene deletion mutants with a gene-specific probe (probe 1) or *hygromycin phosphotransferase* (*HPH*) probe (probe 2). Black lines below the arrows indicate sequence-specific gene probes.
(TIF)

S4 Fig The dynamic assembly of actin in WT and the *Mofim1* mutant revealed by FRAP analysis. **(A)** Images were recorded by FRAP analysis before bleaching, immediately after bleaching, and 19, 104, and 200 s after bleaching. Bars = 2 μm. Images are related to S6 Movie. **(B)** Quantitative FRAP analysis in WT (black curve) and *Mofim1* cells (red curve). The fluorescence at *t*_1/2_ was graphically determined: 19.2 s for WT and 103.4 s for *Mofim1*.
(TIF)

S5 Fig Localization of MoFim1 in invasive hyphae. *Mofim1* mutant expressing both *Lifeact-GFP* and p*MoFim1*-*MoFim1-mCherry* were used in the penetration assay. Three-week-old rice (*O*. *sativa* ssp. *japonica* cv. Nipponbare) was inoculated with fluorescently labeled spores on the inner leaf sheath cells. Photographs were taken at 12 h after infection. Bar = 5 μm.
(TIF)

S6 Fig Biochemical analysis of the MoFim1 actin binding and assembly. **(A and B)** High-speed **(A)** and low-speed **(B)** co-sedimentation assays showing the actin binding or bundling activity of MoFim1. F-actin (4 μM) was incubated with increasing amounts of MoFim1 (0–8 μM). The samples were centrifuged at 200,000 *g* (high speed) or 13,500 *g* (low speed), and the pellets and supernatants were separated by SDS-PAGE.
(TIF)

S7 Fig Yeast two-hybrid assay showing that the EF domain of MoFim1 forms homodimers. To determine whether the EF domain could form homodimers, yeast cells containing the indicated plasmids were grown on SD/-Leu/-Trp DO (DDO) plates and SD/-Leu/-Trp/-Ade/-His DO (QDO) plates (containing 40 mg/L X-α-gal) for 3 d. Interactions of AD/BD, AD/BD-EF, AD-EF/BD were used as the negative controls, and AD-T/BD-53 was used as the positive control.
(TIF)

S8 Fig Plant infection analysis of the *Mofim1* mutant and the complemented strain transformed with truncated *MoFim1*. **(A)** Seven-day-old cultures of the WT, the *Mofim1* mutant, and the complemented strains transformed with *MoFim1* or the indicated truncated *MoFim1*. **(B)** Pathogenicity assay using WT, *Mofim1*, and the complemented *M*. *oryzae* strains indicated in **(A)**. The same area of each SRB culture plate from the indicated strain was used to infect these rice leaves (*O*. *sativa* cv. Nipponbare). Photographs were taken 5 d after infection. **(C)** Quantification of the lesion area of the rice leaves shown in **(B)**. Error bars represent SD (*n* = 20) and the asterisks represent significant difference (***, *P* < 0.001).
(TIF)

S9 Fig Analysis of protein secretion in *Mofim1*. Equal amounts of mycelia from WT and *Mofim1* were cultured in liquid GMM for 24 h. The supernatants were collected and condensed. Total secreted proteins were measured by the Bradford method. Error bars show ± SD of the means for three biological repetitions of the experiment. Asterisks indicate statistically significant differences, as determined by Student’s *t*-test (**, *P* < 0.01).
(TIF)

S10 Fig Penetration analysis of the *MoFim1*-silenced RNAi-1 strain. Rice leaf sheath cells were inoculated with WT **(A)** or RNAi-1 **(B)** *M*. *oryzae* spores. Photographs were taken 72 h after infection, Bars = 5 μm. **(C)** Quantification of the penetration of the WT and RNAi-1 *M*. *oryzae* spores. Error bars show SD of the means for three biological repetitions of the experiment (*n* = 100). Asterisks indicate statistically significant differences, as determined by Student’s *t*-test (**, *P* < 0.01).
(TIF)

S11 Fig Southern blot analysis of the HIGS-*MoFim1* transgenic rice plants. BamH I-digested genomic DNAs of WT and the HIGS-*MoFim1* transgenic rice plants were hybridized with a 5’-Biotin labeled DNA fragment indicated in the figure.
(TIF)

S1 Table MS identification of the secreted proteins from WT and the *Mofim1* mutant. (DOC)

S2 Table Primers used in this study. (DOC)

S1 Movie The dynamic organization of the actin cytoskeleton in growing hyphae. Related to Fig 2A. This representative video is based on data from 50 mycelia in three independent experiments. The numbers at the top right corner indicate the timestamps (min:s). Bar = 5 μm.
(AVI)

S2 Movie 3D reconstruction of actin organization in *M*. *oryzae* hyphae. Related to Fig 2B and 2C. This representative video is based on data from 30 mycelia in three independent experiments.
(AVI)

S3 Movie The dynamic transport of actin patches along the actin cables. Related to Fig 2E. This representative video was obtained from S1 Movie. The red arrows indicate the actin patches. The numbers at the top right corner indicate the timestamps (min:s). Bar = 2 μm.
(AVI)

S4 Movie The dynamic organization of the actin cytoskeleton in growing *Mofim1* mycelia. Related to Fig 3. This representative video is based on data from 30 mycelia in three independent experiments. Red arrows indicate the presence of actin at the Spk in the WT **(A)**, the *Mofim1* mutant **(B)** and the complemented strain **(C)**. The numbers at the top right corner indicate the timestamps (min:s). Bar = 5 μm.
(AVI)

S5 Movie Hyphal growth analysis of the *Mofim1* by microscopy. The hypha of the WT **(A)**, *Mofim1* **(B)** and the complemented **(C)** strains were observed under a microscope (Zeiss LSM880, with a 20x objective). Time-lapse imaging was conducted and the hyphal growth rate was calculated according to the distance it grew. This representative video is based on data from 20 mycelia in three independent experiments. The numbers at the top right corner indicate the timestamps (min:s). Bar = 25 μm.
(AVI)

S6 Movie FRAP analysis of actin dynamics in *Mofim1*. Related to S4 Fig. The red arrows indicate the presence of the actin cytoskeleton at the hyphal tip, and the yellow asterisk shows the bleaching point. The numbers at the top right corner indicate the timestamps (min:s). Bar = 2 μm.
(AVI)

S7 Movie Localization analysis of MoFim1 in growing vegetative hyphae. Related to Fig 4A. Lifeact-GFP **(A)** and MoFim1-mCherry **(B)** driven by its native promoter were coexpressed in the *Mofim1* mutant. Arrows indicate the Spk. This representative video is based on data from 50 mycelia in three independent experiments. The numbers at the top right corner indicate the timestamps (min:s). Bar = 5 μm.
(AVI)

S8 Movie Localization analysis of MoFim1 in growing invasive hyphae. Related to Fig 4B and S5 Fig. Lifeact-GFP and MoFim1-mCherry driven by its native promoter were coexpressed in the *Mofim1* mutant and used for rice sheath cell penetration. The red arrowhead indicates the spot when the imaging starts. This representative video is based on data from 20 mycelia in three independent experiments. The numbers at the top right corner indicate the timestamps (min:s). Bar = 2 μm. Bar = 5 μm.
(MOV)

S9 Movie The dynamic transport of Snc1 in growing WT and *Mofim1* mycelia. Related to Fig 8A. Snc1-GFP driven by its native promoter was expressed in WT and the *Mofim1* mutant. This representative video is based on data from 20 mycelia in three independent experiments. The numbers at the top right corner indicate the timestamps (min:s). Bar = 5 μm.
(AVI)

S10 Movie The dynamic transport of Myosin 5 in growing WT and *Mofim1* mycelia. Related to Fig 8B. Myosin5-GFP driven by its native promoter was expressed in the WT and the *Mofim1* mutant. This representative video is based on data from 20 mycelia in three independent experiments. The numbers at the top right corner indicate the timestamps (min:s). Bar = 5 μm.
(AVI)

S11 Movie The dynamic transport of Sep3 in growing WT and *Mofim1* mycelia. Related to Fig 8C. Sep3-GFP driven by its native promoter was expressed in the WT and the *Mofim1* mutant. This representative video is based on data from 20 mycelia in three independent experiments. The numbers at the top right corner indicate the timestamps (min:s). Bar = 5 μm.
(AVI)

S12 Movie The dynamic transport of Exo84 and Sec9 in growing WT and *Mofim1* mycelia. Related to Fig 8D and 8E. Exo84-GFP and Sec9-GFP driven by their native promoters were expressed in WT and the *Mofim1* mutant. This representative video is based on data from 20 mycelia in three independent experiments. The numbers at the top right corner indicate the timestamps (min:s). Bar = 5 μm.
(AVI)

S1 Data The numerical data and statistical analysis that were used to generate graphs in the manuscript. (XLS)
