# Correction: *Magnaporthe oryzae* fimbrin organizes actin networks in the hyphal tip during polar growth and pathogenesis

**DOI:** 10.1371/journal.ppat.1012210

**Published:** 2024-05-06

**Authors:** Yuan-Bao Li, Rui Xu, Chengyu Liu, Ningning Shen, Li-Bo Han, Dingzhong Tang

The images for GFP in Fig 5B and EF-GFP in Fig 5E appear blank due to low fluorescence signal in these samples and because the images are presented using the default settings of the Zeiss microscopy software. When exported using ‘best fit’ mode to auto-optimize contrast and brightness, a weak fluorescent signal is visible in these panels.

In addition, some of the labels in Fig 5B–5I were placed over previous labels from an earlier version of the figure.

An updated version of Fig 5 is provided with this notice containing ‘best fit’ mode microscopy images for all samples and with the label issue resolved. S1 File contains ‘default’ and ‘best fit’ mode images for Fig 5.

**Fig 5:**
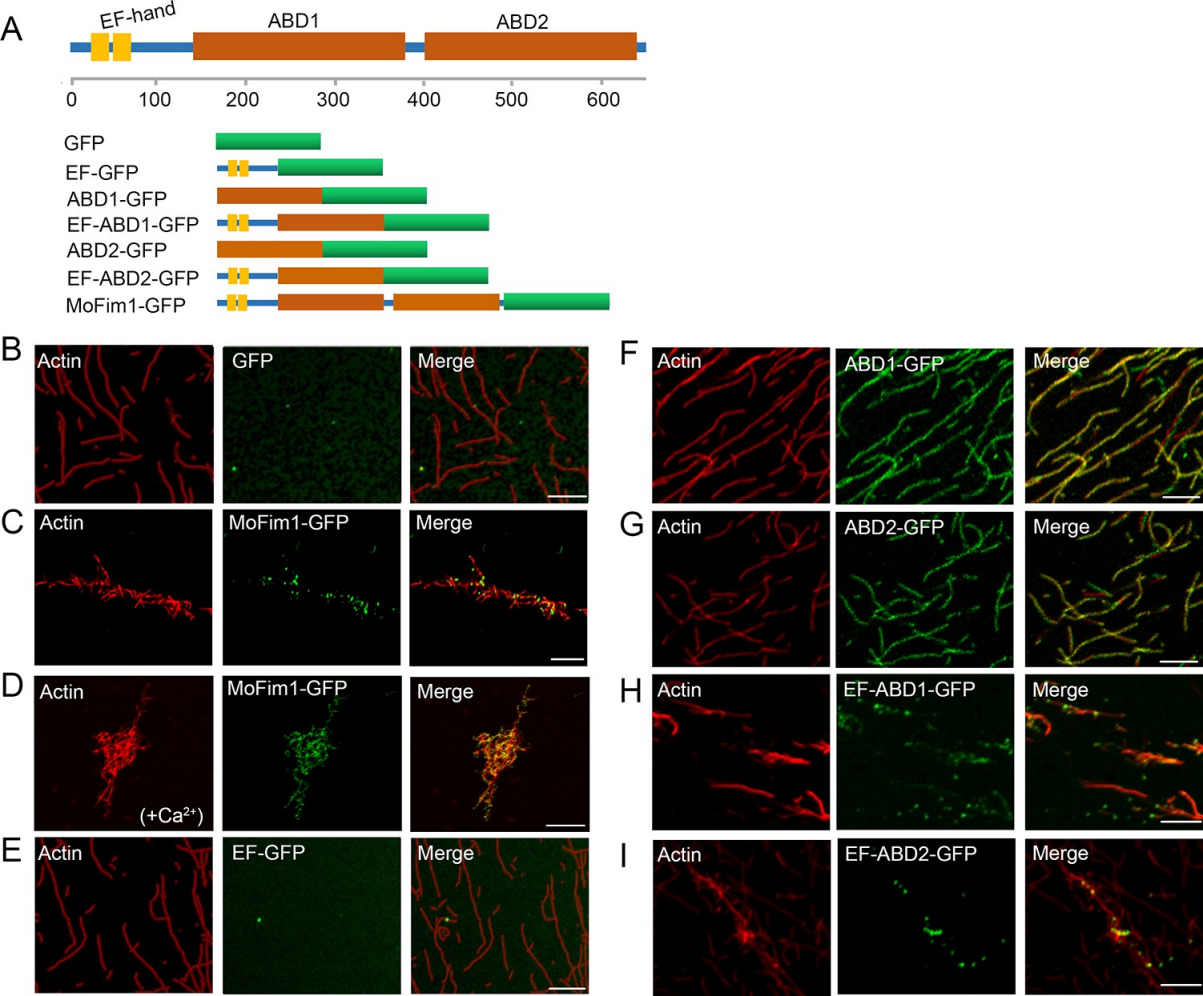
Biochemical analysis of MoFim1. **(A)** Schematic diagram showing the protein domains of MoFim1 and GFP-fused MoFim1 and truncated proteins. **(B–I)** Actin binding/bundling analysis of MoFim1 and its various domains by fluorescence observation. Polymerized F-actin (1 μM) incubated with various proteins (1 μM) was used for analysis. Alex561-phalloidin-labeled F-actin alone **(B)**; F-actin in the presence of MoFim1-GFP **(C)**; F-actin in the presence of MoFim1-GFP and Ca^2+^ (1 μM) **(D)**; F-actin in the presence of EF-GFP **(E)**; ABD1-GFP **(F)**; ABD2-GFP **(G)**; EF-ABD1-GFP **(H)** and EF-ABD2-GFP **(I)**. Bars = 1 μm.

In addition, the article’s Data Availability statement was incorrect and is updated to: The original underlying data to support all results in the article and Supporting Information files are available from the corresponding author. [1]

The authors apologize for the error in the published article.

## Supporting information

S1 File Original microscopy images in ‘default’ and ‘best fit’ mode for Fig 5. (ZIP)
