# Supplementary figures and images for: Magnaporthe oryzae fimbrin organizes actin networks in the hyphal tip during polar growth and pathogenesis
Source: PLoS Pathog. 2020 Mar 16;16(3):e1008437. doi: 10.1371/journal.ppat.1008437 (PMC7098657; doi:10.1371/journal.ppat.1008437)

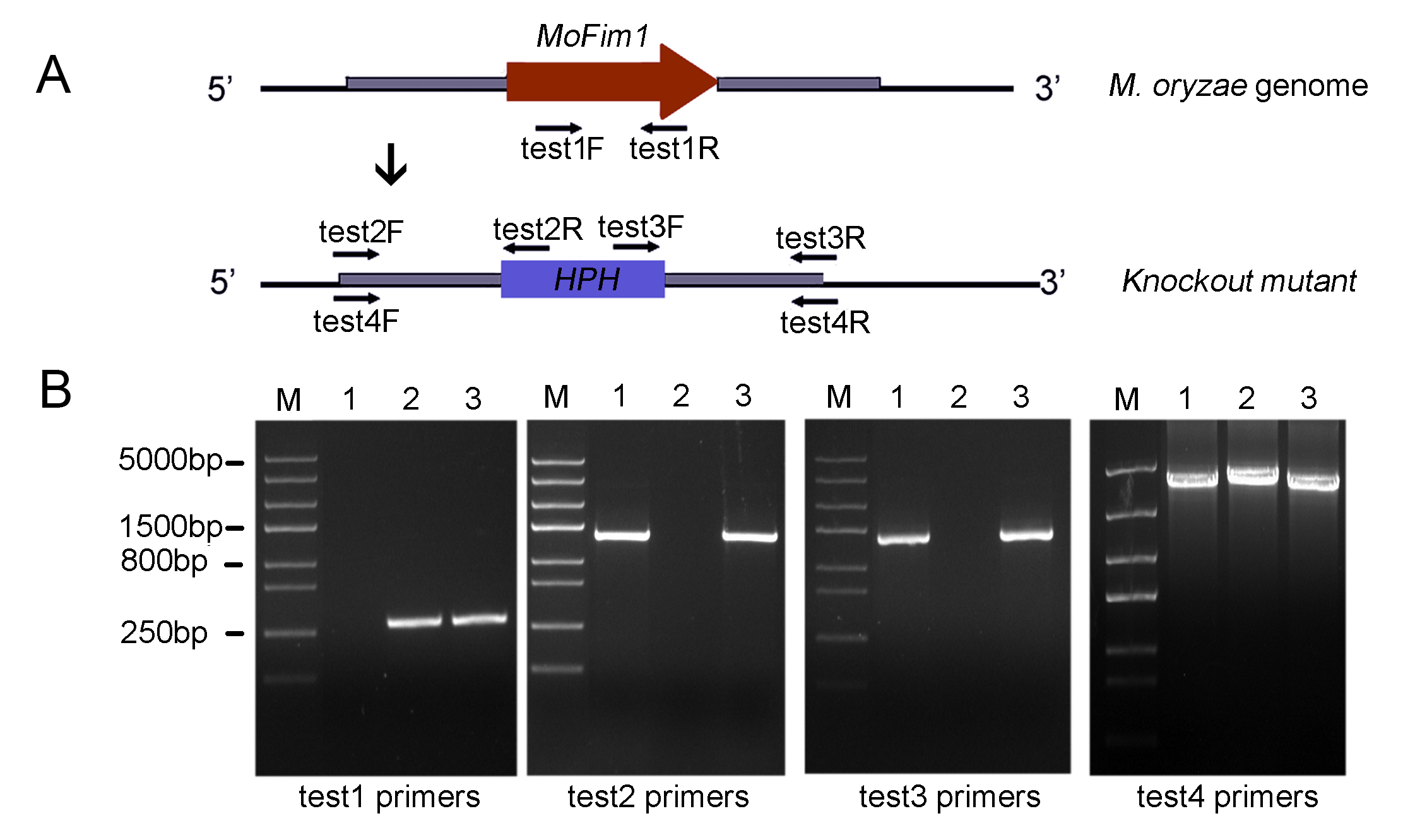

Supplement: S1 Fig — (A) Schematic representation of the recombination event involved in the targeted replacement of MoFim1. (B) PCR identification of the knockout mutant and complemented strain using the primers indicated in (A). Lanes 1, 2, and 3 indicate the Mofim1 mutant, WT and the complemented strain, respectively. (TIF) [file ppat.1008437.s001.tif]

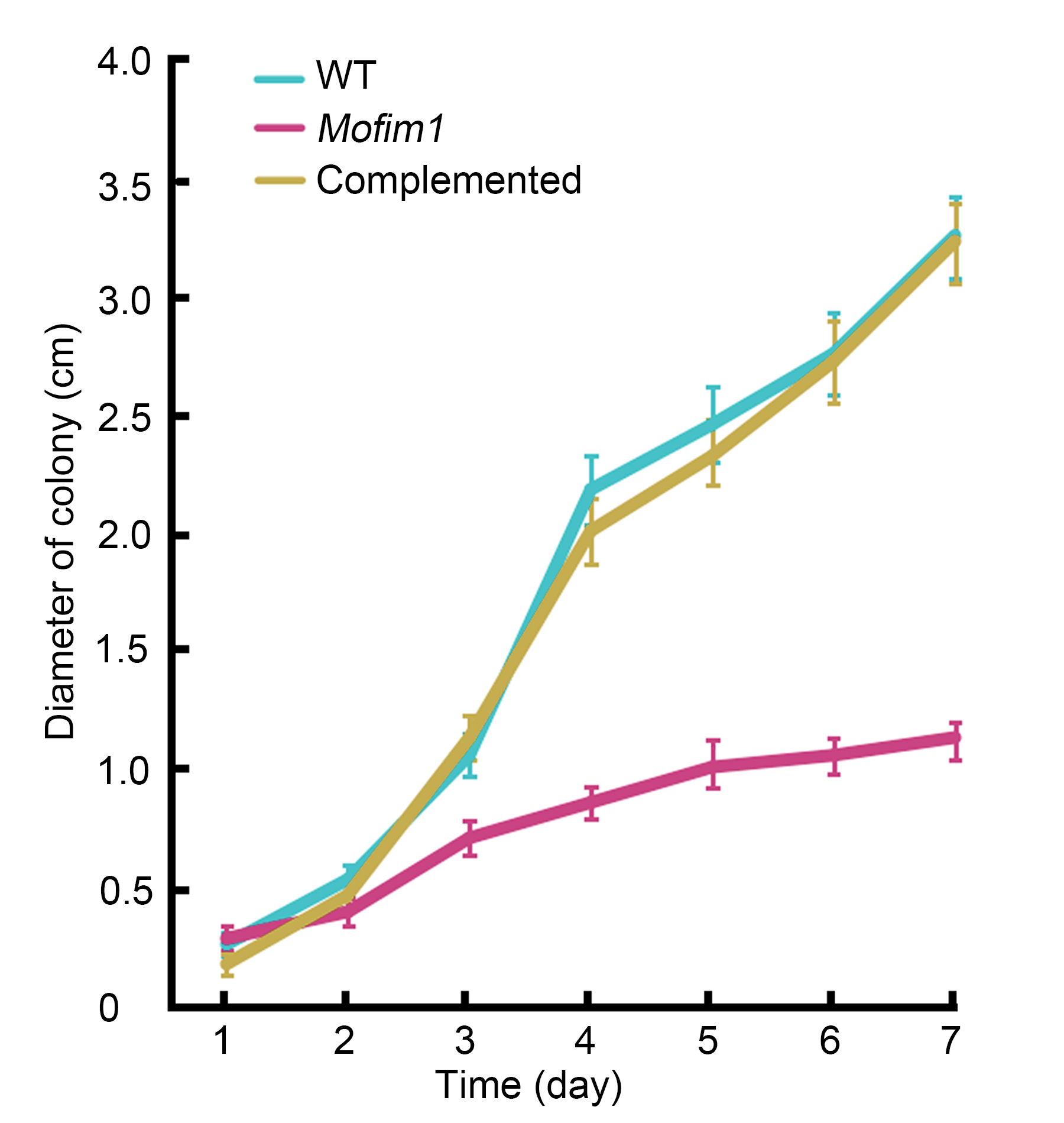

Supplement: S2 Fig — The diameters of the cultured WT, Mofim1 mutant, and complemented strain were measured for 7 days. Error bars indicate standard deviation calculated for three replicates. (TIF) [file ppat.1008437.s002.tif]

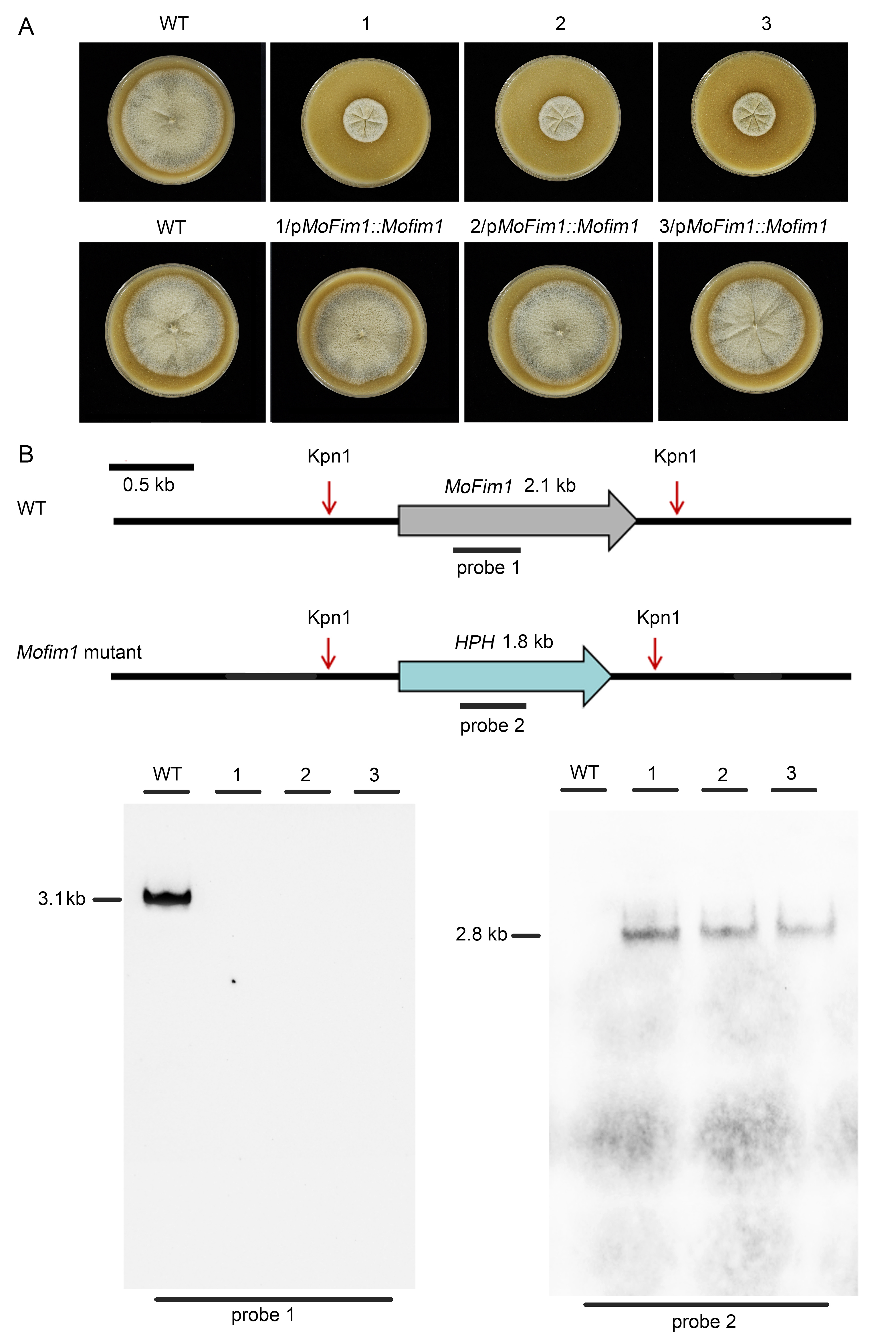

Supplement: S3 Fig — (A) Seven-day-old cultures of the WT, three purified Mofim1 single colonies from the protoplasts, and the corresponding complemented strains on SRB medium. (B) Southern blot analysis of the Mofim1 gene deletion mutants with a gene-specific probe (probe 1) or hygromycin phosphotransferase (HPH) probe (probe 2). Black lines below the arrows indicate sequence-specific gene probes. (TIF) [file ppat.1008437.s003.tif]

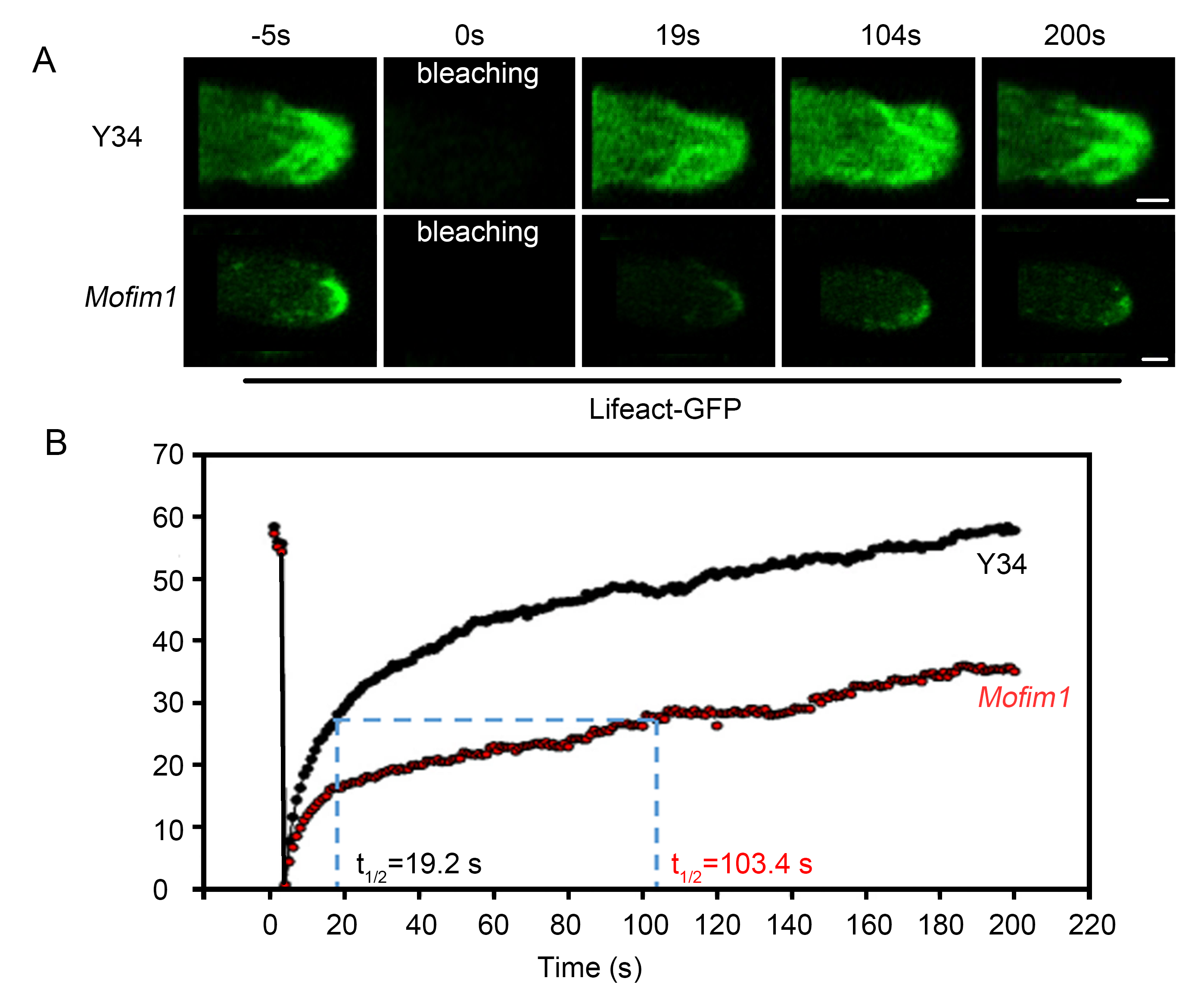

Supplement: S4 Fig — (A) Images were recorded by FRAP analysis before bleaching, immediately after bleaching, and 19, 104, and 200 s after bleaching. Bars = 2 μm. Images are related to S6 Movie. (B) Quantitative FRAP analysis in WT (black curve) and Mofim1 cells (red curve). The fluorescence at t1/2 was graphically determined: 19.2 s for WT and 103.4 s for Mofim1. (TIF) [file ppat.1008437.s004.tif]

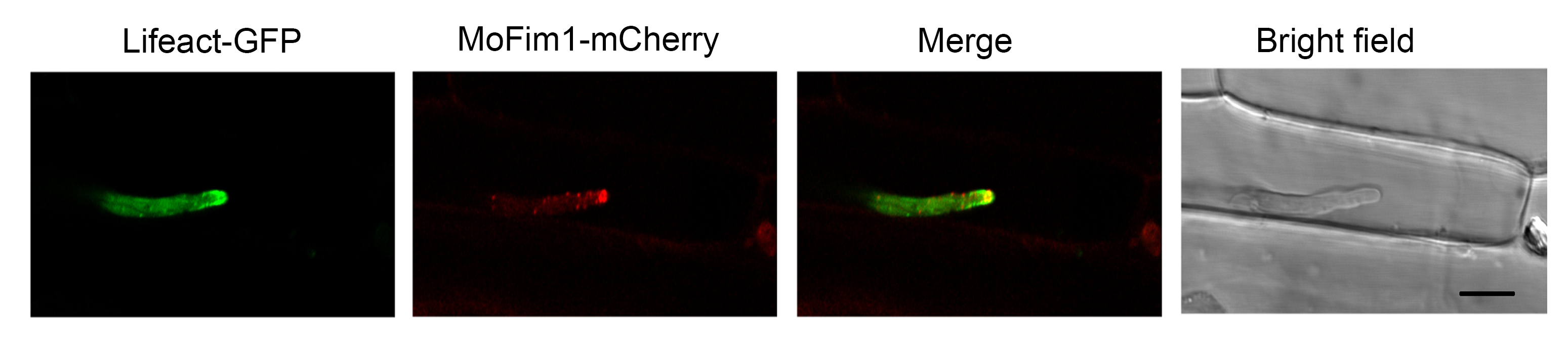

Supplement: S5 Fig — Mofim1 mutant expressing both Lifeact-GFP and pMoFim1-MoFim1-mCherry were used in the penetration assay. Three-week-old rice (O. sativa ssp. japonica cv. Nipponbare) was inoculated with fluorescently labeled spores on the inner leaf sheath cells. Photographs were taken at 12 h after infection. Bar = 5 μm. (TIF) [file ppat.1008437.s005.tif]

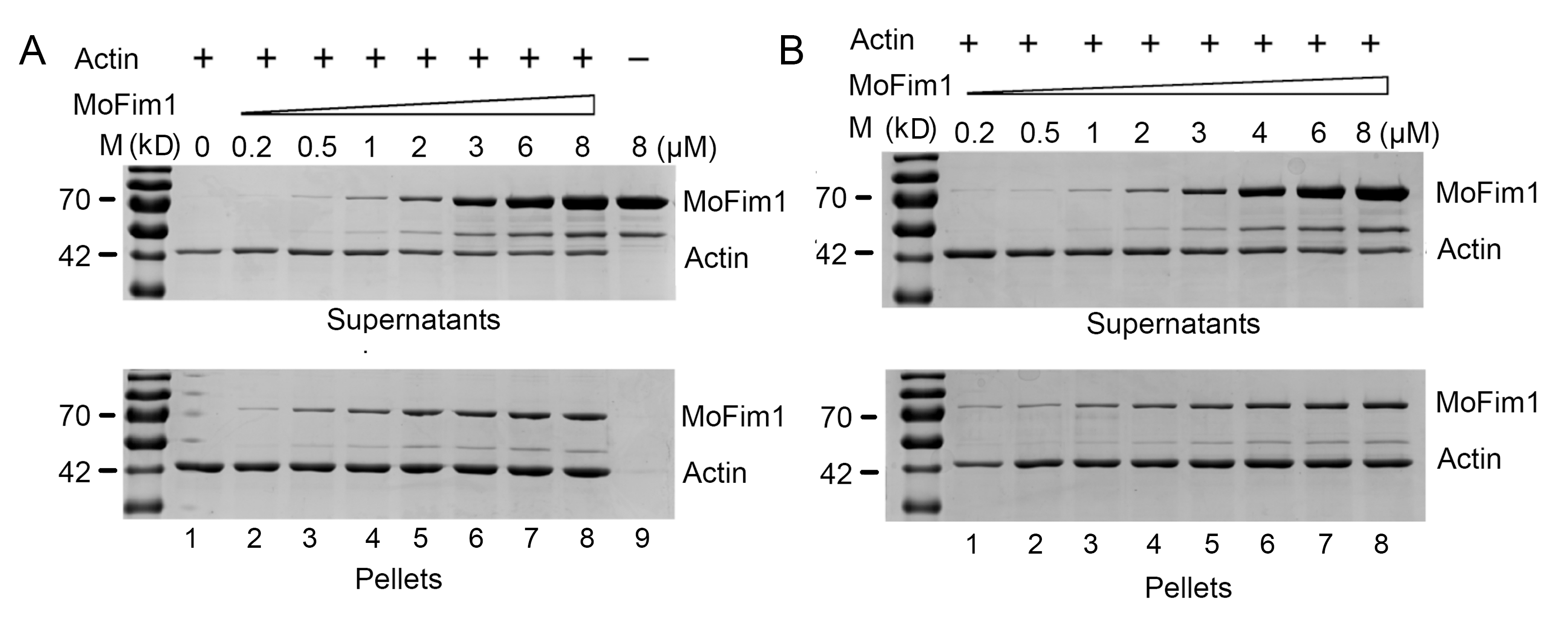

Supplement: S6 Fig — (A and B) High-speed (A) and low-speed (B) co-sedimentation assays showing the actin binding or bundling activity of MoFim1. F-actin (4 μM) was incubated with increasing amounts of MoFim1 (0–8 μM). The samples were centrifuged at 200,000 g (high speed) or 13,500 g (low speed), and the pellets and supernatants were separated by SDS-PAGE. (TIF) [file ppat.1008437.s006.tif]

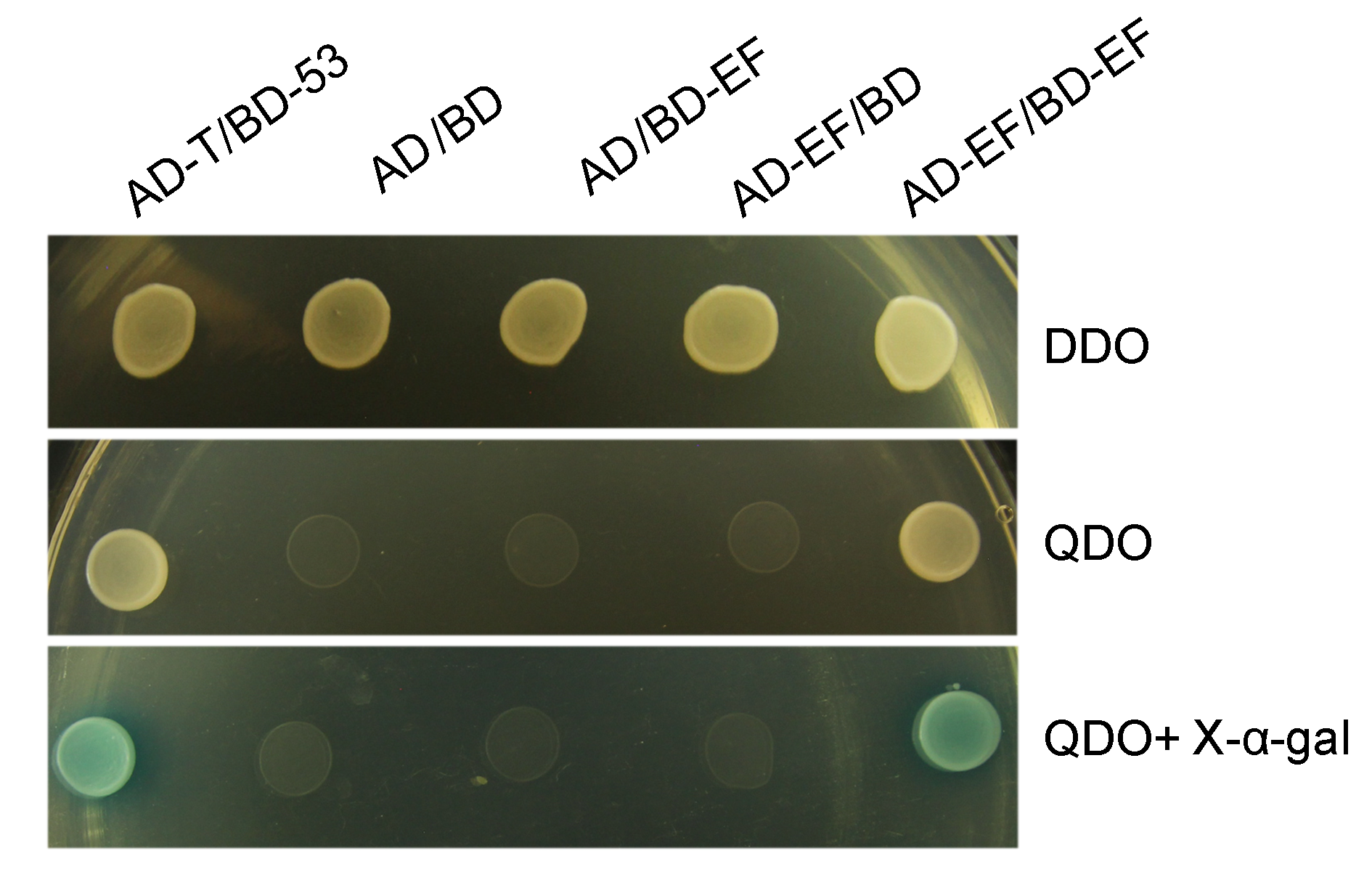

Supplement: S7 Fig — To determine whether the EF domain could form homodimers, yeast cells containing the indicated plasmids were grown on SD/-Leu/-Trp DO (DDO) plates and SD/-Leu/-Trp/-Ade/-His DO (QDO) plates (containing 40 mg/L X-α-gal) for 3 d. Interactions of AD/BD, AD/BD-EF, AD-EF/BD were used as the negative controls, and AD-T/BD-53 was used as the positive control. (TIF) [file ppat.1008437.s007.tif]

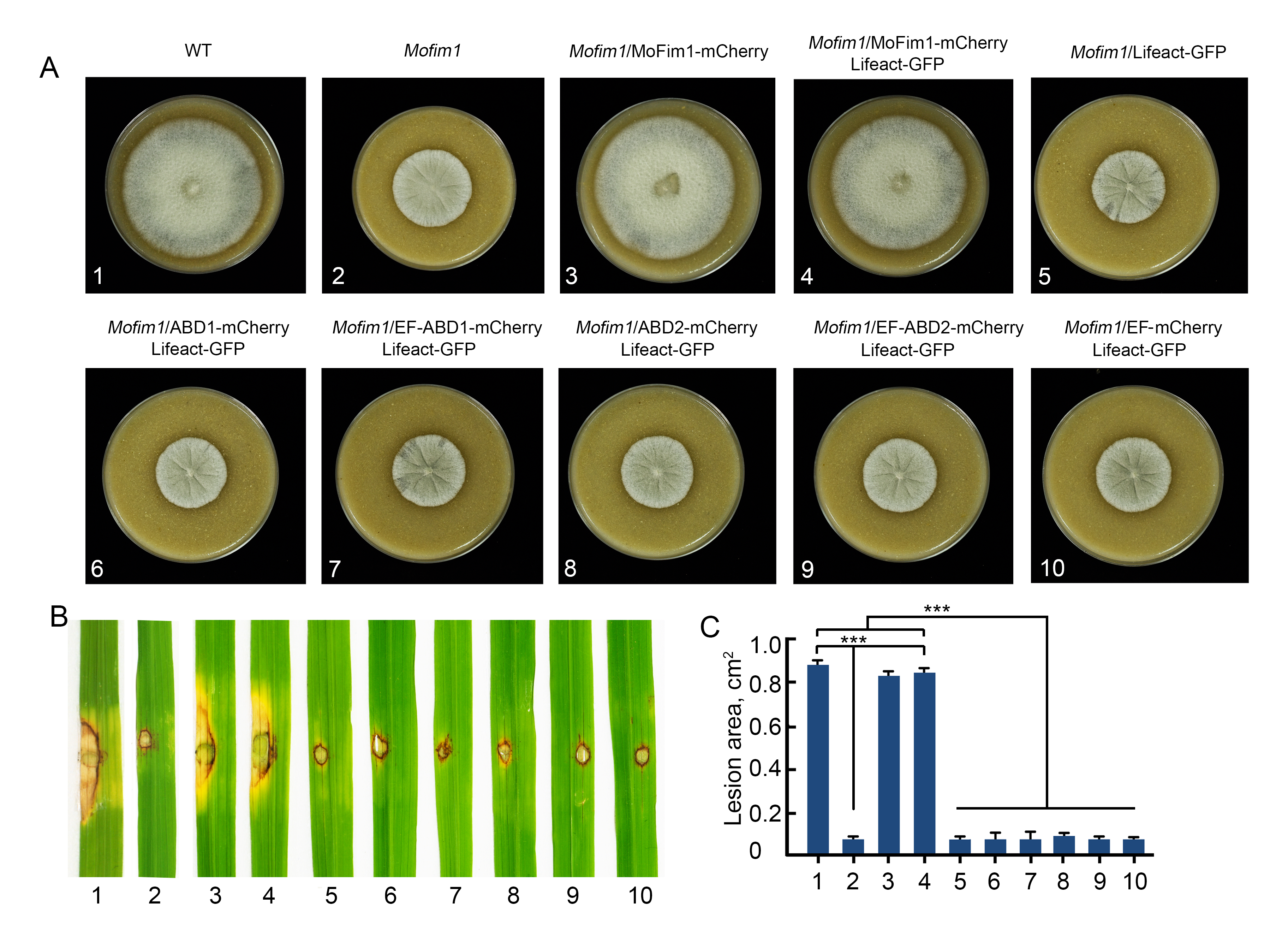

Supplement: S8 Fig — (A) Seven-day-old cultures of the WT, the Mofim1 mutant, and the complemented strains transformed with MoFim1 or the indicated truncated MoFim1. (B) Pathogenicity assay using WT, Mofim1, and the complemented M. oryzae strains indicated in (A). The same area of each SRB culture plate from the indicated strain was used to infect these rice leaves (O. sativa cv. Nipponbare). Photographs were taken 5 d after infection. (C) Quantification of the lesion area of the rice leaves shown in (B). Error bars represent SD (n = 20) and the asterisks represent significant difference (***, P < 0.001). (TIF) [file ppat.1008437.s008.tif]

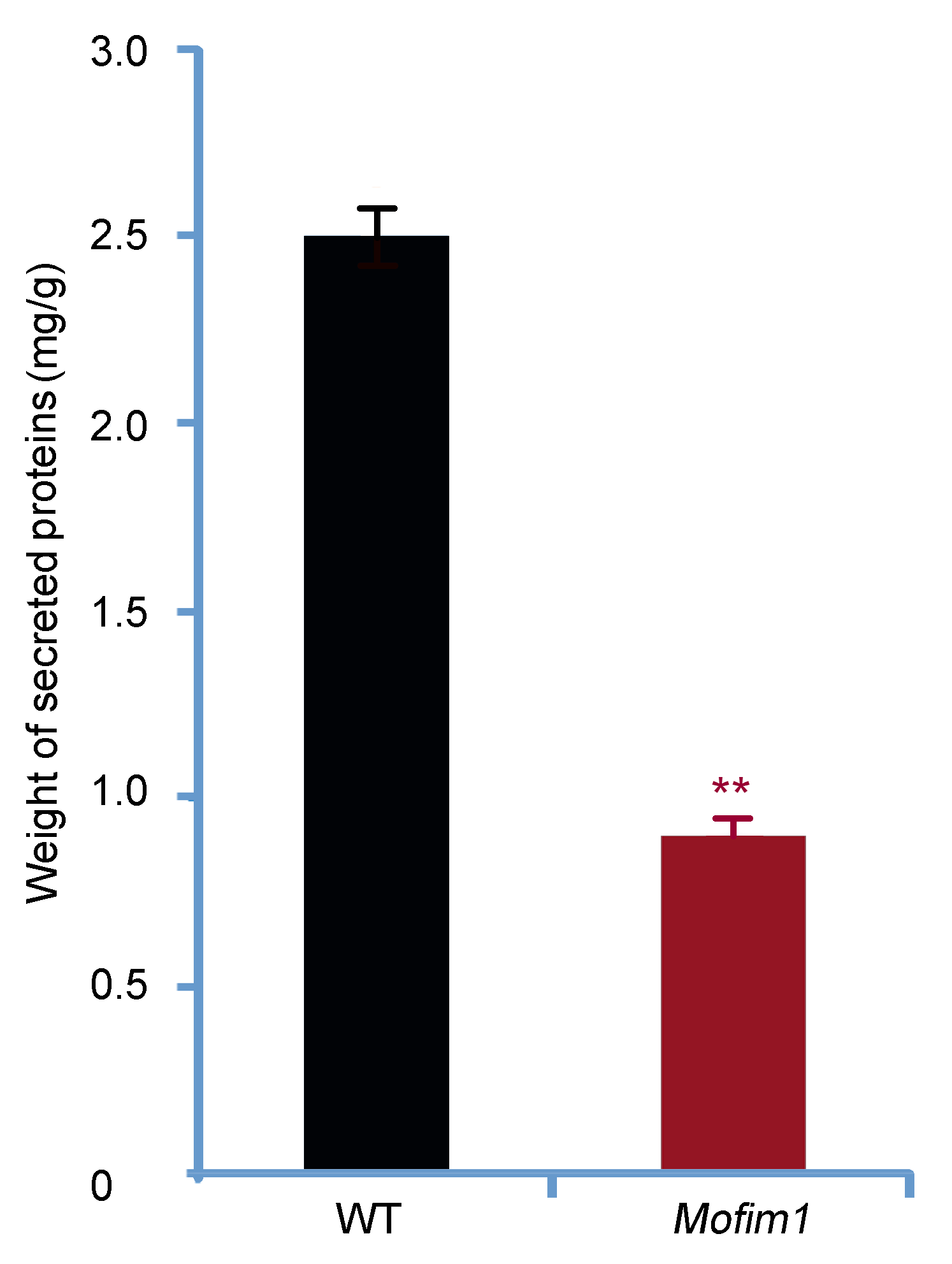

Supplement: S9 Fig — Equal amounts of mycelia from WT and Mofim1 were cultured in liquid GMM for 24 h. The supernatants were collected and condensed. Total secreted proteins were measured by the Bradford method. Error bars show ± SD of the means for three biological repetitions of the experiment. Asterisks indicate statistically significant differences, as determined by Student’s t-test (**, P < 0.01). (TIF) [file ppat.1008437.s009.tif]

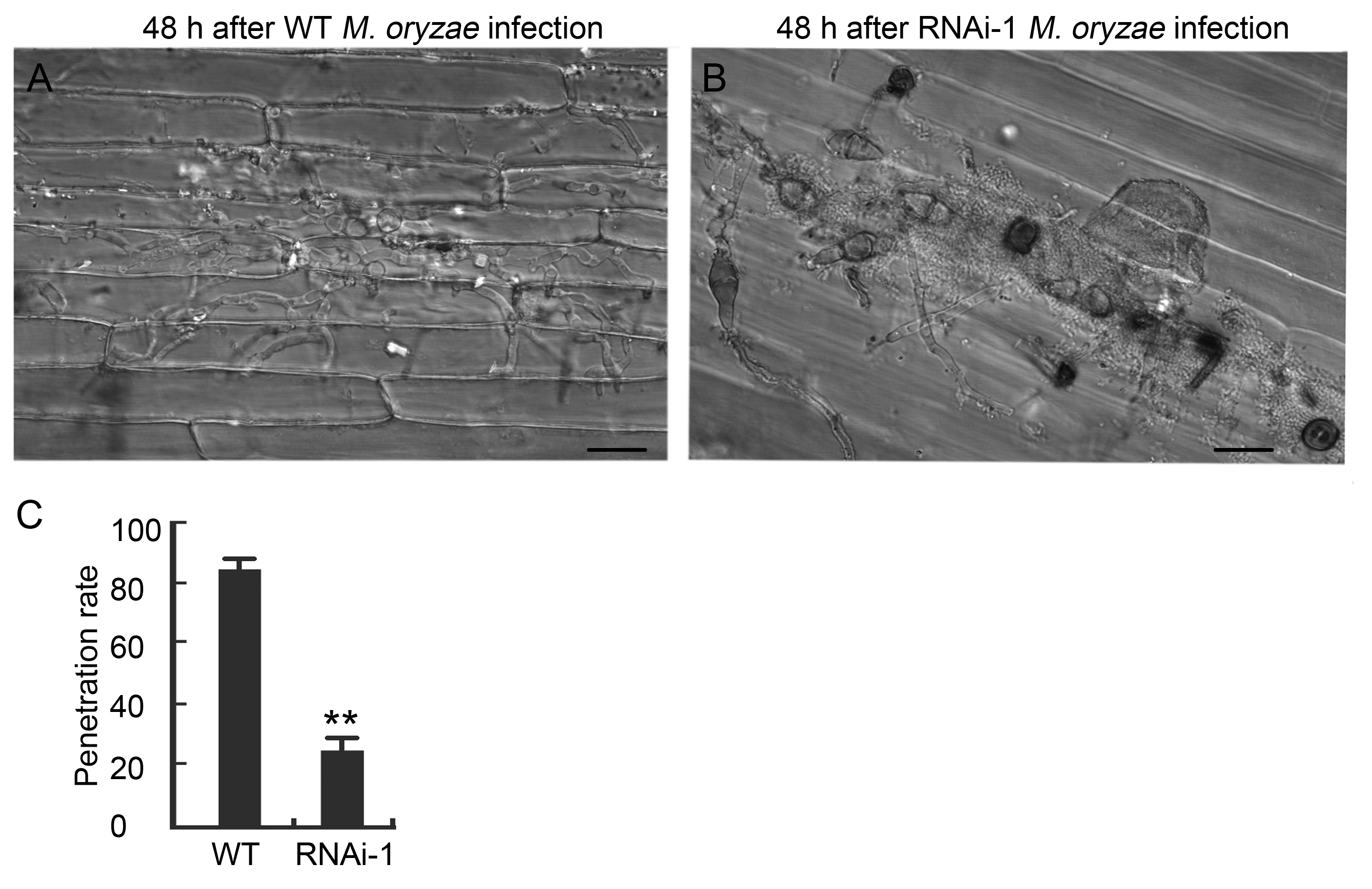

Supplement: S10 Fig — Rice leaf sheath cells were inoculated with WT (A) or RNAi-1 (B) M. oryzae spores. Photographs were taken 72 h after infection, Bars = 5 μm. (C) Quantification of the penetration of the WT and RNAi-1 M. oryzae spores. Error bars show SD of the means for three biological repetitions of the experiment (n = 100). Asterisks indicate statistically significant differences, as determined by Student’s t-test (**, P < 0.01). (TIF) [file ppat.1008437.s010.tif]

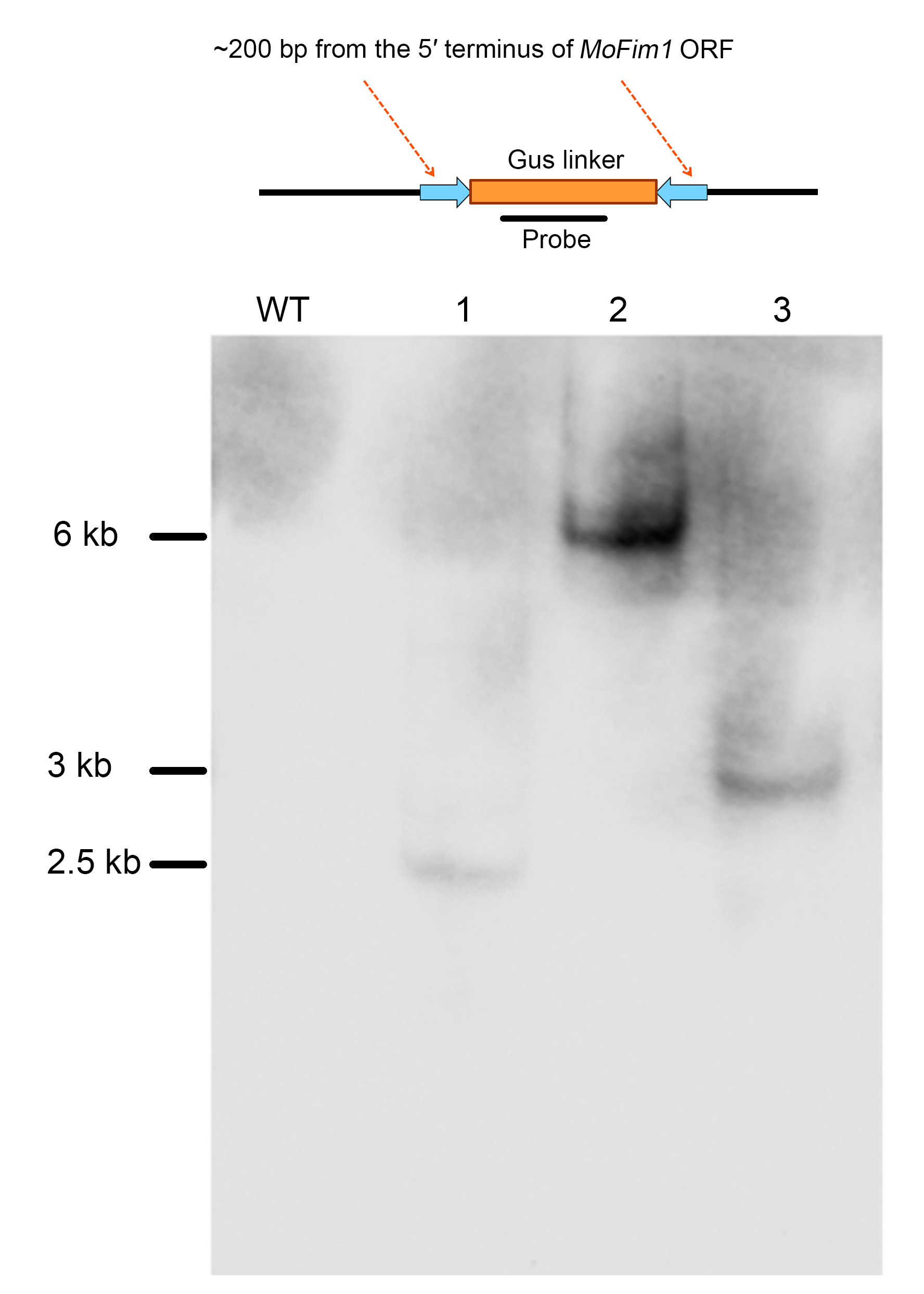

Supplement: S11 Fig — BamH I-digested genomic DNAs of WT and the HIGS-MoFim1 transgenic rice plants were hybridized with a 5’-Biotin labeled DNA fragment indicated in the figure. (TIF) [file ppat.1008437.s011.tif]
