# Supplementary material for: Magnaporthe oryzae fimbrin organizes actin networks in the hyphal tip during polar growth and pathogenesis
Source: PLoS Pathog. 2020 Mar 16;16(3):e1008437. doi: 10.1371/journal.ppat.1008437 (PMC7098657; doi:10.1371/journal.ppat.1008437)
Supplement: S1 Table — (DOC) [file ppat.1008437.s012.doc]

**S1 Table.** MS identification of the secreted proteins from WT and *Mofim1* mutant.

|  | Protein description | Protein ID | Changed fold (Mofim1/WT) | Secretory peptide | Amino acid number |
| --- | --- | --- | --- | --- | --- |
| 1 | Uncharacterized protein | OOU_Y34scaffold00608g93 | 0.68 | yes | 146 |
| 2 | ThiJ/PfpI family protein | OOU_Y34scaffold00125g2 | 0.27 | yes | 248 |
| 3 | Acid phosphatase | OOU_Y34scaffold00522g54 | 0.74 | yes | 464 |
| 4 | Uncharacterized protein | OOU_Y34scaffold00601g6 | 0.94 | yes | 149 |
| 5 | Uncharacterized protein | OOU_Y34scaffold00589g14 | 0.77 | yes | 137 |
| 6 | Uncharacterized protein | OOU_Y34scaffold00073g12 | 0.46 | yes | 189 |
| 7 | Uncharacterized protein | OOU_Y34scaffold00192g52 | 0.44 | yes | 390 |
| 8 | Uncharacterized protein | OOU_Y34scaffold00734g10 | 0.79 | yes | 234 |
| 9 | Uncharacterized protein | OOU_Y34scaffold00522g24 | 0.56 | yes | 207 |
| 10 | Uncharacterized protein | OOU_Y34scaffold00246g4 | 0.221 | yes | 210 |
| 11 | Uncharacterized protein | OOU_Y34scaffold00526g23 | 0.771 | yes | 244 |
| 12 | Uncharacterized protein | OOU_Y34scaffold00696g3 | 0.51 | yes | 274 |
| 13 | Aspergillopepsin-F | OOU_Y34scaffold00458g41 | 0.38 | yes | 407 |
| 14 | Uncharacterized protein | OOU_Y34scaffold00071g64 | 0.22 | yes | 267 |
| 15 | Uncharacterized protein | OOU_Y34scaffold00726g55 | 0.03 | yes | 208 |
| 16 | Uncharacterized protein | OOU_Y34scaffold01028g4 | 0.47 | yes | 183 |
| 17 | Uncharacterized protein | OOU_Y34scaffold00666g122 | 1.11 | yes | 142 |
| 18 | Aspergillopepsin-F | OOU_Y34scaffold00547g6 | 0.045 | yes | 430 |
| 19 | Uncharacterized protein | OOU_Y34scaffold01180g4 | 0.66 | yes | 199 |
| 21 | [AVR-Pia](https://www.uniprot.org/uniprot/R9RX08) | OOU_Y34scaffold01050g2 | 0.43 | yes | 85 |
| 22 | Candidapepsin-3 | OOU_Y34scaffold00559g2 | 0.24 | yes | 474 |
| 23 | Surface protein 1 | OOU_Y34scaffold00799g5 | 0.37 | yes | 146 |
| 24 | Uncharacterized protein | OOU_Y34scaffold00616g1 | 0.27 | yes | 141 |
| 25 | Uncharacterized protein | OOU_Y34scaffold00624g82 | 0.45 | yes | 197 |
| 26 | Uncharacterized protein | OOU_Y34scaffold00636g9 | 0.33 | yes | 301 |
| 27 | Uncharacterized protein | OOU_Y34scaffold00432g6 | 0.15 | yes | 232 |
| 28 | Surface protein 1 | OOU_Y34scaffold00050g13 | 0.08 | yes | 134 |
| 29 | Uncharacterized protein | OOU_Y34scaffold00412g18 | 0.35 | yes | 268 |
| 30 | Bys1 family protein | OOU_Y34scaffold00037g32 | 1.14 | yes | 161 |
| 31 | Uncharacterized protein | OOU_Y34scaffold00095g19 | 0.68 | yes | 247 |
| 32 | Uncharacterized protein | OOU_Y34scaffold00325g47 | 0.78 | yes | 282 |
| 33 | Uncharacterized protein | OOU_Y34scaffold00217g22 | 0.46 | yes | 224 |
| 34 | Uncharacterized protein | OOU_Y34scaffold00533g27 | 0.6 | yes | 156 |
| 35 | Uncharacterized protein | OOU_Y34scaffold00034g2 | 0.3 | yes | 276 |
| 36 | Candidapepsin-8 | OOU_Y34scaffold00233g5 | 0.73 | yes | 476 |
| 37 | Uncharacterized protein | OOU_Y34scaffold00174g86 | 0.23 | yes | 240 |
| 38 | Peptide hydrolase | OOU_Y34scaffold00182g24 | 0.72 | yes | 389 |
| 39 | Uncharacterized protein | OOU_Y34scaffold00548g78 | 0.45 | yes | 225 |
| 40 | Uncharacterized protein | OOU_Y34scaffold00522g67 | 0.52 | yes | 328 |
| 41 | WSC domain-containing protein | OOU_Y34scaffold00044g7 | 0.69 | yes | 384 |
| 42 | Metalloprotease 1 | OOU_Y34scaffold00910g2 | 0.27 | yes | 288 |
| 43 | Peptide hydrolase | OOU_Y34scaffold01075g7 | 0.37 | yes | 494 |
| 44 | Uncharacterized protein | OOU_Y34scaffold01073g23 | 0.77 | yes | 118 |
| 45 | Uncharacterized protein | OOU_Y34scaffold00555g10 | 0.22 | yes | 202 |
| 46 | Uncharacterized protein | OOU_Y34scaffold00325g24 | 0.37 | yes | 145 |
| 47 | Uncharacterized protein | OOU_Y34scaffold00628g21 | 0.05 | yes | 110 |
| 48 | Peptide hydrolase | OOU_Y34scaffold01003g39 | 0.354 | yes | 506 |
| 49 | Uncharacterized protein | OOU_Y34scaffold00664g1 | 0.54 | yes | 152 |
| 50 | Endoprotease | OOU_Y34scaffold00726g88 | 0.99 | yes | 542 |
| 51 | Uncharacterized protein | OOU_Y34scaffold00692g41 | 0.22 | yes | 212 |
| 52 | Uncharacterized protein | OOU_Y34scaffold00174g47 | 0.85 | yes | 327 |
| 53 | Uncharacterized protein | OOU_Y34scaffold00781g1 | 0.45 | yes | 247 |
| 54 | Uncharacterized protein | OOU_Y34scaffold00694g5 | 0.56 | yes | 93 |
| 55 | Uncharacterized protein | OOU_Y34scaffold00649g10 | 0.57 | yes | 283 |
| 56 | Lipase 2 | OOU_Y34scaffold00712g22 | 0.25 | yes | 662 |
| 57 | Uncharacterized protein | OOU_Y34scaffold00069g2 | 0.81 | no | 562 |
| 58 | Beta-glucuronidase | OOU_Y34scaffold00492g37 | 0.78 | yes | 482 |
| 59 | Uncharacterized protein | OOU_Y34scaffold00370g15 | 0.28 | yes | 368 |
| 60 | Uncharacterized protein | OOU_Y34scaffold00542g72 | 0.22 | yes | 477 |
| 61 | Ribonuclease Trv | OOU_Y34scaffold00589g11 | 1.57 | no | 318 |
| 62 | Uncharacterized protein | OOU_Y34scaffold00500g4 | 0.52 | no | 438 |
| 63 | Minor extracellular protease vpr | OOU_Y34scaffold00547g5 | 1.43 | yes | 906 |
| 64 | Cytochrome c | OOU_Y34scaffold00533g52 | 0.7 | no | 108 |
| 65 | Repressible acid phosphatase | OOU_Y34scaffold00283g91 | 1.52 | yes | 437 |
| 66 | Mannan endo-1,6-alpha-mannosidase | OOU_Y34scaffold00707g7 | 1.85 | no | 421 |
| 67 | Superoxide dismutase | OOU_Y34scaffold00127g3 | 0.86 | no | 203 |
| 68 | Endo-beta-1,3-glucanase | OOU_Y34scaffold00334g13 | 5.64 | no | 769 |
| 69 | Uncharacterized protein | OOU_Y34scaffold00214g10 | 0.79 | yes | 494 |
| 70 | Lipase OS | OOU_Y34scaffold00224g3 | 0.63 | yes | 400 |
| 71 | Uncharacterized protein | OOU_Y34scaffold01005g5 | 0.2 | no | 541 |
| 72 | D-tyrosyl-tRNA(Tyr) deacylase | OOU_Y34scaffold00094g14 | 0.86 | no | 137 |
| 73 | Peptidyl-prolyl cis-trans isomerase | OOU_Y34scaffold00189g6 | 0.23 | no | 165 |
| 74 | Uncharacterized protein | OOU_Y34scaffold00726g102 | 1.59 | yes | 505 |
| 75 | Ulilysin | OOU_Y34scaffold00492g54 | 0.1 | no | 280 |
| 76 | Peroxidase | OOU_Y34scaffold00050g12 | 0.48 | yes | 474 |
| 77 | Aspartate aminotransferase | OOU_Y34scaffold00255g5 | 0.46 | no | 457 |
| 78 | Uncharacterized protein | OOU_Y34scaffold00462g24 | 2.12 | no | 345 |
| 79 | Transaldolase | OOU_Y34scaffold00666g190 | 0.55 | no | 324 |
| 80 | Podosporapepsin | OOU_Y34scaffold00533g6 | 0.44 | yes | 417 |
| 81 | Endoglucanase II | OOU_Y34scaffold01003g18 | 0.75 | yes | 258 |
| 82 | Secretory lipase family protein | OOU_Y34scaffold00414g54 | 0.45 | yes | 471 |
| 83 | Peroxiredoxin type-2 | OOU_Y34scaffold00666g77 | 0.82 | no | 168 |
| 84 | Uncharacterized protein | OOU_Y34scaffold00211g7 | 0.41 | yes | 302 |
| 85 | Uncharacterized protein | OOU_Y34scaffold00267g16 | 0.43 | no | 404 |
| 86 | Uncharacterized protein | OOU_Y34scaffold00141g7 | 0.03 | yes | 830 |
| 87 | Glucan 1,3-beta-glucosidase | OOU_Y34scaffold00099g20 | 1.56 | yes | 769 |
| 88 | ADP-ribosylation factor 1 | OOU_Y34scaffold00744g40 | 0.95 | no | 183 |
| 89 | Nucleoside diphosphate kinase | OOU_Y34scaffold00685g12 | 0.75 | no | 241 |
| 90 | Uncharacterized protein | OOU_Y34scaffold00598g3 | 0.73 | no | 124 |
| 91 | Bacterial hemoglobin | OOU_Y34scaffold00793g24 | 0.96 | no | 447 |
| 92 | Cofilin | OOU_Y34scaffold00155g33 | 0.74 | no | 152 |
| 93 | Uncharacterized protein | OOU_Y34scaffold00995g21 | 0.26 | yes | 184 |
| 94 | Uncharacterized protein | OOU_Y34scaffold00799g8 | 0.38 | No | 192 |
| 95 | Uncharacterized protein | OOU_Y34scaffold00686g16 | 0.42 | no | 86 |
| 96 | Uncharacterized protein | OOU_Y34scaffold00666g106 | 3.96 | no | 125 |
| 97 | Uncharacterized protein | OOU_Y34scaffold00666g192 | 1.95 | no | 135 |
| 98 | Uncharacterized protein | OOU_Y34scaffold00528g86 | 0.52 | yes | 86 |
| 99 | Integral membrane protein | OOU_Y34scaffold00283g12 | 0.49 | no | 164 |
| 100 | Uncharacterized protein | OOU_Y34scaffold00126g56 | 0.26 | yes | 146 |
| 101 | Uncharacterized protein | OOU_Y34scaffold00094g25 | 0.35 | no | 184 |
| 102 | Uncharacterized protein | OOU_Y34scaffold00071g56 | 0.52 | yes | 173 |
| 103 | Uncharacterized protein | OOU_Y34scaffold00045g4 | 2.69 | no | 186 |
| 104 | Uncharacterized protein | OOU_Y34scaffold00090g17 | 0.65 | no | 205 |
