# Supplementary material for: Magnaporthe oryzae fimbrin organizes actin networks in the hyphal tip during polar growth and pathogenesis
Source: PLoS Pathog. 2020 Mar 16;16(3):e1008437. doi: 10.1371/journal.ppat.1008437 (PMC7098657; doi:10.1371/journal.ppat.1008437)
Supplement: S2 Table — (DOC) [file ppat.1008437.s013.doc]

**Primers used in this study**

The nucleotides highlighted in red indicate the enzyme site for construction.

| CAAATGGGTCGCGGATCCATGAACGTTCTCA AACTTC | F-MoFim1 (PET28a) |
| --- | --- |
| GAGTGCGGCCGCAAGCTTCTAAGCCATCTTTTCATGC | R-MoFim1 (PET28a) |
| CAAATGGGTCGCGGATCCATGGTGAGCAAGGGCGAG | F-GFP (PET28a) |
| GAGTGCGGCCGCAAGCTTCTACTTGTACAGCTCGTCC | R-GFP (PET28a) |
| CAAATGGGTCGCGGATCCATGAACGTTCTCA AACTTC | F-MoFim1  (PET28a-GFP) |
| CCTTGCTCACCATGGATCCAGCCATCTTTTCATGCGT | R-MoFim1  (PET28a-GFP) |
| CAAATGGGTCGCGGATCCATGAACGTTCTCA AACTTC | 1. EF   (PET28a-GFP) |
| CCTTGCTCACCATGGATCCCTCGTTGATGGTATGCG | 1. EF   (PET28a-GFP) |
| CAAATGGGTCGCGGATCCATGGATGAGCGCACAGAG | F-ABD1  (PET28a-GFP) |
| CCTTGCTCACCATGGATCCAGGGTGTGTGTTGAACAG | R-ABD1  (PET28a-GFP) |
| CAAATGGGTCGCGGATCCATGCGTGTCTTTACTCTGTG | F-ABD2  (PET28a-GFP) |
| CCTTGCTCACCATGGATCCCGCCATCAGGGAACCAATA | R-ABD2  (PET28a-GFP) |
| CAAATGGGTCGCGGATCCATGAACGTTCTCA AACTTC | F-EF-ABD1  (PET28a-GFP) |
| CCTTGCTCACCATGGATCCAGGGTGTGTGTTGAACAG | R-EF-ABD1  (PET28a-GFP) |
| CAAATGGGTCGCGGATCCATGAACGTTCTCA AACTTC | F-EF-ABD2-1  (PET28a-GFP) |
| GCCACAGAGTAAAGACACGCTCGTTGATGGTATGCGT | R-EF-ABD2-1  (PET28a-GFP) |
| CACGCATACCATCAACGAGCGTGTCTTTACTCTGTGGC | F-EF-ABD2-2  (PET28a-GFP) |
| CCTTGCTCACCATGGATCCCGCCATCAGGGAACCAATA | R-EF-ABD2  (PET28a-GFP) |
| GTACCAGATTACGCTCATATGATGAACGTTCTCAAACTT | F-EF (pGADT7) |
| GCTCGAGCTCGATGGATCCTTACTCGTTGATGGTATGC | R-EF (pGADT7) |
| GCCATGGAGGCCGAATTCATGAACGTTCTCAAACTT | F-EF (pGBKT7) |
| GCTGCAGGTCGACGGATCCTTAACTCGTTGATGGTATG | R-EF (pGBKT7) |
| AACGACGGCCAGTGCCAAGCTTCATGGCCCAAGCTTTGT | F-NP-MoFim1  (PBN-GFP) |
| CGCCCTTGCTCACCATCCCGGGAGCCATCTTTTCATGCGT | R-NP-MoFim1  (PBN-GFP) |
| AACGACGGCCAGTGCCAAGCTTCGTCTGCTTTGCTCGTC | F-NP-Sep3  (PBN-GFP) |
| CGCCCTTGCTCACCATCCCGGGACGGAGTGAGAAACC | R-NP-Sep3  (PBN-GFP) |
| AACGACGGCCAGTGCCAAGCTTGTGTATAGATTCGCTACT | F-NP-Myosin5  (PBN-GFP) |
| CGCCCTTGCTCACCATCCCGGGGTCCTGGGCTGGACAGG | R-NP-Myosin5  (PBN-GFP) |
| AACGACGGCCAGTGCCAAGCTTCAGTTGTGCAGGTTGGTTG | F-NP-SNC1  (PBN-GFP) |
| CGCCCTTGCTCACCATCCCGGGGTTGCCCTTGAAGTGGAAAAC | R-NP-SNC1  (PBN-GFP) |
| AACGACGGCCAGTGCCAAGCTTCACCCTTTGACCAATGC | F-NP-Sec9  (PBN-GFP) |
| CGCCCTTGCTCACCATCCCGGGACCCTTCTTGTAGATGCG | R-NP-Sec9  (PBN-GFP) |
| AACGACGGCCAGTGCCAAGCTTGAGGCTTGTGCAGGGCC | F-NP-Exo84  (PBN-GFP) |
| CGCCCTTGCTCACCATCCCGGGAGAGAGGCCGAGGCCAAG | R-NP-Exo84  (PBN-GFP) |
| TATGACCATGATTACGAATTCCATGGCCCAAGCTTTGT | F-NP-MoFim1  (PBN-mCherry) |
| GCTCACCATGGATCCGGTACCAGCCATCTTTTCATGCGT | R-NP-MoFim1  (PBN-mCherry) |
| TATGACCATGATTACGAATTCCATGGCCCAAGCTTTGT | F-NP-EF  (PBN-mCherry) |
| GCTCACCATGGATCCGGTACCCTCGTTGATGGTATGCG | R-NP-EF  (PBN-mCherry) |
| TATGACCATGATTACGAATTCCATGGCCCAAGCTTTGT | F-NP-ABD1  (PBN-mCherry) |
| GCTCACCATGGATCCGGTACCAGGGTGTGTGTTGAAC | R-NP-ABD1  (PBN-mCherry) |
| TATGACCATGATTACGAATTCCATGGCCCAAGCTTTGT | F-NP-ABD2  (PBN-mCherry) |
| GCTCACCATGGATCCGGTACCCGCCATCAGGGAACCT | R-NP-ABD2  (PBN-mCherry) |
| TATGACCATGATTACGAATTCCATGGCCCAAGCTTTGT | F-EF-ABD1  (PBN-mCherry) |
| GCTCACCATGGATCCGGTACCAGGGTGTGTGTTGAAC | R-EF-ABD1  (PBN-mCherry) |
| TATGACCATGATTACGAATTCCATGGCCCAAGCTTTGT | F-EF-ABD2-1  (PBN-mCherry) |
| GCCACAGAGTAAAGACACGCTCGTTGATGGTATGCGT | R-EF-ABD2-1  (PBN-mCherry) |
| CACGCATACCATCAACGAGCGTGTCTTTACTCTGTGGC | F-EF-ABD2-2  (PBN-mCherry) |
| GCTCACCATGGATCCGGTACCCGCCATCAGGGAACCT | R-EF-ABD2-2  (PBN-mCherry) |
| GGTGGCGGCCGCTCTAGACGGTAGCACCCGTTAACGCTG | F-MoFim1-1  (PGKO-HPH) |
| CAAAAATGCTCCTTCAATCTAGAGACTGTTATTTGGAGGATC | R-MoFim1-1  (PGKO-HPH) |
| GGGTTCGCAAAGATAAAAGCTTGCTTGTGGTGGGTCCAG | F-MoFim1-2  (PGKO-HPH) |
| GGTCGACGGTATCGATAAGCTTGGTGTGTGAGTGAGAGG | R-MoFim1-2  (PGKO-HPH) |
| CAGCGTGCCCGACACCATCG | F-test1 |
| CCAGCGCAGCAGGATCTGCT | R-test1 |
| CATGGCCCAAGCTTTGTTTG | F-test2 |
| CACAAGTTATCGTGCACCAA | R-test2 |
| GATACAGCTCATCTGCAATGC | F-test3 |
| GCAGATGGATGGTATGTGAG | R-test3 |
| CATGGCCCAAGCTTTGTTTG | F-test4 |
| GCAGATGGATGGTATGTGAG | R-test4 |
| CACGGGGGACTCTAGAGGATCCCCTACGGCGTGCAGTGCTTC | F-GFP-1 (RNAi) |
| CGCGAAGCGGGTAGATCCCGGGGCGGTCACGAACTCCAGCAG | R-GFP-1 (RNAi) |
| GAAAGGGATCTTCACTCGGTCGACGCGGTCACGAACTCC | F-GFP-2 (RNAi) |
| GAACGATCGGGGAAATTCGAGCTCCCTACGGCGTGCAGTG | R-GFP-2 (RNAi) |
| CACGGGGGACTCTAGAGGATCCATGAACGTTCTCAAAC | F-MoFim1-F1-1  (RNAi in plant) |
| CGCGAAGCGGGTAGATCCCGGGGAGTCCAGATCGACTTC | R-MoFim1-F1-1  (RNAi in plant) |
| GAAAGGGATCTTCACTCGGTCGACGAGTCCAGATCGACTTC | F-MoFim1-F1-2  (RNAi in plant) |
| GAACGATCGGGGAAATTCGAGCTCATGAACGTTCTCAAAC | R-MoFim1-F1-2  (RNAi in plant) |
| ATACATCACCGTCAAACCGAGCTCATGAACGTTCTCAAAC | F-MoFim1-F1-1  (RNAi in fungi) |
| GACGCGAAGCGGGTAGATGAGCTCGAGTCCAGATCGACTTC | R-MoFim1-F1-1  (RNAi in fungi) |
| GAAAGGGATCTTCACTCGGGATCCGAGTCCAGATCGACTTC | F-MoFim1-F1-2  (RNAi in fungi) |
| GCCAAATGTTTGAACGATCGGATCCATGAACGTTCTCAAAC | R-MoFim1-F1-2  (RNAi in fungi) |
| ATACATCACCGTCAAACCGAGCTCCCTCTCGCCCCATCAGG | F-MoFim1-F2-1  (RNAi in fungi) |
| GACGCGAAGCGGGTAGATGAGCTCGGTGTCGGTAGGGAAG | R-MoFim1-F2-1  (RNAi in fungi) |
| GAAAGGGATCTTCACTCGGGATCCGGTGTCGGTAGGGAAG | F-MoFim1-F2-2  (RNAi in fungi) |
| GCCAAATGTTTGAACGATCGGATCCCCTCTCGCCCCATCAGG | R-MoFim1-F2-2  (RNAi in fungi) |
| ATACATCACCGTCAAACCGAGCTCGGAGCAGATATAACCGA | F-MoFim1-F3-1  (RNAi in fungi) |
| GACGCGAAGCGGGTAGATGAGCTCGGTCCTTGAATGACCG | R-MoFim1-F3-1  (RNAi in fungi) |
| GAAAGGGATCTTCACTCGGGATCCGGTCCTTGAATGACCG | F-MoFim1-F3-2  (RNAi in fungi) |
| GCCAAATGTTTGAACGATCGGATCCGGAGCAGATATAACCGA | R-MoFim1-F3-2  (RNAi in fungi) |
| CATTGTCCACCGCAAGTG | F-MoACT1  (qRT-PCR) |
| GCCGATATTGCTGCGAGT | R-MoACT1  (qRT-PCR) |
| CGCCATGCCCGAAGGCTAC | F-GFP (qRT-PCR) |
| TGATATAGACGTTGTGGCTG | R-GFP (qRT-PCR) |
| CGATGTCGTGCGCCAGGCC | F-MoFim1 (qRT-PCR) |
| GCAAGAACGGCGTTGATGT | R-MoFim1 (qRT-PCR) |
