# Supplementary figures and images for: Correction: Magnaporthe oryzae fimbrin organizes actin networks in the hyphal tip during polar growth and pathogenesis
Source: PLoS Pathog. 2024 May 6;20(5):e1012210. doi: 10.1371/journal.ppat.1012210 (PMC11073713; doi:10.1371/journal.ppat.1012210)

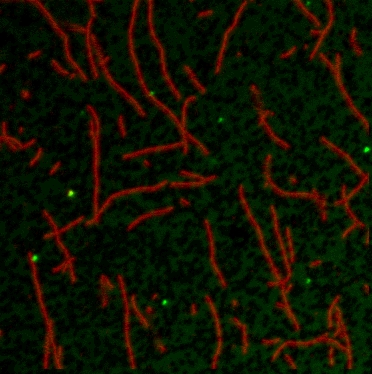

Supplement: S1 File — (ZIP) [file ppat.1012210.s001.zip › Figure 5B best fit mode MoFim1-GFP/Figure 5B-Image Export-02_c1+2.jpg]

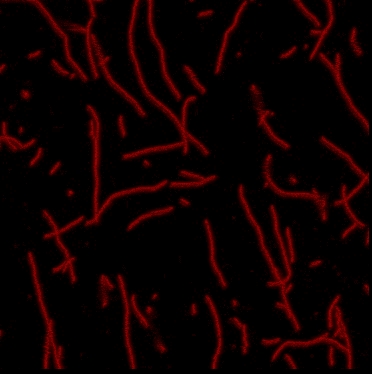

Supplement: S1 File — (ZIP) [file ppat.1012210.s001.zip › Figure 5B best fit mode MoFim1-GFP/Figure 5B-Image Export-02_c1.jpg]

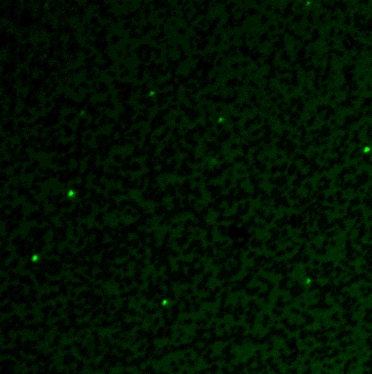

Supplement: S1 File — (ZIP) [file ppat.1012210.s001.zip › Figure 5B best fit mode MoFim1-GFP/Figure 5B-Image Export-02_c2.jpg]

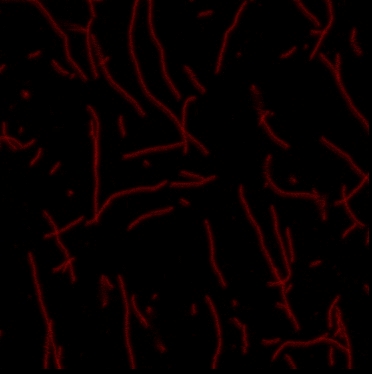

Supplement: S1 File — (ZIP) [file ppat.1012210.s001.zip › Figure 5B default mode MoFim1-GFP/Figure 5B-Image Export-03_c1+2.jpg]

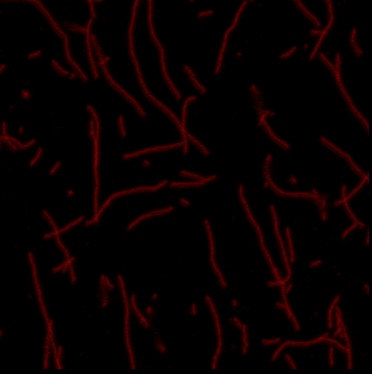

Supplement: S1 File — (ZIP) [file ppat.1012210.s001.zip › Figure 5B default mode MoFim1-GFP/Figure 5B-Image Export-03_c1.jpg]

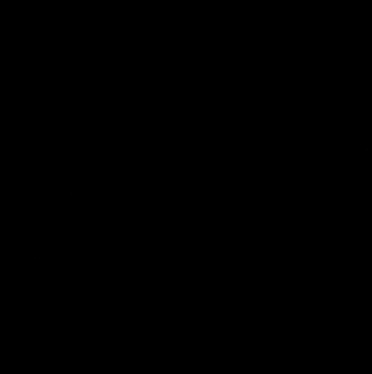

Supplement: S1 File — (ZIP) [file ppat.1012210.s001.zip › Figure 5B default mode MoFim1-GFP/Figure 5B-Image Export-03_c2.jpg]

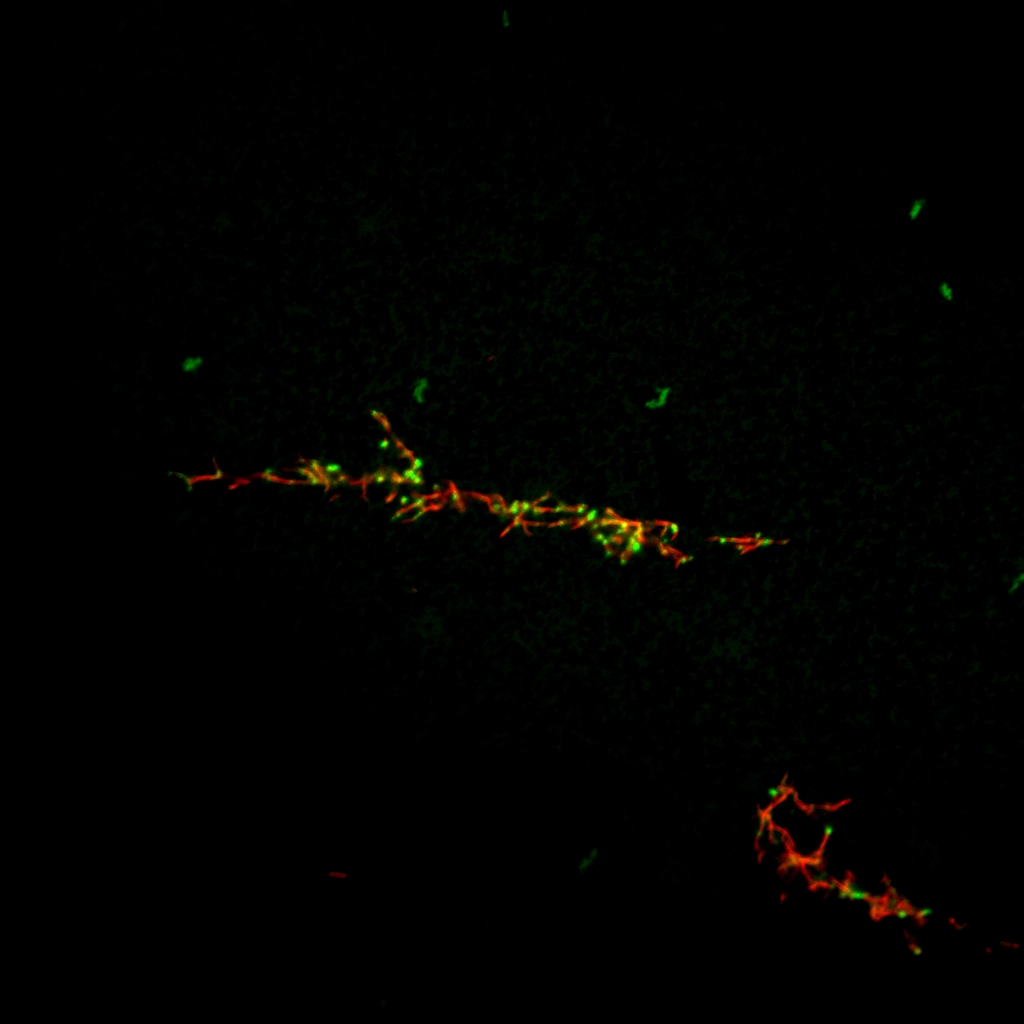

Supplement: S1 File — (ZIP) [file ppat.1012210.s001.zip › Figure 5C best fit mode MoFim1-GFP/MoFim1-GFP_c1+2.jpg]

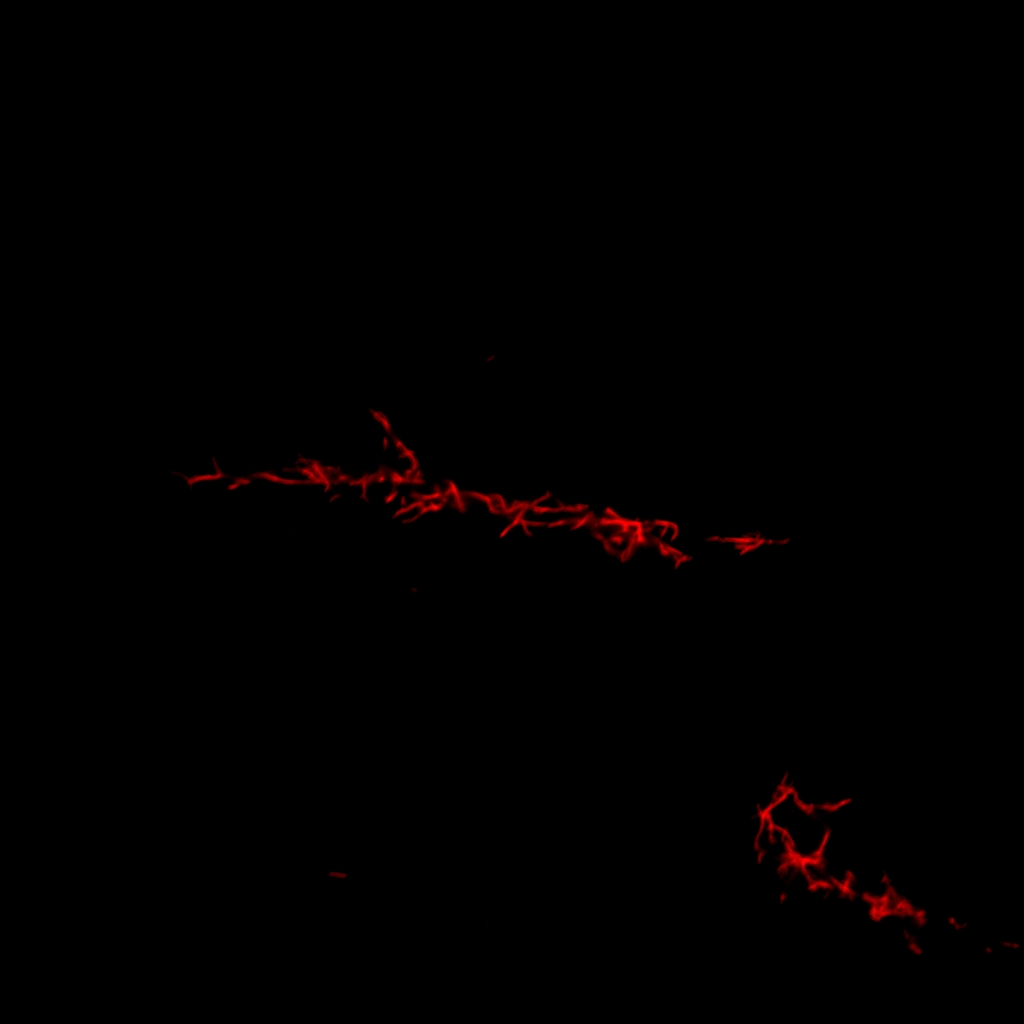

Supplement: S1 File — (ZIP) [file ppat.1012210.s001.zip › Figure 5C best fit mode MoFim1-GFP/MoFim1-GFP_c1.jpg]

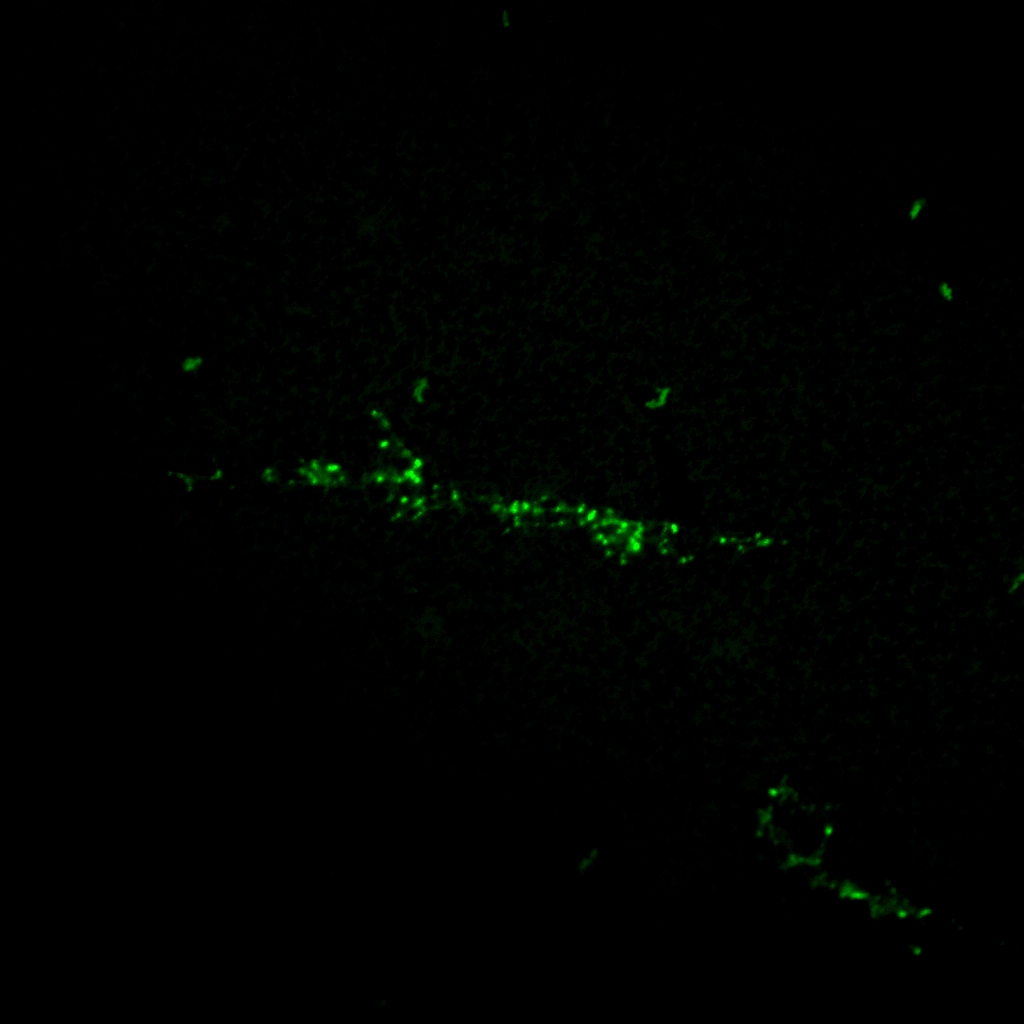

Supplement: S1 File — (ZIP) [file ppat.1012210.s001.zip › Figure 5C best fit mode MoFim1-GFP/MoFim1-GFP_c2.jpg]

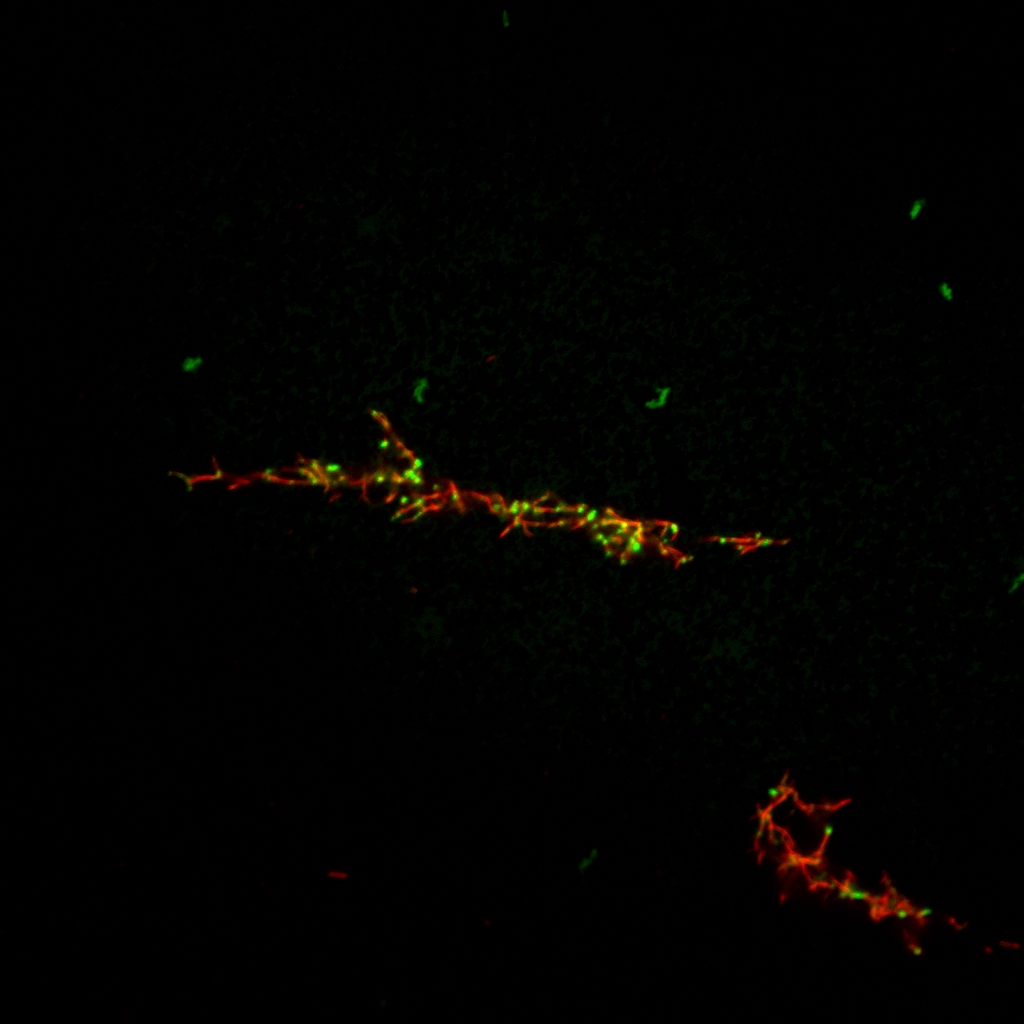

Supplement: S1 File — (ZIP) [file ppat.1012210.s001.zip › Figure 5C default mode MoFim1-GFP/MoFim1-GFP_c1+2.jpg]

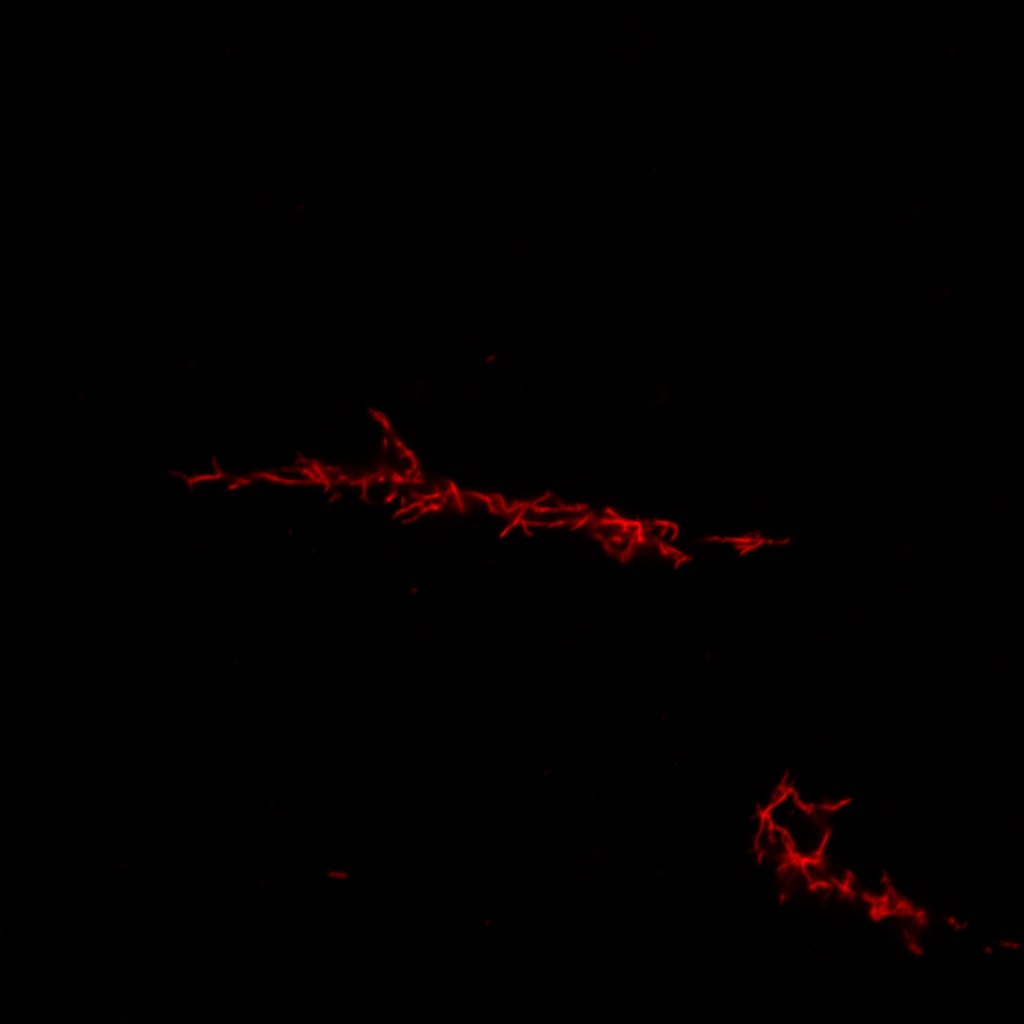

Supplement: S1 File — (ZIP) [file ppat.1012210.s001.zip › Figure 5C default mode MoFim1-GFP/MoFim1-GFP_c1.jpg]

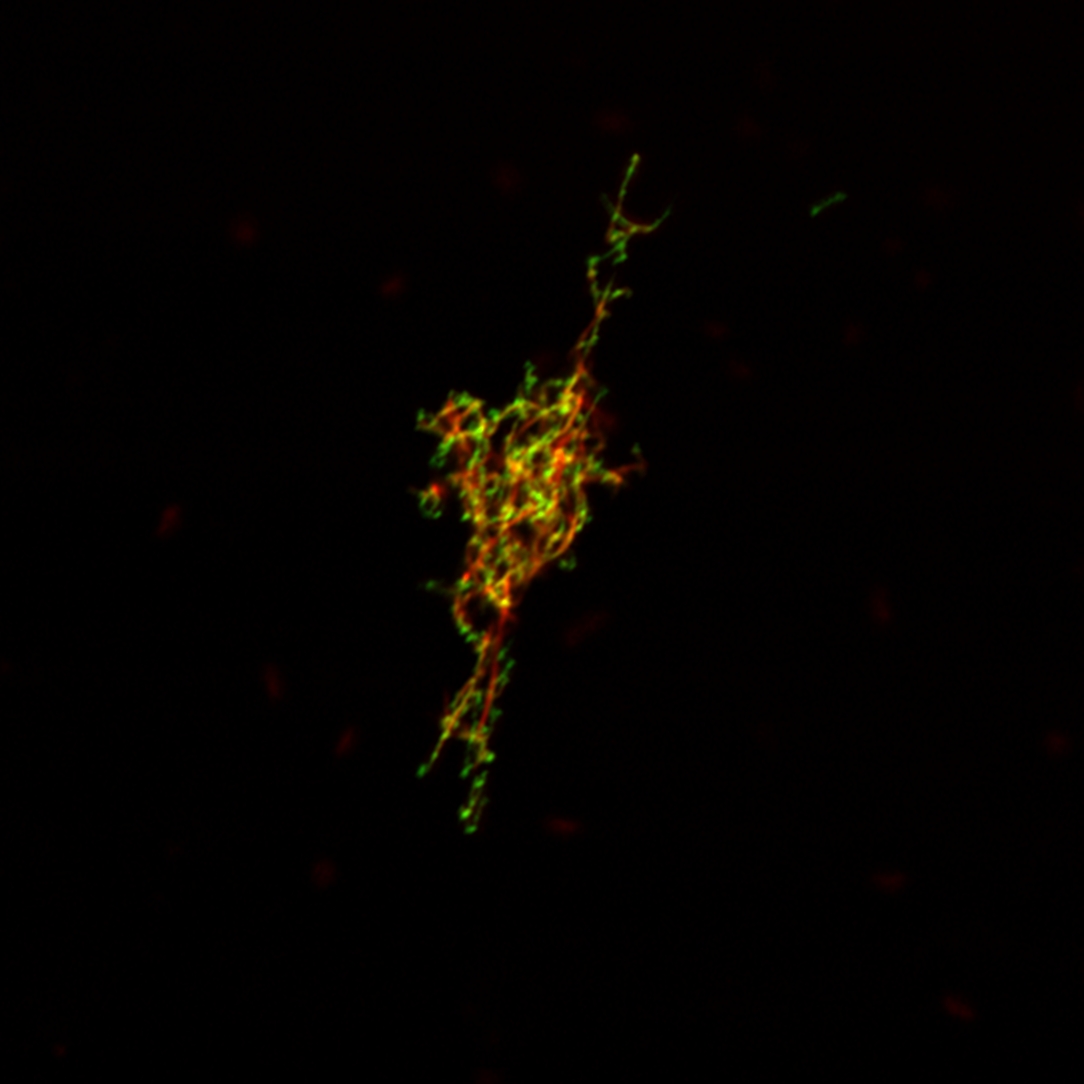

Supplement: S1 File — (ZIP) [file ppat.1012210.s001.zip › Figure 5D best fit mode MoFim1-GFP+Ca2+/MoFim1-GFP+Ca2+_c1+2.jpg]

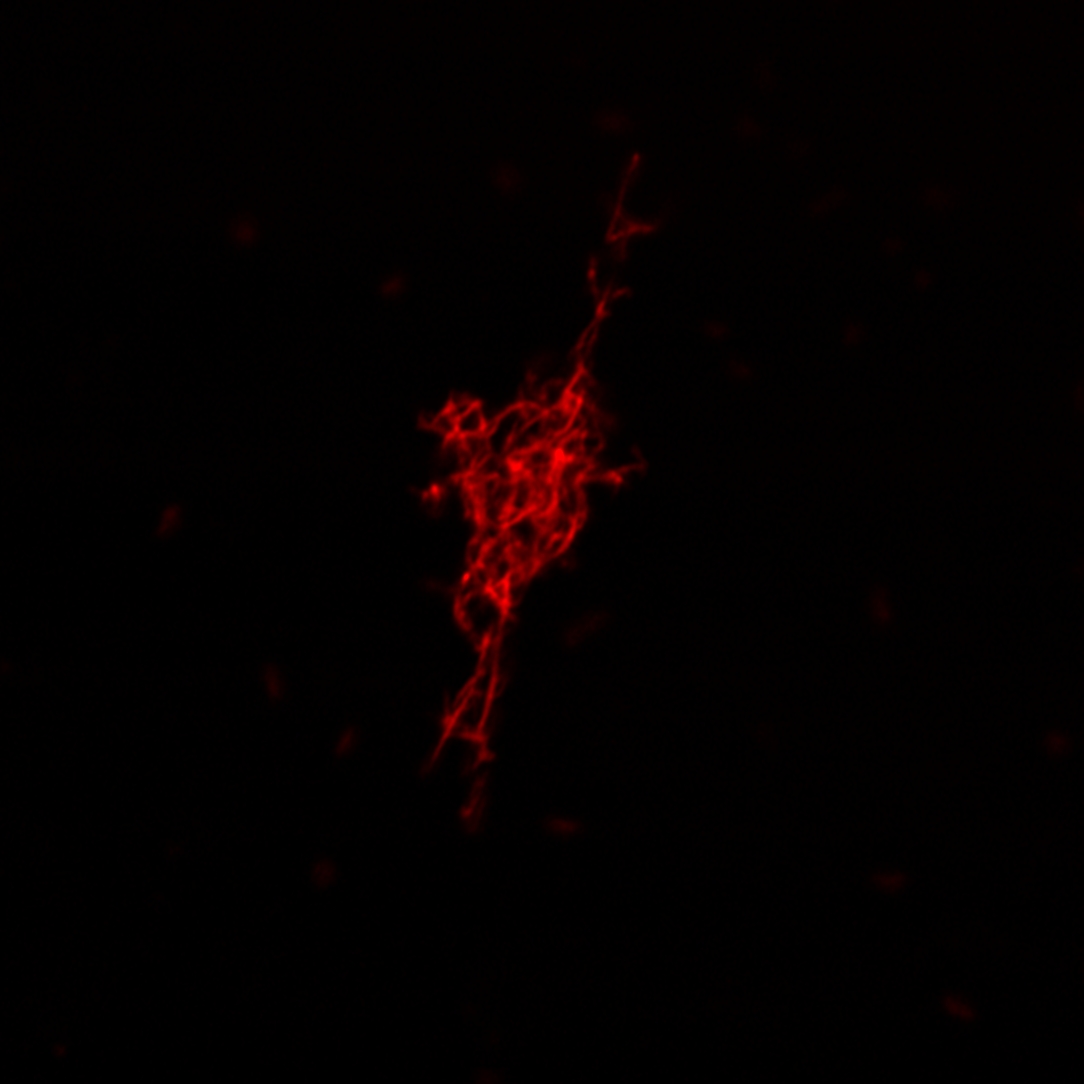

Supplement: S1 File — (ZIP) [file ppat.1012210.s001.zip › Figure 5D best fit mode MoFim1-GFP+Ca2+/MoFim1-GFP+Ca2+_c1.jpg]

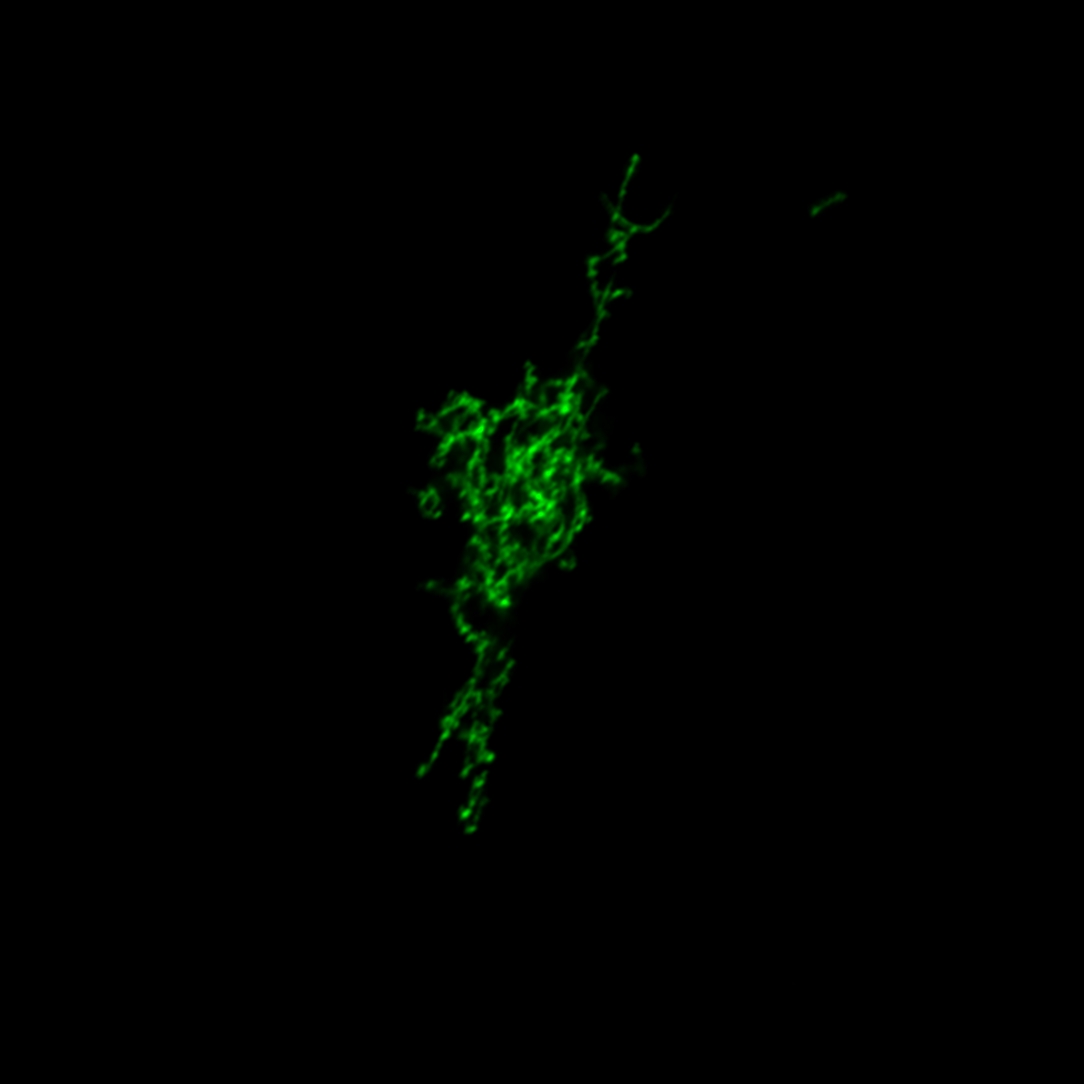

Supplement: S1 File — (ZIP) [file ppat.1012210.s001.zip › Figure 5D best fit mode MoFim1-GFP+Ca2+/MoFim1-GFP+Ca2+_c2.jpg]

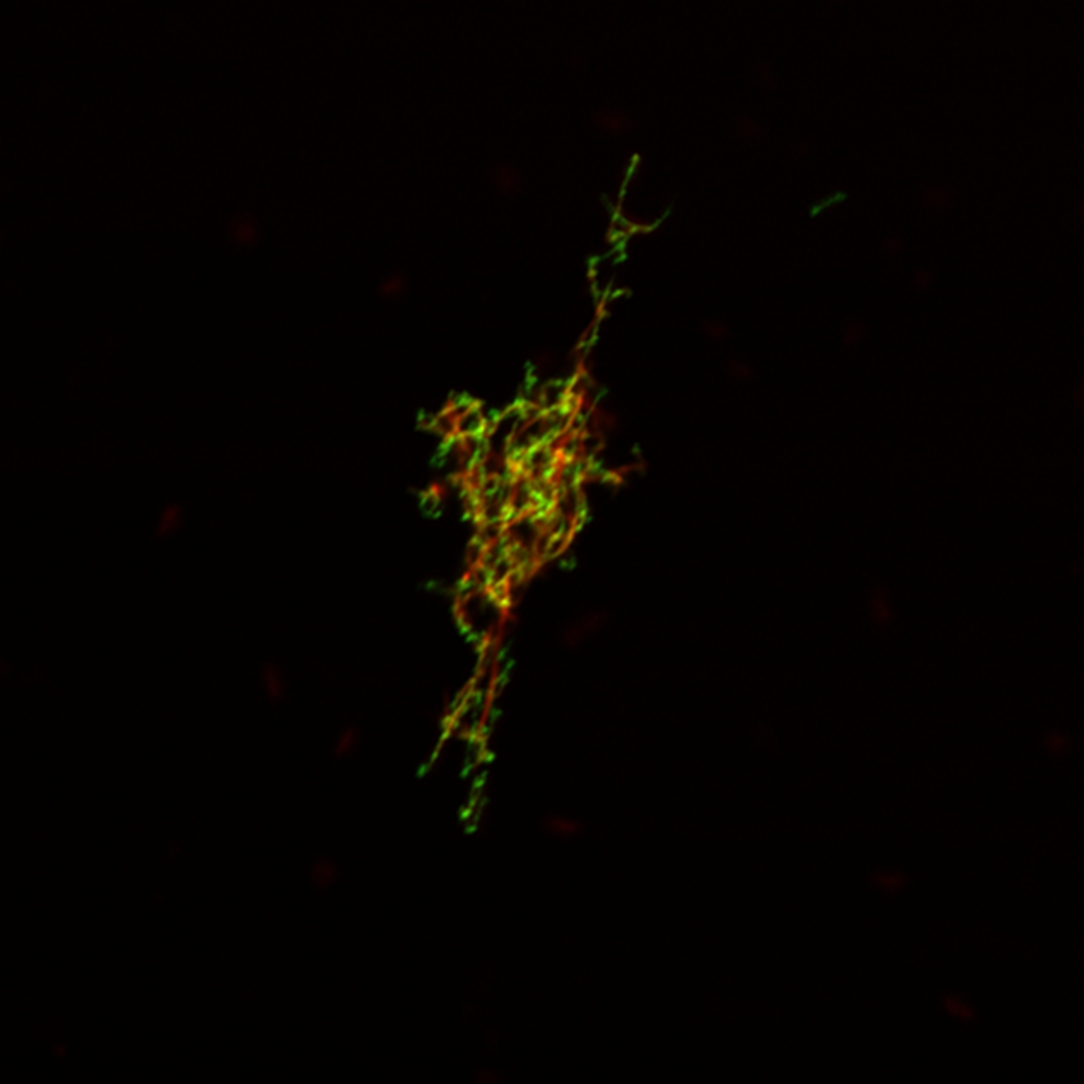

Supplement: S1 File — (ZIP) [file ppat.1012210.s001.zip › Figure 5D default mode MoFim1-GFP+Ca2+/MoFim1-GFP+Ca2+_c1+2.jpg]

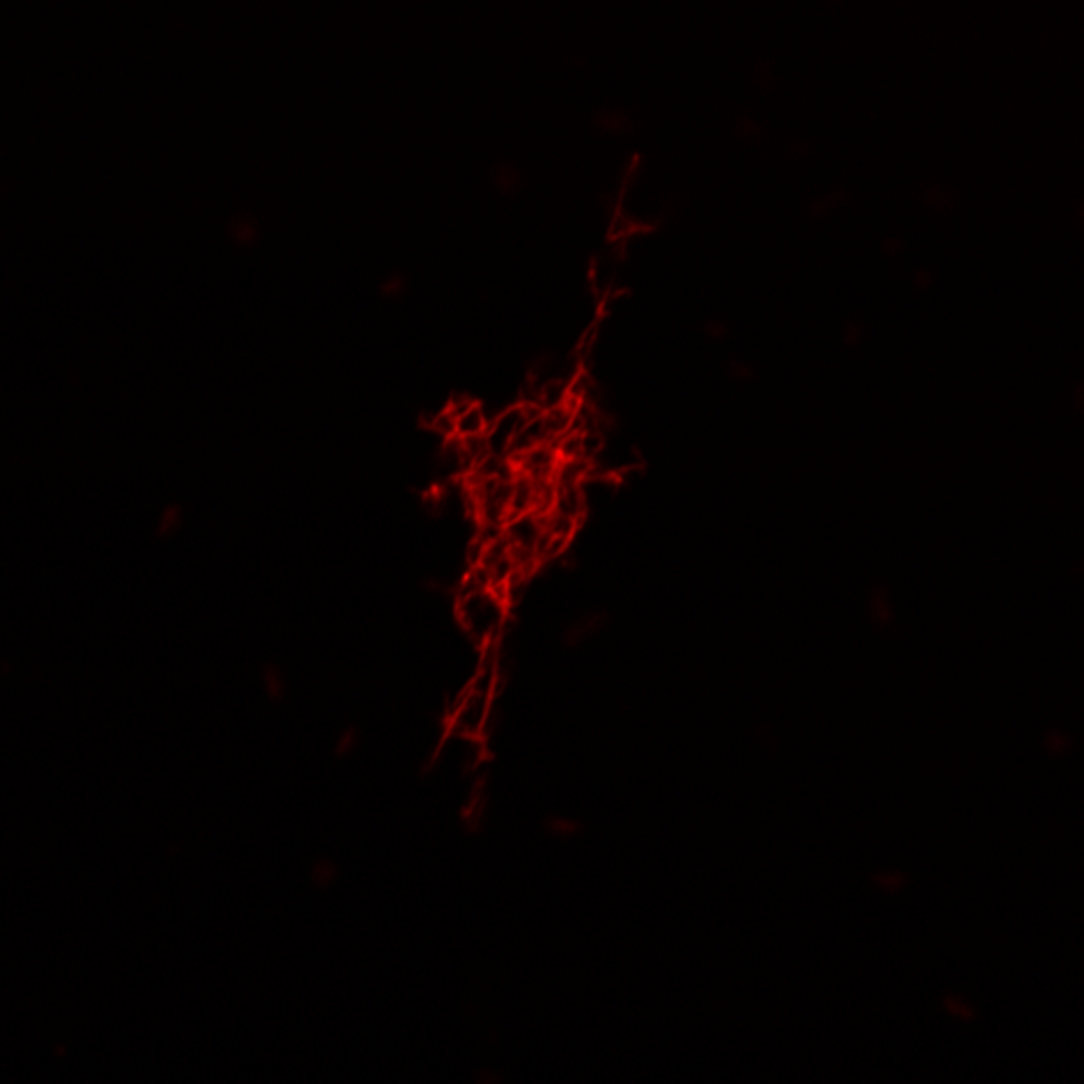

Supplement: S1 File — (ZIP) [file ppat.1012210.s001.zip › Figure 5D default mode MoFim1-GFP+Ca2+/MoFim1-GFP+Ca2+_c1.jpg]

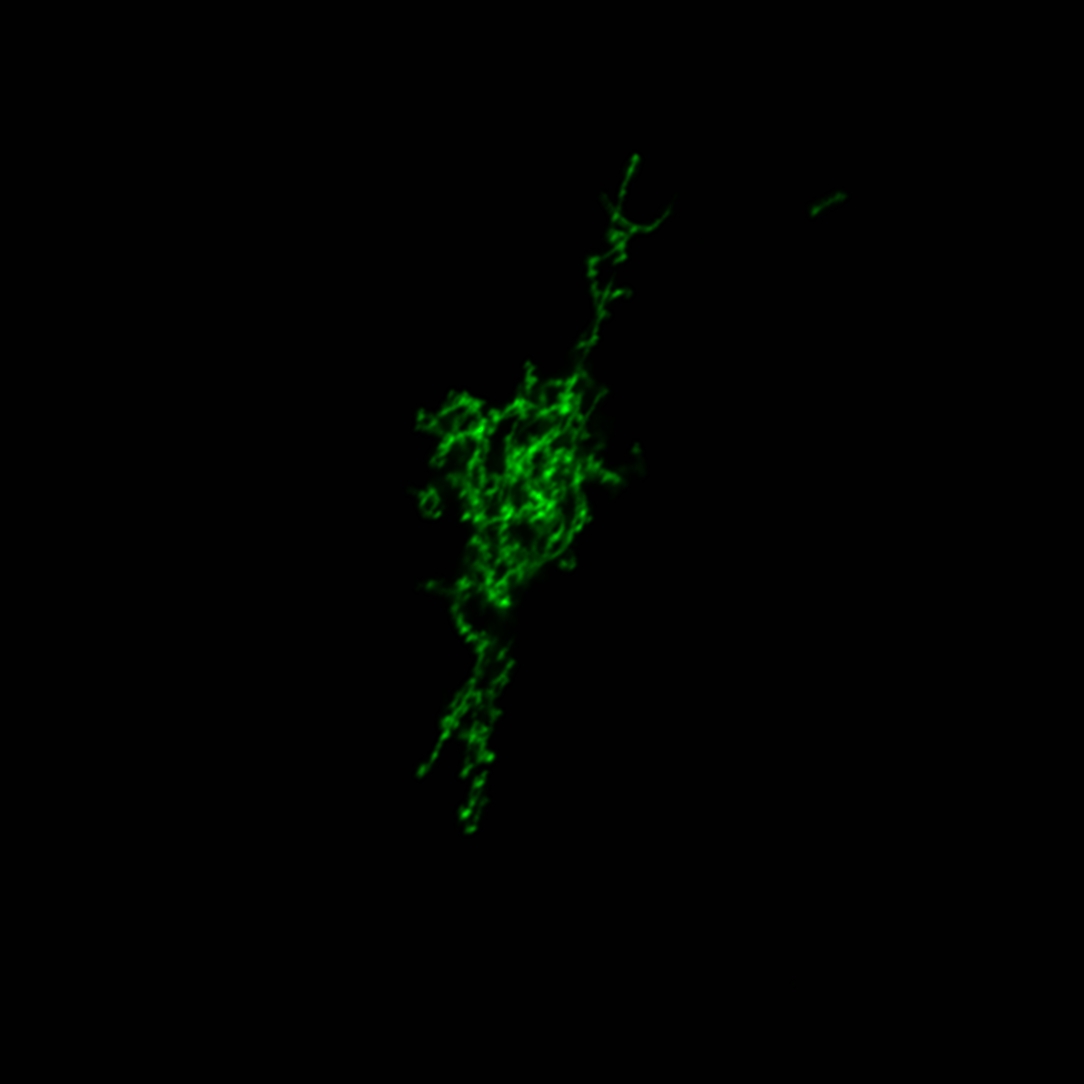

Supplement: S1 File — (ZIP) [file ppat.1012210.s001.zip › Figure 5D default mode MoFim1-GFP+Ca2+/MoFim1-GFP+Ca2+_c2.jpg]

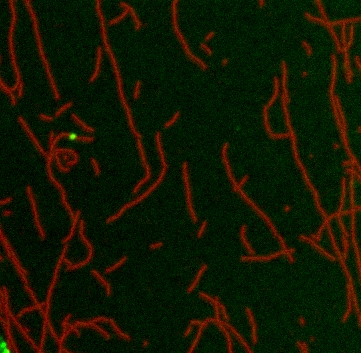

Supplement: S1 File — (ZIP) [file ppat.1012210.s001.zip › Figure 5E best fit mode MoFim1-GFP/Figure 5 E-Image Export-03_c1+2.jpg]

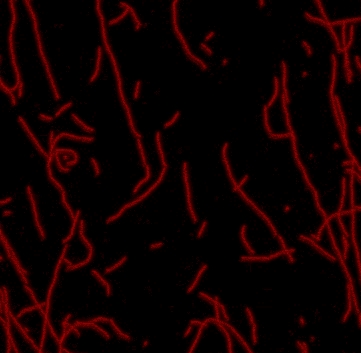

Supplement: S1 File — (ZIP) [file ppat.1012210.s001.zip › Figure 5E best fit mode MoFim1-GFP/Figure 5 E-Image Export-03_c1.jpg]

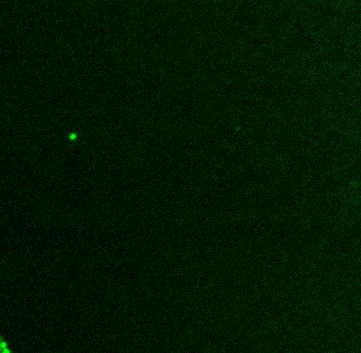

Supplement: S1 File — (ZIP) [file ppat.1012210.s001.zip › Figure 5E best fit mode MoFim1-GFP/Figure 5 E-Image Export-03_c2.jpg]

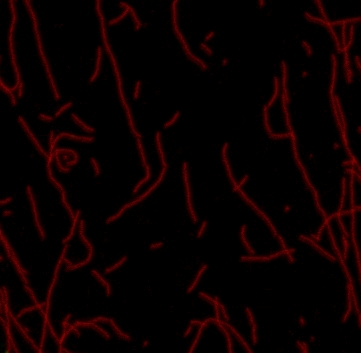

Supplement: S1 File — (ZIP) [file ppat.1012210.s001.zip › Figure 5E default mode MoFim1-GFP/Figure 5 E-Image Export-01_c1+2.jpg]

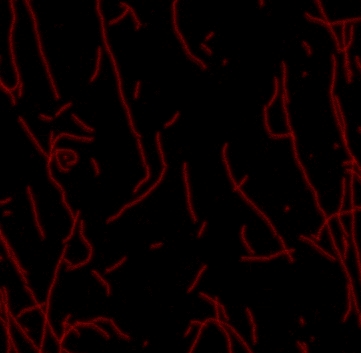

Supplement: S1 File — (ZIP) [file ppat.1012210.s001.zip › Figure 5E default mode MoFim1-GFP/Figure 5 E-Image Export-01_c1.jpg]

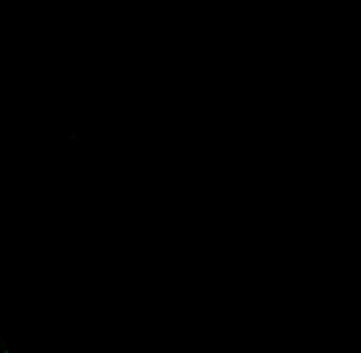

Supplement: S1 File — (ZIP) [file ppat.1012210.s001.zip › Figure 5E default mode MoFim1-GFP/Figure 5 E-Image Export-01_c2.jpg]

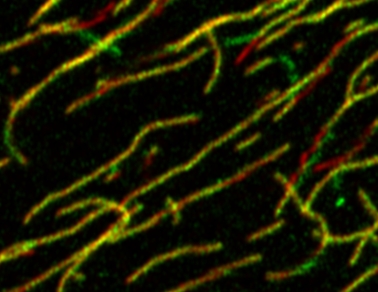

Supplement: S1 File — (ZIP) [file ppat.1012210.s001.zip › Figure 5F best fit mode ABD1-GFP-Image/ABD1-GFP-Image Export-04_c1+2.jpg]

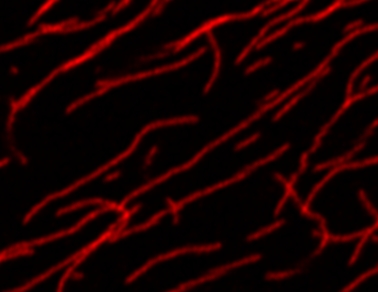

Supplement: S1 File — (ZIP) [file ppat.1012210.s001.zip › Figure 5F best fit mode ABD1-GFP-Image/ABD1-GFP-Image Export-04_c1.jpg]

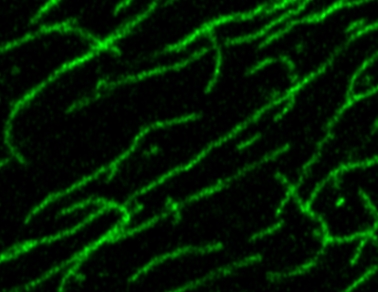

Supplement: S1 File — (ZIP) [file ppat.1012210.s001.zip › Figure 5F best fit mode ABD1-GFP-Image/ABD1-GFP-Image Export-04_c2.jpg]

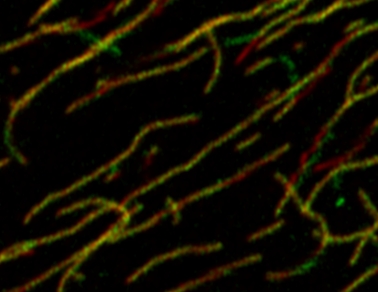

Supplement: S1 File — (ZIP) [file ppat.1012210.s001.zip › Figure 5F default mode ABD1-GFP/ABD1-GFP_c1+2.jpg]

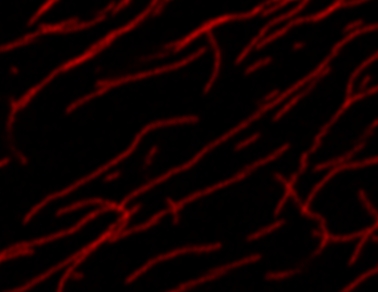

Supplement: S1 File — (ZIP) [file ppat.1012210.s001.zip › Figure 5F default mode ABD1-GFP/ABD1-GFP_c1.jpg]

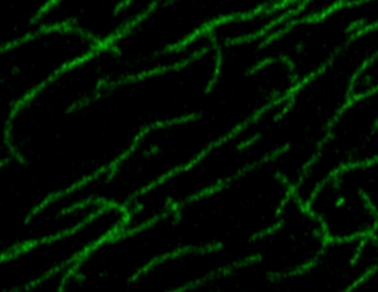

Supplement: S1 File — (ZIP) [file ppat.1012210.s001.zip › Figure 5F default mode ABD1-GFP/ABD1-GFP_c2.jpg]

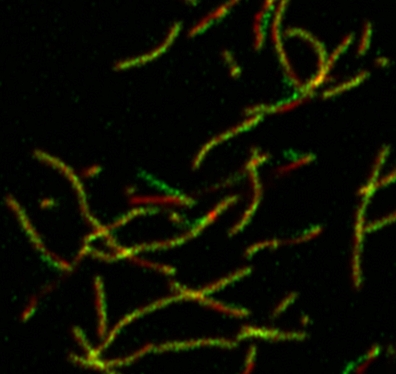

Supplement: S1 File — (ZIP) [file ppat.1012210.s001.zip › Figure 5G best fit mode ABD2-GFP-Image/ABD2-GFP-Image Export-01_c1+2.jpg]

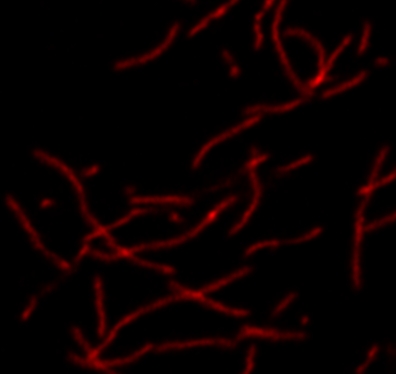

Supplement: S1 File — (ZIP) [file ppat.1012210.s001.zip › Figure 5G best fit mode ABD2-GFP-Image/ABD2-GFP-Image Export-01_c1.jpg]

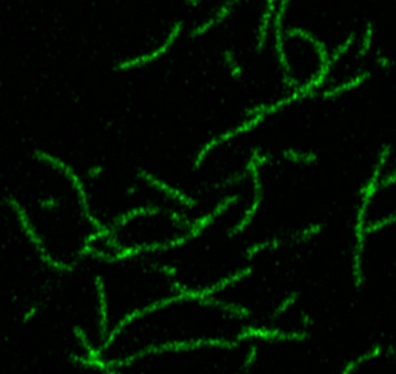

Supplement: S1 File — (ZIP) [file ppat.1012210.s001.zip › Figure 5G best fit mode ABD2-GFP-Image/ABD2-GFP-Image Export-01_c2.jpg]

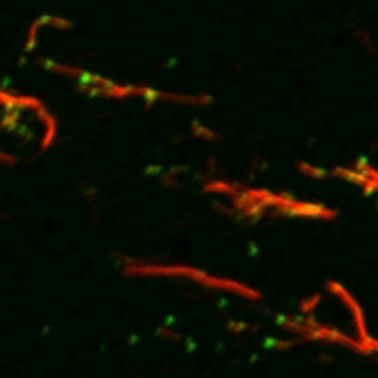

Supplement: S1 File — (ZIP) [file ppat.1012210.s001.zip › Figure 5H best fit mode EF-ABD1-GFP-Image/EF-ABD1-GFP-Image Export-02_c1+2.jpg]

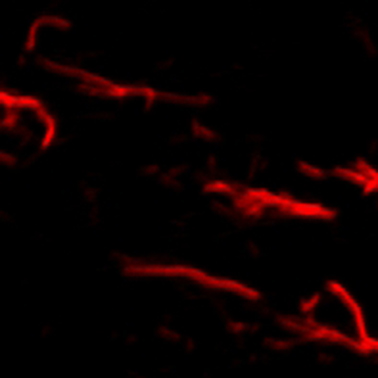

Supplement: S1 File — (ZIP) [file ppat.1012210.s001.zip › Figure 5H best fit mode EF-ABD1-GFP-Image/EF-ABD1-GFP-Image Export-02_c1.jpg]

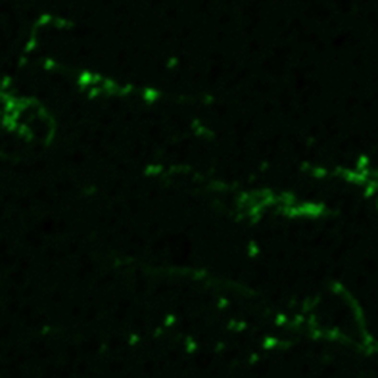

Supplement: S1 File — (ZIP) [file ppat.1012210.s001.zip › Figure 5H best fit mode EF-ABD1-GFP-Image/EF-ABD1-GFP-Image Export-02_c2.jpg]

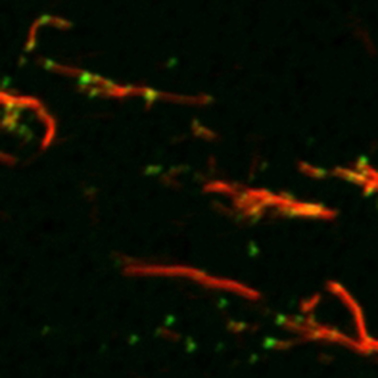

Supplement: S1 File — (ZIP) [file ppat.1012210.s001.zip › Figure 5H default mode EF-ABD1-GFP/EF-ABD1-GFP_c1+2.jpg]

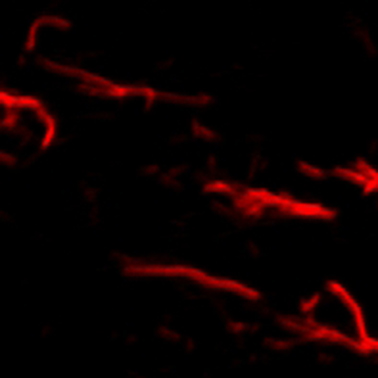

Supplement: S1 File — (ZIP) [file ppat.1012210.s001.zip › Figure 5H default mode EF-ABD1-GFP/EF-ABD1-GFP_c1.jpg]

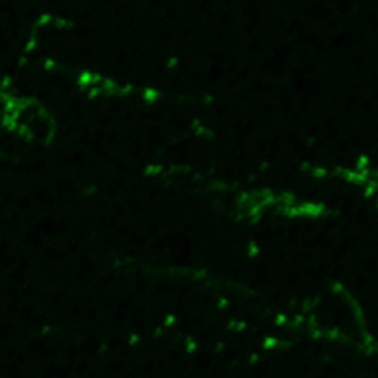

Supplement: S1 File — (ZIP) [file ppat.1012210.s001.zip › Figure 5H default mode EF-ABD1-GFP/EF-ABD1-GFP_c2.jpg]

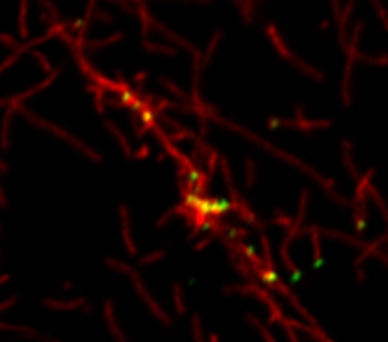

Supplement: S1 File — (ZIP) [file ppat.1012210.s001.zip › Figure 5I best fit mode EF-ABD2-GFP-Image/EF-ABD2-GFP-Image Export-03_c1+2.jpg]

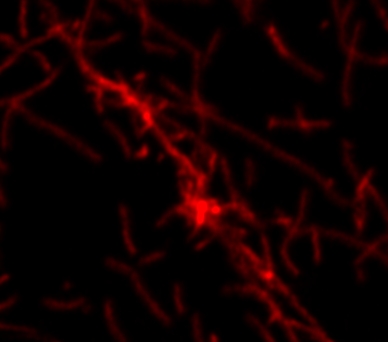

Supplement: S1 File — (ZIP) [file ppat.1012210.s001.zip › Figure 5I best fit mode EF-ABD2-GFP-Image/EF-ABD2-GFP-Image Export-03_c1.jpg]

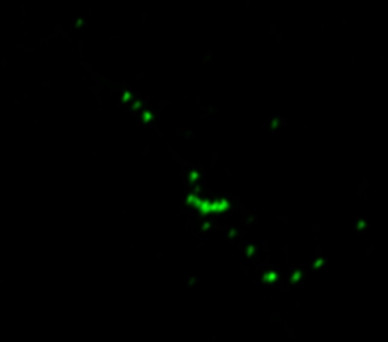

Supplement: S1 File — (ZIP) [file ppat.1012210.s001.zip › Figure 5I best fit mode EF-ABD2-GFP-Image/EF-ABD2-GFP-Image Export-03_c2.jpg]

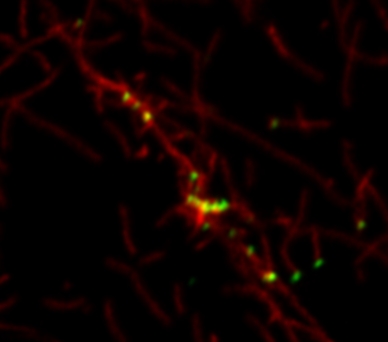

Supplement: S1 File — (ZIP) [file ppat.1012210.s001.zip › Figure 5I default mode EF-ABD2-GFP-Image EF-ABD2-GFP/EF-ABD2-GFP_c1+2.jpg]

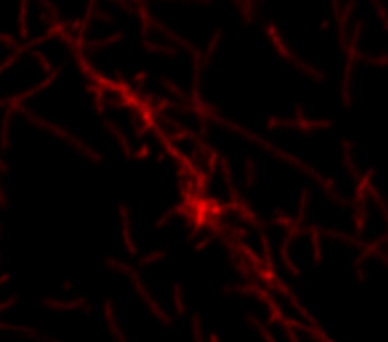

Supplement: S1 File — (ZIP) [file ppat.1012210.s001.zip › Figure 5I default mode EF-ABD2-GFP-Image EF-ABD2-GFP/EF-ABD2-GFP_c1.jpg]
